# Opinion Formation by Social Influence: From Experiments to Modeling

**DOI:** 10.1371/journal.pone.0140406

**Published:** 2015-10-30

**Authors:** Andrés Chacoma, Damián H. Zanette

**Affiliations:** Consejo Nacional de Investigaciones Científicas y Técnicas, Centro Atómico Bariloche and Instituto Balseiro, Comisión Nacional de Energía Atómica, 8400 San Carlos de Bariloche, Río Negro, Argentina; Universidad Carlos III de Madrid, SPAIN

## Abstract

Predicting different forms of collective behavior in human populations, as the outcome of individual attitudes and their mutual influence, is a question of major interest in social sciences. In particular, processes of opinion formation have been theoretically modeled on the basis of a formal similarity with the dynamics of certain physical systems, giving rise to an extensive collection of mathematical models amenable to numerical simulation or even to exact solution. Empirical ground for these models is however largely missing, which confine them to the level of mere metaphors of the real phenomena they aim at explaining. In this paper we present results of an experiment which quantifies the change in the opinions given by a subject on a set of specific matters under the influence of others. The setup is a variant of a recently proposed experiment, where the subject’s confidence on his or her opinion was evaluated as well. In our realization, which records the quantitative answers of 85 subjects to 20 questions before and after an influence event, the focus is put on characterizing the change in answers and confidence induced by such influence. Similarities and differences with the previous version of the experiment are highlighted. We find that confidence changes are to a large extent independent of any other recorded quantity, while opinion changes are strongly modulated by the original confidence. On the other hand, opinion changes are not influenced by the initial difference with the reference opinion. The typical time scales on which opinion varies are moreover substantially longer than those of confidence change. Experimental results are then used to estimate parameters for a dynamical agent-based model of opinion formation in a large population. In the context of the model, we study the convergence to full consensus and the effect of opinion leaders on the collective distribution of opinions.

## Introduction

The study of social interactions in human populations aims at providing a scientifically sound description of what is probably the most intricate and significant aspect of human behavior, namely the dynamics of relations between our equals. It would be difficult to overestimate the relevance that a systematic characterization of social processes, and their underlying mechanisms, has in the understanding of our own nature as members of a community. Moreover, this interest reaches vast domains outside basic science, as it points to questions which are crucial to all kinds of social organization and planning –from the level of everyday interpersonal contacts, to global strategies in politics and economy.

Opinion formation is a favorite theme in the quantitative study of social phenomena, first, because of the interest in predicting collective behavior on the basis of personal stances and their mutual influence. In contrast with decision making –a related class of processes which is however associated with episodes localized in time– opinion formation involves a progressive evolution that, at the level of single individuals, may persist indefinitely as personal opinions change under various effects. This specific feature makes opinion formation a candidate for dynamical modeling, based on the mathematical representation of individual opinions and of the mechanisms of social influence that drive their changes. Moreover, this kind of models can readily be extended to social phenomena which, like opinion formation, are governed by the interaction between suitably defined agents. Prominent examples are, among others, the adoption of cultural traits [1], language evolution [2], and technological innovation [3].

During the last two decades, a multitude of models of opinion formation have been advanced in the literature [4]. A feature common to their vast majority is the very stylized, almost metaphoric approximation to the social phenomenon under study and, at the same time, the close relation to well-known physical systems. For instance, models where opinions have discrete values –representing distinct choices or options, as in an election– have typically been shaped by analogy with spin systems, with social influence represented by some kind of interaction between spins [5–7]. The dynamics of continuous opinions –as in the evaluation of a measurable magnitude– have in turn been modeled as kinetic processes governed by transport equations [8]. Populations of moving individuals have also been represented as kinetic physical systems [9].

The powerful tools of equilibrium and non-equilibrium statistical mechanics provide extremely efficient approaches to deal with these physics-inspired social models. In many cases, it was possible to obtain exact and approximate analytical solutions and/or robust numerical results for various kinds of interaction mechanisms and diverse topologies [4]. At the same time, and perhaps not unexpectedly, the connection between models and real-life social systems became thinner. In his “call for closer link with reality” [10], Sobkowitz reviews an extensive bibliography on opinion formation models, highlighting the fact that only a tiny fraction establishes explicit quantitative connections with real-life data, going beyond hand-waving references to social systems. Systematic links between models and genuine measurements seem to have been assessed only in a few instances, such as for electoral results [11–13] and collaborative webpage editing [14, 15].

Only very recently the same scientific community that developed the corpus of theoretical modeling of opinion formation referred to above has undertaken the design and realization of controlled experiments aimed at complementing and giving empirical support to the models [16–18]. The underlying idea in the domain of experimental psychology [19, 20] consists in quantifying the change of the opinion given by a subject on a specific matter as the result of an event of exposure to a different opinion. On the basis of the same idea, we report in this paper the results of a variant of a recently proposed experiment [18] where not only opinion changes, but also variations in the confidence that subjects assign to their opinions, are quantified and recorded. Besides comparing our results with the previous version of the experiment –with which we find significant similarities and differences– our focus is put in obtaining realistic estimations for parameters that can later be used to feed opinion formation models. In particular, we are interested in evaluating the degree of influence that the exposure to contrasting information can have on the revision of opinion, and how this influence is modulated by the respective confidence levels. We use the experimental outcome to determine parameters for an agent-based theoretical model of opinion formation driven by the mutual influence of individuals in a population, and run several illustrative simulations that also include “opinion leaders.” Analytical results on the evolution and stationary distribution of opinions for the model are also reported.

## Materials and Methods

### Experiment

During the experiment, which was performed in the presence of a supervisor, each subject answered 20 questions, successively appearing on a computer screen. All questions could be given precise quantitative answers, approachable through common knowledge and/or common sense. However, typical subjects were expected to have only an approximate idea of such precise values, so that their answers should emerge from educated guesses. If applicable, the question prescribed in which units the answer should be given. The 20 questions (listed in the S1 Text) were the same for all subjects, but they were sorted at random for each subject to avoid possible systematic correlations between successive answers. The questions covered a broad thematic variety, and they were formulated keeping in mind that the prospective subjects were long-term inhabitants of Argentina (see below).

In the first round, the subject was asked to consecutively write on the computer the answer to each question and, at the same time, to score confidence on the given answer on a scale from 0 to 5 (less to more confidence). In the second round, which took place immediately after the first round ended, the 20 questions were presented again and in the same order. Now, however, the subject was provided with additional information, consisting of a reference answer for each question. In one half of the cases (class A questions) it was stated that the reference answer had been given by a previous individual participant, and the confidence score was also informed. In the other half (class B questions) it was stated that the reference answer was the average of a group of previous participants, in which case no confidence score was given. Actually, the reference answers were generated by drawing a random value that did not differ from the true answer by more than 20%. For class A questions, confidence scores were drawn uniformly at random between 0 and 5. In all cases, the previous answer and confidence score given by the subject were also presented. The subject was asked to answer each question and to score confidence once more, possibly revising the values given in the first round. The computer automatically assigned an anonymous identification code to each subject, which was recorded together with the question number and the two answers and scores.

The experiment was performed on a group of 85 undergraduate students from science and engineering courses of two public universities in Argentina (Universidad Nacional de San Luis and Universidad Nacional de Cuyo). The educational background was thus expected to be reasonably homogeneous. Ages and genders were recorded with the subjects’ consent, but not used in the analysis of the results. Each subject was individually instructed on the experimental procedure, and informed that the experiment was aimed at studying “information processing by humans,” without explicit mention to “opinion formation” and “social influence.” Participation did not contemplate monetary reward. In the S1 Table, we provide the raw data from the experiment.

### Ethics statement

The aims and procedures of the experiment were approved by the Ethics Committee of Instituto Balseiro, Universidad Nacional de Cuyo. All subjects provided written consent for participation.

### Data processing

Various empirical observations of educated guesses by groups of people in different controlled situations [21], including the previous version of the present experiment [18], suggest that the set of quantitative answers to a given question should obey a lognormal distribution –or, equivalently, that the logarithms of the answers are normally distributed. However, a rigorous statistical (Kolmogorov-Smirnov) test of our experimental results does not support this assumption, although the logarithms of the answers do follow a bell-shaped, short-tailed, approximately symmetric distribution (see S1 Fig). It is nevertheless advantageous to work with the logarithms of the answers instead of their original values, as we explain in the following paragraph. Thus, for each answer *a*, we introduce *r* = log *a* as the relevant experimental datum, which for brevity we call the “opinion” associated with that answer.

Although not strictly a Gaussian, the bell-shaped distribution of opinions can be adequately described by its mean value and standard deviation. Moreover, the difference of two opinions –which coincides with the logarithm of the ratio between the respective answers– is independent of the units in which the answers are expressed. This makes it particularly convenient to quantify social influence by means of opinion differences, as the quantification becomes independent of the scale of the answers, thus allowing a direct comparison between the results for different questions. Irrespectively of the choice of units, however, the relative dispersion of opinions can still vary between questions. If necessary, this dispersion can be normalized by scaling the opinions of each question by their standard deviation, as done in S1 Fig.

Comparison of individual opinions with their mean value and standard deviation for each question was used to detect outlier answers. We identified as outliers those opinions differing from the mean value by more than three times the standard deviation. From the total of 85 × 20 = 1700 answers, 56 were discarded as outliers. The analysis of results was performed on the remaining 1644 answers, 804 corresponding to questions of class A, and 840 to class B.

To characterize the change of opinion on a given question, from *r* in the first round to *r*′ in the second round, and with respect to the opinion *r*_*R*_ corresponding to the reference answer presented in the second round, we first compute the initial opinion difference *δr* = *r* − *r*_*R*_ and the opinion change Δ*r* = *r*′ − *r*. We define the influence factor *I* as

$$\begin{array}{c} I=-\frac{\Delta r}{\delta r}=\frac{r^{\prime }-r}{r_{R}-r} \end{array}$$ (1)

This quantity equals zero when the opinion does not change between the two rounds (*r*′ = *r*), and one when the reference opinion is adopted (*r*′ = *r*_*R*_). If the new opinion *r*′ is a compromise between *r* and *r*_*R*_, then 0 < *I* < 1. We stress that the influence factor is independent not only of the units in which the answers are given, but also of the dispersion of the set of opinions corresponding to a given question. The values of *I* can thus be safely compared between questions.

As for the characterization of the confidence levels, we call *c* and *c*′ the scores assigned by the subject to each answer in the first and second round, respectively. When applicable, *c*_*R*_ is the confidence assigned to the reference answer. The three quantities are integer numbers between 0 and 5. Our analysis focuses on the confidence change Δ*c* = *c*′ − *c*, and the confidence difference with the reference answer *δc* = *c* − *c*_*R*_. Both differences can vary between −5 and 5.

### Theoretical model

We propose an agent-based model for a population of interacting individuals where each agent *i* is endowed with an opinion *r*_*i*_ and a confidence level *c*_*i*_. Both attributes evolve as the result of interactions. Each interaction event –which we conceive as being similar to a realization of our experiment for any given question of class A– involves an active agent *i* whose opinion and confidence can vary by confrontation with the attributes of a reference agent *j*. At each event the two agents are drawn at random from the population.

According to the definitions introduced in the previous section, we write the updated attributes for agent *i* as

$$\begin{array}{c} r_{i}^{\prime }=r_{i}+I_{ij}\left(r_{j}-r_{i}\right)\;\;\;\;\;c_{i}^{\prime }=c_{i}+\Delta c_{ij} \end{array}$$ (2)

where *r*_*j*_ is the reference opinion. The influence factor *I*_*ij*_ and the confidence change Δ*c*_*ij*_ corresponding to the interaction of agents *i* and *j* are chosen on the basis of our experimental results. Our main aim is to characterize the collective evolution of opinion and confidence as successive interactions take place all over the population. We deal with the model both analytically and by means of numerical simulations.

The first of Eq (2) belongs to the class of kinetic models of opinion formation [8, 10] for the time-discrete evolution of continuous opinions under the effect of social influence. In contrast with previous realizations of this kind of models [22–25], in our approach the influence factor *I*_*ij*_ is a random variable drawn at each interaction event from a distribution derived from the experimental results.

## Results

### Experimental Results

The pair of answers given by each subject to each question falls in one of three distinct categories. The first category corresponds to the answers where the subjects keep their opinion unchanged between rounds (influence factor *I* = 0), or even react to the reference opinion by enhancing the difference with it (*I* < 0). At the other end, the subjects exactly adopt the reference opinion (*I* = 1), or overreact by adopting an even more distant opinion (*I* > 1). In the third category, the second-round opinion is a compromise between the initial opinion and the reference opinion (0 < *I* < 1). These three categories have also been identified in the previous version of the experiment [18], though no cases with *I* < 0 or *I* > 1 were reported. In our realization, extreme reactions with *I* < 0 or *I* > 1 amounted to 3.9% of the total set of answers.

**Fig 1:**
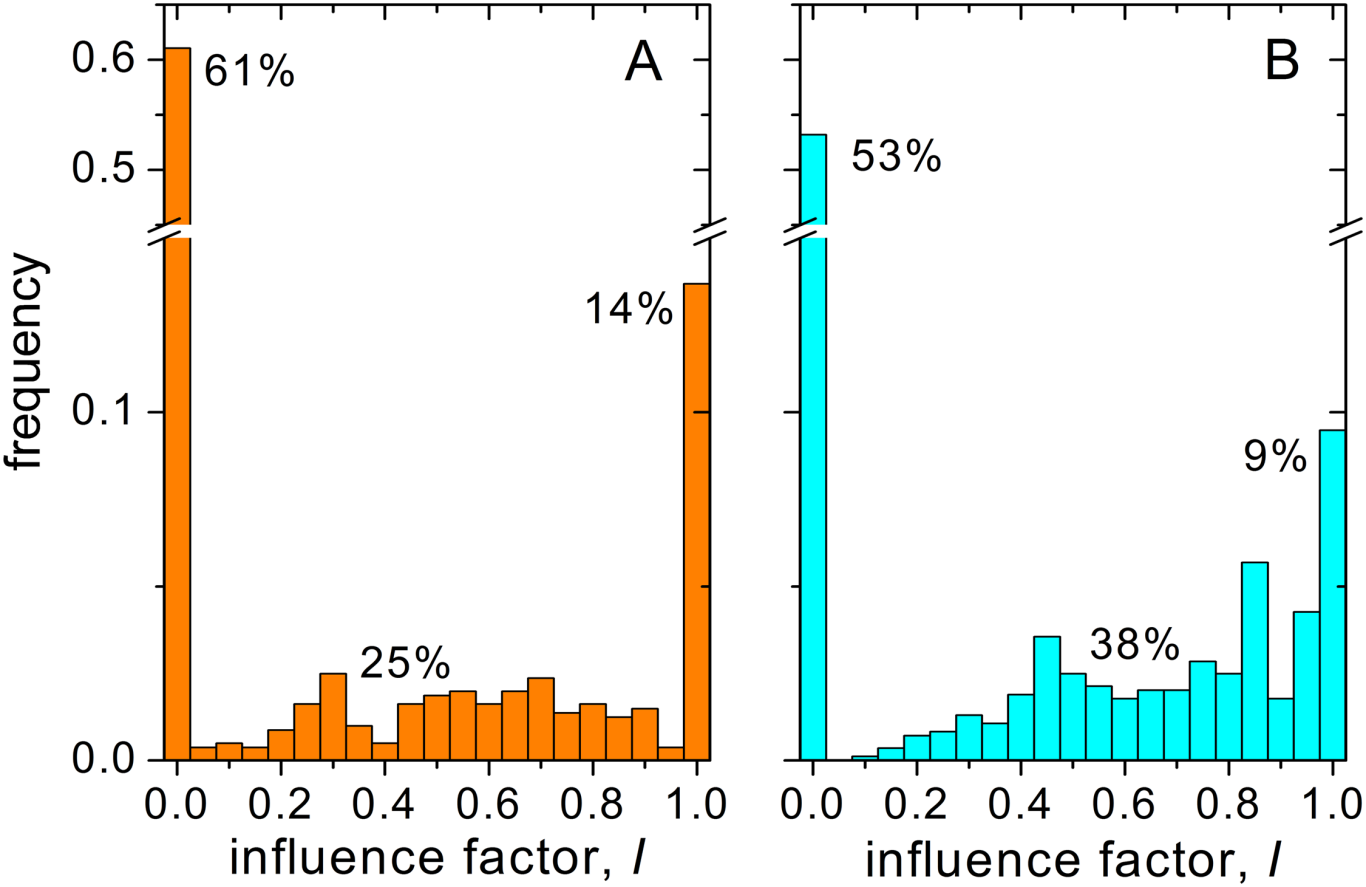
Distribution of the influence factor. The histograms show the frequency for the influence factor *I* recorded in the experiment. Percentages stand for the frequencies of answers (rounded to integer values) where the opinion is kept unchanged (*I* = 0), where the reference opinion is adopted (*I* = 1), and where a compromise is reached (0 < *I* < 1). The columns corresponding to *I* = 0 and *I* = 1 respectively incorporate the answers with *I* < 0 and *I* > 1. A: Individual influence (class A questions). B: Group influence (class B questions).

Fig 1 shows histograms of the values obtained for *I* in the two classes of questions (A and B, respectively with individual and group influence). For the sake of compactness, the histograms incorporate to the columns assigned to *I* = 0 and *I* = 1 all the answers with *I* ≤ 0 and *I* ≥ 1, respectively. The frequency with which the initial opinion is kept unchanged (*f*_*K*_, given by the height of the columns at *I* = 0) is above 50% for both classes. Keeping the opinion is therefore the most common recorded behavior [18]. In turn, adopting the reference opinion is the category with the lowest frequency, *f*_*A*_ = 14% and 9% in the respective classes. Finally, the frequency of compromise opinions reaches *f*_*C*_ = 25% and 38%. The corresponding values of *I* are more or less scattered in the interval (0,1), although a slight preference for large values seems to occur, with an average close to *I* = 0.6 in both classes.

In spite of the qualitative similarity between the results for the two classes, a Kolmogorov-Smirnov test shows that their difference is statistically highly significant (*p* < 0.001). The difference suggests that in the case of individual influence subjects tend to embrace more extremist attitudes toward the potential influence on their opinions, either resisting or welcoming the influence with higher frequency. For group influence, on the other hand, compromise is considerably more frequent, but still does not represent the dominant behavior.

**Fig 2:**
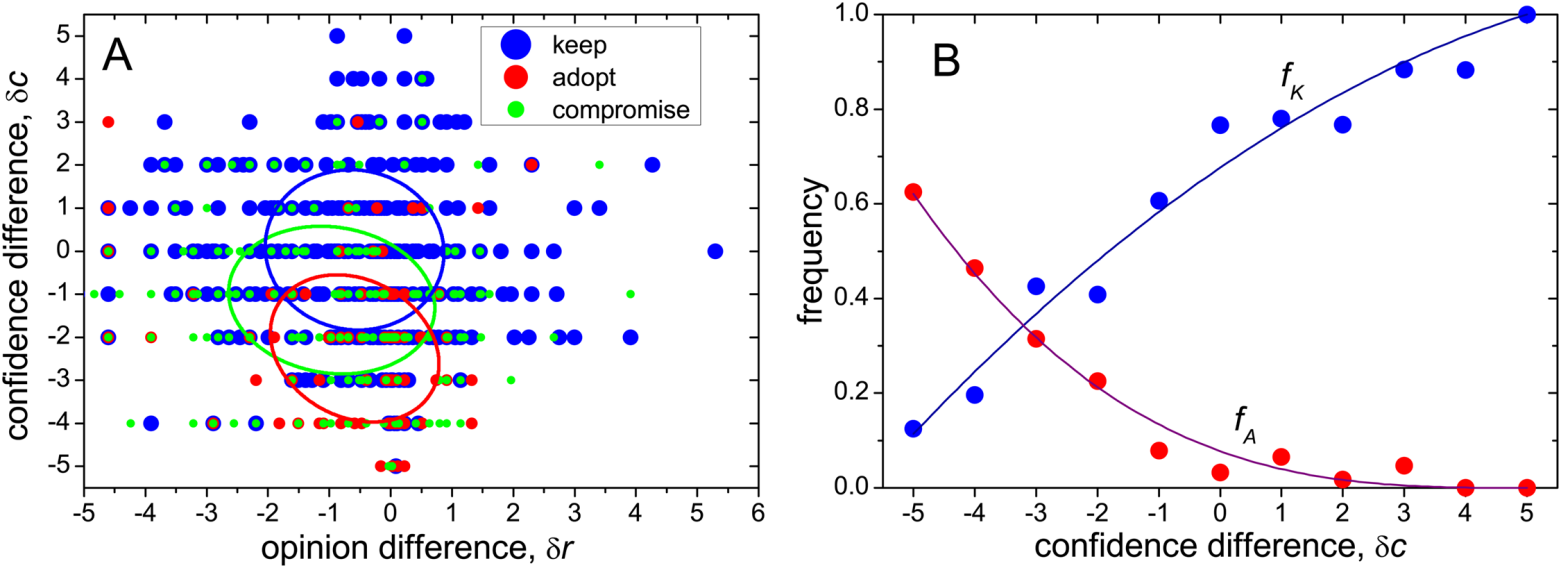
Distribution of answers according to opinion and confidence differences. A: Each dot in the plot corresponds to a pair of answers, with coordinates given by the corresponding opinion and confidence differences, *δr* and *δc*. Colors and sizes code the three answer categories, as indicated in the legend. Ellipses give the standard-deviation intervals around the mean value of each category, following the same color coding. B: The frequency of answers corresponding to two categories (keep and adopt) as a function of the confidence difference. Curves are polynomial fittings to the data: *f*_*K*_ = 1 − 0.04(5 − *δc*) − .005(5 − *δc*)^2^, and *f*_*A*_ = 6.2 × 10^−4^(5 − *δc*)^3^. The least-square regression coefficients are *R* ^2^ = 0.96 and 0.98, respectively.

In order to analyze the connection between influence and confidence we now focus on questions of class A, for which a reference confidence level is available. We first study how the three answer categories (keep, adopt, and compromise) are determined by the opinion difference *δr* = *r* − *r*_*R*_ and the confidence difference *δc* = *c* − *c*_*R*_. Each dot in Fig 2A represents an answer pair, with its category coded by a color (different sizes are used to discern between overlapping dots). Their coordinates are given by the corresponding *δr* and *δc*. The three ellipses are centered at the average coordinates of each category, and their axes are oriented along the directions of maximal and minimal variance, with semi-diameters given by the respective standard deviations.

While the distribution of answers on the (*δr*, *δc*)-plane is very disperse, there is a statistically significant mutual separation between the ellipses along the confidence difference axis. As may be expected, large and small values of *δc* –where the subject confidence is respectively high and low as compared with the reference– are correspondingly related to keeping and adopting opinions. Compromising opinions occupy the intermediate range. Correlation between different categories and the opinion difference, on the other hand, is hardly significant. This fact suggests that, interestingly, comparison with the reference opinion has negligible consequence on the kind of response to influence.

The correlation between answer categories and confidence difference is qualitatively the same as obtained in the previous version of the experiment [18]. However, in that version the distribution of answers in the (*δr*, *δc*)-plane was used to construct an “influence map” with three well-separated regions, one corresponding to each category. This map provided then the basis for formulating a model of opinion formation by social influence. In contrast, according to our results there is a major overlapping between the regions associated with each category, so that the construction of such a map cannot be justified. The correspondence between an answer’s category and its position in the (*δr*, *δc*)-plane is at most of probabilistic nature.

A more transparent characterization of the distribution of answers by category in terms of the confidence difference is given by the measurement of the frequency of the three categories as a function of *δc*. Taking advantage of the fact that the confidence differences are always integer numbers, this can be done for every value of *δc*, between −5 and 5. Fig 2B shows the results for two categories (keep, *f*_*K*_; adopt, *f*_*A*_). The frequency of the remaining category (compromise, *f*_*C*_ = 1 − *f*_*K*_ − *f*_*A*_) is just the complement of the other two. Results clearly show the inverse trend of *f*_*K*_ and *f*_*A*_, with the former growing and the latter decreasing as the subject confidence increases with respect to the reference. In particular, for the maximal confidence difference, *δ*_*c*_ = 5, the frequencies reach the extreme values *f*_*K*_ = 1 and *f*_*A*_ = 0. The curves in Fig 2B are polynomial fittings to the data, used below in our numerical simulations.

**Fig 3:**
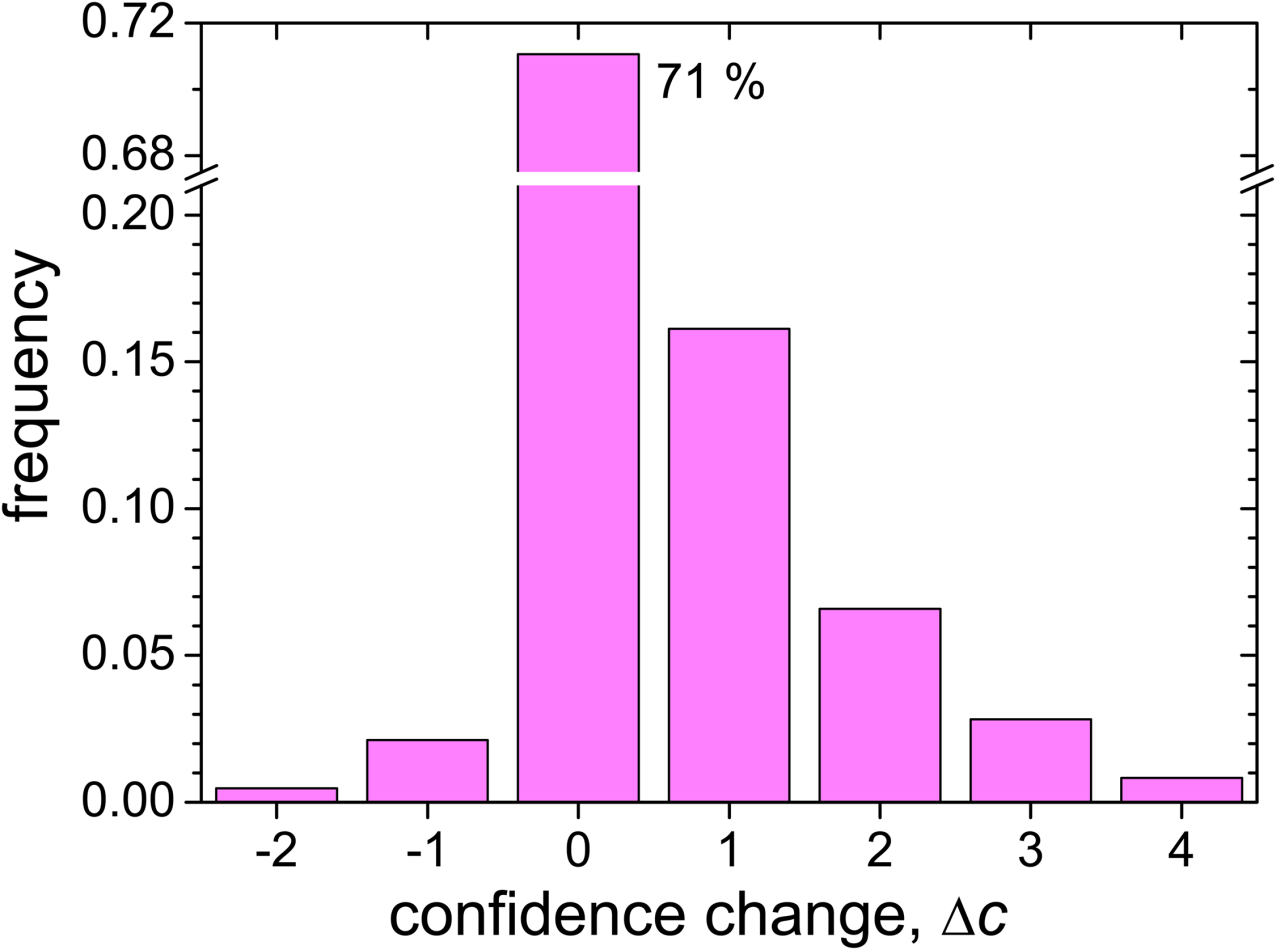
Distribution of changes in the confidence level. The histogram shows the frequencies for the confidence change between rounds, Δ*c* = *c*′ − *c*, for class A questions (see also S2 Table). The frequency is exactly equal to zero for Δ*c* < −2 and Δ*c* > 4. By far, the most frequent value is Δ*c* = 0, with about 71% of the cases. The distribution is biased towards positive values, with a total of 26% and 3% of the cases for Δ*c* > 0 and Δ*c* < 0, respectively.

Finally, we turn the attention to the confidence change between rounds, Δ*c* = *c*′ − *c*. Fig 3 shows a histogram for Δ*c* from the answer pairs for class A questions. As for the opinions, the dominant behavior with confidence is a conservative stance: in 71% of the answers, subjects do not modify their confidence, in spite of their interaction with the reference opinion. Moreover, the distribution is heavily biased toward positive values, meaning that when the confidence level changes it usually grows. This occurs in 26% of the answers, with a preference for small positive confidence changes. Only a 3% corresponds to decreasing confidence. The S2 Table gives the observed frequency for each Δ*c*, which we use later in modeling the evolution of confidence.

A remarkable result for the confidence change is that Δ*c* shows little correlation with both the opinion difference *δr* and the opinion change Δ*r*. The respective Pearson’s correlation coefficients are *ρ* = −0.02 and 0.05. The correlation between Δ*c* and the influence factor *I* gives *ρ* = 0.09, still a small value. Therefore, we conclude that modifications in the level of confidence if to a large extent quantitatively independent of opinion.

On the other hand, Δ*c* shows a sizable correlation with the initial confidence level *c*, with a correlation coefficient *ρ* = −0.49. This fact is in turn closely related to a high correlation between Δ*c* and the confidence difference *δc*, with *ρ* = −0.47. However, the correlation between Δ*c* and *c* is an artifact originating in the fact that the confidence level is limited to vary between 0 an 5. Indeed, this implies that from a given initial confidence *c* the resulting confidence change is necessarily confined to vary between −*c* and 5 − *c*. Assuming a uniform distribution of answers within this interval yields a correlation between Δ*c* and *c* with *ρ* = −0.7. This effect completely explains the high correlation observed in the experiment.

### Analytical and Numerical Results

#### The evolution of confidence

As shown by our experimental results, confidence changes between the two rounds of questions bear little correlation with any of the other quantities measured during the experiment. At the level of modeling it thus seems reasonable to typify the evolution of confidence as an autonomous process, in particular, not influenced by the opinion evolution.

In a series of interaction events involving a given active agent *i* and generic reference agents *j*, the confidence *c*_*i*_ evolves as a consequence of the successive confidence changes Δ*c*_*ij*_ (see the second of Eq (2)). According to our experimental results, we assume that the probability of any given Δ*c*_*ij*_ is independent of individual opinions and confidence, and can be estimated from the experiment as the observed overall frequency for each possible confidence change (Fig 3; S2 Table). Moreover, we introduce the simplifying assumption that this estimation is valid not only for the first interaction, but along a continued succession of events. With these elements at hand, it is possible to write evolution equations for the probability of each confidence level as a function of the elapsed number of events. In a large population this probability coincides with the fraction of individuals with each confidence level. Details of the mathematical model are given in the S2 Text.

The solution to the equations show that, after a sufficiently long sequence of interaction events, the distribution of confidence levels over the population reaches a stationary profile, independent of its initial condition. Table 1 gives the distribution of confidence levels measured in the initial round of the experiment, and the stationary values obtained from the theoretical model. We see that, according to the model, almost 90% of the population reaches maximal confidence due to the preferentially positive values of confidence changes. However, negative confidence changes make possible lower confidence levels, although with considerably lower frequencies.

**Table 1:** Frequencies of confidence levels. First line: as measured in the first round of the experiment. Second line: theoretical stationary values.

confidence	0	1	2	3	4	5
**initial (experiment)**	0.086	0.113	0.216	0.400	0.160	0.025
**stationary (theory)**	< 0.001	0.001	0.003	0.022	0.079	0.895

From the solution, it is also possible to evaluate the typical number of events necessary to approach the stationary distribution of confidence levels. Results indicate that the difference between a generic frequency distribution and the stationary distribution approximately halves every 4 events per agent or, equivalently, is reduced by a factor of 10 every 13 events per agent (see S2 Text). Thus, the convergence of confidence levels toward their long-time profile is a rather fast process, in particular by comparison with the evolution of opinions, as we show in the following.

#### The evolution of opinions

In numerical simulations, our model of opinion formation is based on the selection of one of the three categories with the frequencies recorded in the experiment, *f*_*K*_, *f*_*A*_, and *f*_*C*_, according to which the active agent keeps opinion, adopts the reference, or chooses a compromise. Once the active agent *i* and the reference agent *j* have been selected, their confidence difference *δc* = *c*_*i*_ − *c*_*j*_ is calculated. For this value of *δc*, the probabilities of the three categories is computed from the analytical approximations to the corresponding frequencies, quoted in the caption to Fig 2B and plotted as curves in the same figure. A category is then chosen using those probabilities, which determines the value of the influence factor *I*_*ij*_ for this event. In the case of compromise, the value of *I*_*ij*_ is uniformly drawn at random in the interval (0,1). Finally, the influence factor is used to update the active-agent opinion following the first of Eq (2).

The fact that at each interaction event (unless *δc* = 5) there is a finite probability that the active agent changes its opinion to a compromise with the reference implies that the population asymptotically approaches a state of full consensus for long times, with all the agents sharing the same opinion. Assuming as an approximation that all the agents have maximal confidence, it is possible to analytically show that the final consensus opinion coincides with the average value of the initial opinions (see S3 Text). Moreover, by studying the evolution of the standard deviation of opinions, it is possible to estimate the typical number of events per agent necessary to approach consensus. The calculation shows that the standard deviation decreases by a factor of 2 approximately every 16 events per agent or, equivalently, by a factor of 10 every 54 events. Hence, the evolution of opinions is substantially slower than that of confidence.

**Fig 4:**
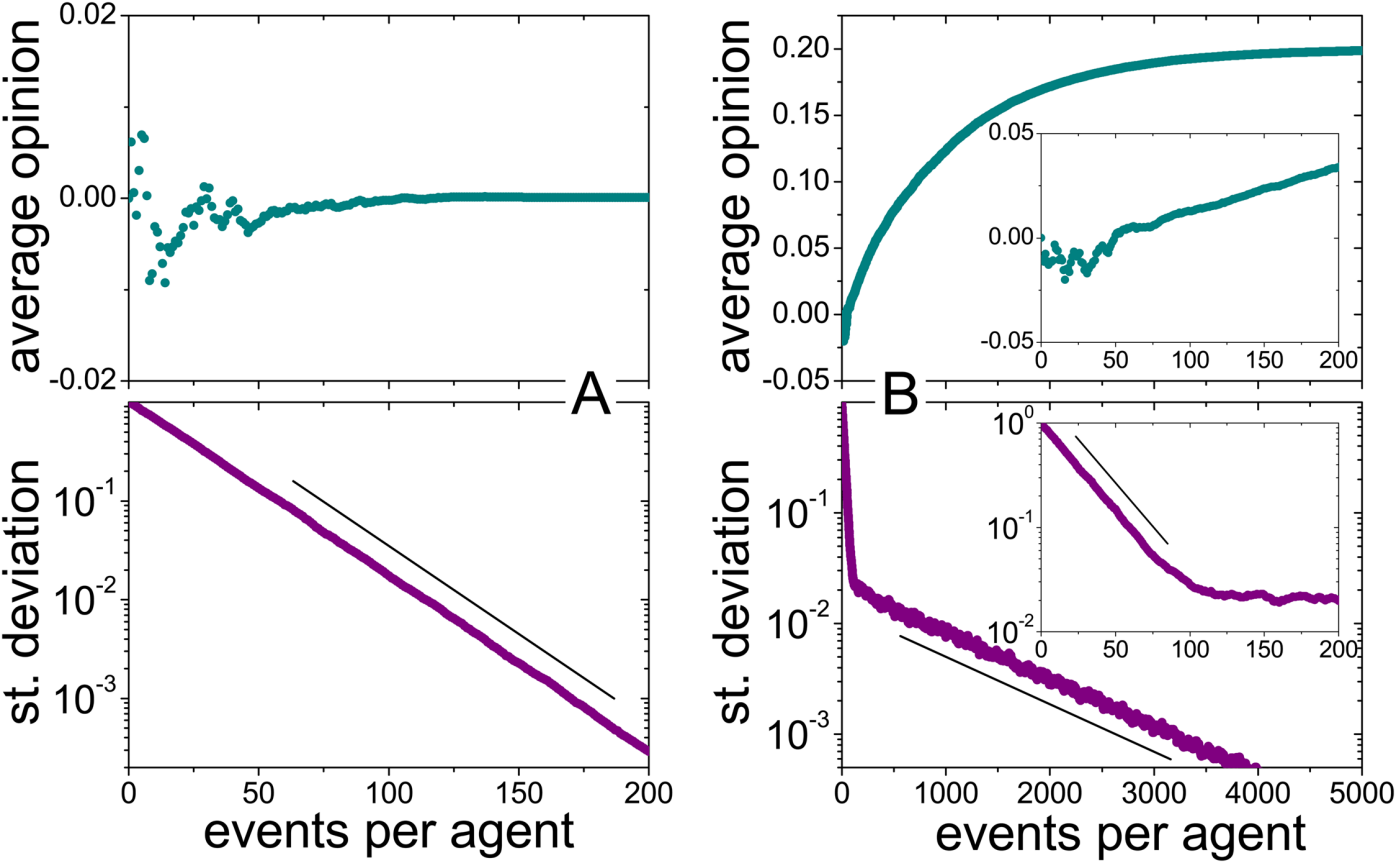
Evolution of the average opinion and the standard deviation. The plots show simulation results for a population of 10^4^ agents, whose initial opinions where normally distributed and whose confidence is distributed as observed in the first round of the experiment. Results are plotted as functions of the number of events per agent. Straight lines stand for the analytical prediction of exponential slopes (see S3 Text). A: Without opinion leaders. B: With one opinion leader, whose opinion is *R* = 0.2. The leader’s influence factor is distributed as for any ordinary agent, but the leader is chosen as a reference 100 times more often than any other agent. The insets are close-ups of the initial evolution stage.

Fig 4A shows simulation results on the evolution of the average opinion and the standard deviation for a population of *N* = 10^4^ agents. Initially, the opinions were normally distributed, with zero mean and unitary standard deviation, while the confidence is distributed as observed in the first round of the experiment. The average opinion shows transient fluctuations with a typical size of order *N* ^− 1/2^ = 0.01, until it settles down to its asymptotic value. The standard deviation decreases in turn exponentially with the slope predicted by the analytical calculation (straight line). A minor deviation from a pure exponential decay is observed at the very first stage, which can be ascribed to the still-evolving confidence distribution.

The rather uninteresting fate of full consensus at the average opinion can be challenged by the presence of one or more agents with special dynamic rules. Here, we consider “opinion leaders” [26–28] which are characterized by their privileged capacity of influencing ordinary agents. An opinion leader is defined by three special features. First, its opinion is fixed (i.e., it is not affected by interaction events) and its confidence is always maximal. Second, the frequency with which it is selected to play the role of reference can be higher than that of an ordinary agent. Third, its influence on the opinion of the active agent (measured by the average influence factor) can also be higher.

Under the influence of a single opinion leader, the population is still attracted toward an asymptotic state of full consensus. However, the final unanimous opinion coincides now with that of the leader. While the interaction between ordinary agents drives opinions to their average, occasional compromise events with the leader’s opinion, which is fixed and highly confident, determine the collective drift of the whole population to ultimate agreement with the leader. If the leader’s influence on each agent –measured by the frequency of their mutual interactions and by the corresponding influence factor– is small to moderate as compared with the effect of the remaining population, the collective evolution of opinions exhibits two well separated stages. First, the opinions initiate a rapid collapse toward their average value, as if the opinion leader were absent. Later on, however, interactions with the leader begin to have a sizable effect. In this second stage, the average opinion drifts toward the leader’s opinion and, at the same time, the decrease of the standard deviation slows down abruptly. The two evolution stages are illustrated by simulation results in Fig 4B. In these simulations, the leader’s influence factor is distributed as for any ordinary agent, but its opinion is chosen as a reference 100 times more frequently, i.e. with probability *α* ≈ 100 *N* ^−1^ = 0.01.

Two or more leaders with different opinions are necessary to drive the population to a stationary state where full consensus is avoided and individual opinions do not coincide. In this state, the opinion of any agent continues to evolve indefinitely, driven by its interaction with the leaders and with other agents with different opinions, but the opinion distribution over the population is statistically invariant. In the S3 Text, we give analytical results for the asymptotic average opinion and the standard deviation in the case of several leaders. Full consensus is possible in this case if and only if all leaders share the same opinion.

**Fig 5:**
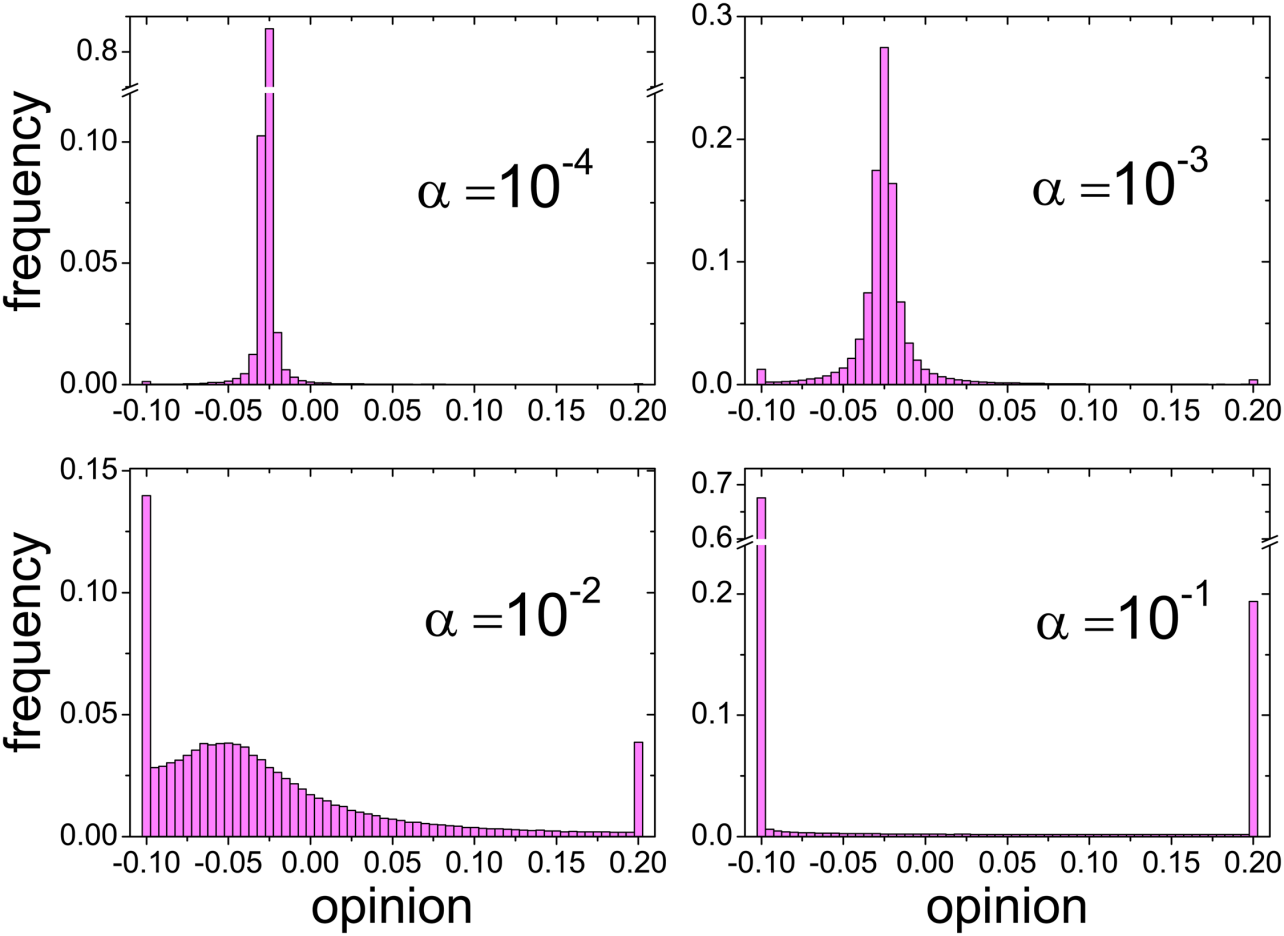
Opinion distributions under the influence of two leaders. The histograms show simulation results for the stationary distribution of opinions in a population of 10^4^ agents, with the same initial condition as in Fig 4. The leaders have fixed opinions *R*_1_ = 0.2 and *R*_2_ = −0.1, and they are chosen as references with frequencies *α*_1_ = *α* and *α*_2_ = 4*α*, respectively. The four panels correspond to different values of *α*, as indicated by the labels. The leaders’ influence is such that, upon interaction with a leader, ordinary agents always adopt the leader’s opinion.

As an illustration, Fig 5 shows simulation results for the stationary distribution of opinions under the influence of two leaders with distinct opinions. Different panels in the figure correspond to different frequencies of interaction with the leaders (although the ratio between the two frequencies is held constant throughout). For small frequencies, the distribution exhibits a narrow profile, with a large fraction of the opinions at or around an intermediate value. Analytical results show that, even for arbitrarily small frequencies, this intermediate value is determined by the leaders’ opinions. The initial opinions of ordinary agents have no effect on it (see S3 Text). As the frequency grows, the distribution becomes broader and flatter. At the same time, sharp peaks appear at the leaders’ opinions [27]. Finally, for sufficiently high frequencies, the distribution shows a bimodal profile where practically all agents share their opinion with either leader. At these interaction frequencies, the population has been polarized by the large, contrasting influence of the two leaders.

## Discussion and Conclusion

We have here presented results on a variant of a recently proposed experiment on opinion formation by social influence [18]. In short, the experiment consisted of a series of events where each subject gave a quantitative answer to a question and then had the opportunity of revising the answer upon exposure to a different (“reference”) opinion on the same question. The subject was also required to evaluate his or her degree of confidence on the two answers. Our focus was put on the quantification of the change from the first to the second answer and of the variation in confidence, as measures of the influence of the reference opinion on the subject. Experimental results were then used to estimate parameters for a dynamical agent-based model of opinion formation, where the collective distribution of opinions in a large population evolves as the result of iterated pair interactions, similar to the individual events of the experiment.

The most frequent instance recorded in the experiment corresponds to no change in the answers and in confidence –in agreement with a comparable result in the original version of the experiment. In almost 60% of the events, the subjects chose not to modify their answers, while their confidence remained the same in more than 70%. This behavior, which may be inscribed in a form of resistance to persuasion by which a person psychologically affirms self-consistency and the sense of control [29], does obviously not contribute to the collective evolution of opinions. However, as long as adoption of the reference opinion and compromise between the initial answer and the reference are also possible, the main effect of stubborn attitudes is a slowing down of the overall process [30]. In round numbers, in the experiment, opinion adoption and compromise respectively corresponded to some 10% and 30% of the events. In the case of compromise, the revised answer was more or less uniformly scattered between the initial answer and the reference.

According to our results, when confidence changed, increments were roughly ten times more frequent than reductions. Remarkably, moreover, confidence changes bore nearly insignificant statistical correlation with all the other quantities involved in each event, such as the difference in opinion and confidence with the reference, and the opinion change. This may be an interesting clue to a generalized reaffirmation attitude [31], irrespective of an objective evaluation of the own opinion and confidence by comparison with the reference. At the level of modeling this observation makes it possible to treat confidence as an attribute with autonomous dynamics. The insertion of our experimental results in a dynamical model showed that iterated interactions lead to a stationary confidence distribution with some 90% of the population at the maximum confidence level after just a few events per agent. Note however that the model assumes that the confidence changes are the same along a whole series of interaction events. Validation of this hypothesis would require further evidence from experiments.

It must be recognized that confidence scoring was based on a qualitatively-defined scale, contrary to the quantitative answers to the questions in the experiment. Scores depended therefore on the interpretation of the scale by each subject and thus did not posses the desirable objectivity. This lack of rigorous definition in the confidence levels may explain in part the low correlation of confidence changes with all the other experimental measurements. The design of a more convincing quantitative characterization of confidence remains a challenging question.

Another aspect inscribed in the problematic of qualitative valuations has to do with the perceived difficulty or ambiguity of each individual question, irrespectively of the fact that it has a precise quantitative answer. Different degrees of difficulty may lead the subject to adopt various strategies when generating a response, and may affect the stance toward the reference answer. The analysis of this attitudinal feature would probably require an independent empirical approach.

In contrast with the original version of the experiment, our results do not suggest a clear-cut relation between response to the reference opinion (keep, adopt, or compromise) and the corresponding difference in opinions and confidence. On the other hand, we have found a highly significant statistical correlation between the frequency of each one of the three categories and the initial difference between subject and reference confidence. We conclude that our results do support a probabilistic prediction of the influence on his or her response from the confidence difference with the reference, although we cannot chart an “influence map” to anticipate behavior in terms of opinions and confidence [18]. On the basis of this observation, we have estimated the parameters of a kinetic-like model for opinion formation, for which we obtained a series of analytical results and run several numerical simulations on the long-term evolution of opinions driven by iterated interaction events. It is important to notice that in our model all agents are in principle equivalent to each other, in the sense that their behavior is controlled by the same probabilistic parameters. This is consistent with the fact that in the experiment we have not analyzed whether different individuals exhibited significantly disparate behavior as for their response to influence. Additionally, in the model we have introduced special agents in the form of “opinion leaders” as a way to explore situations where opinions do not converge to full consensus but develop a nontrivial stationary distribution.

Using the experiment-based parameter estimation, our analytical results reveal, first, that the typical evolution times of opinion formation are substantially longer than those of confidence levels. Second, as shown in the S3 Text, the large disparity in the agents’ response to social influence –quantified by the broad distributions of influence factors shown in Fig 1– plays an important role in the dynamics. Indeed, the standard deviation of the influence factor is involved in both the convergence rate of the opinion distribution and in its stationary dispersion. This points to the fact that including fluctuations in the parameters that weigh social influence is a desirable ingredient in modeling.

An experimental observation that clashes with the basic hypotheses of some opinion formation models is the lack of correlation between response and difference between opinion and reference. In “bounded confidence” models [22–25], opinion compromise is possible only if the original opinions of the interacting agents differ by less than a given threshold. Otherwise, the agents keep their respective opinions. In contrast with this hypothesis, our results show that whether the active agent keeps opinion or makes a compromise, perhaps even adopting the reference opinion, is largely independent on how the two opinions compare. This kind of conflict should serve as an incentive to ground theoretical quantitative studies of opinion formation, and social phenomena in general, on solid empirical evidence.

## Supporting Information

S1 Fig Distribution of opinions measured in the experiment. The histograms incorporate the answers to all questions in each round. The distribution of opinions for each question has been standardized by subtracting their mean value and scaling by their standard deviation. The curves correspond to a normal (Gaussian) distribution with zero mean and unitary standard deviation.
(TIF)

S1 Table Raw data obtained from the experiment. (PDF)

S2 Table Frequencies of confidence changes measured in the experiment. (PDF)

S1 Text List of questions used in the experiment. (PDF)

S2 Text Analytical model for the autonomous evolution of confidence. (PDF)

S3 Text Analytical approximation to the evolution of opinions. (PDF)
