# Supplementary figures and images for: Opinion Formation by Social Influence: From Experiments to Modeling
Source: PLoS One. 2015 Oct 30;10(10):e0140406. doi: 10.1371/journal.pone.0140406 (PMC4627778; doi:10.1371/journal.pone.0140406)

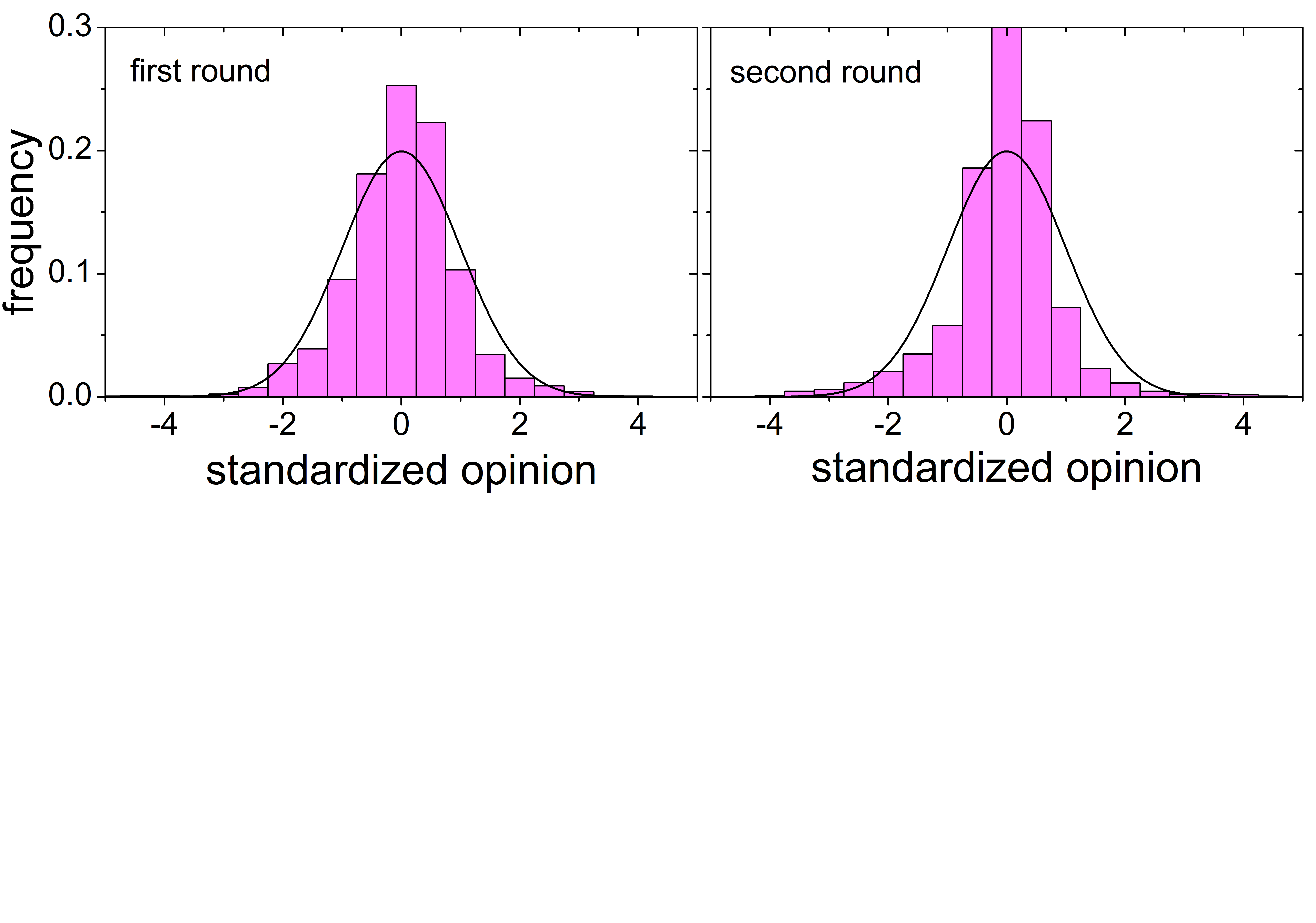

Supplement: S1 Fig — The histograms incorporate the answers to all questions in each round. The distribution of opinions for each question has been standardized by subtracting their mean value and scaling by their standard deviation. The curves correspond to a normal (Gaussian) distribution with zero mean and unitary standard deviation. (TIF) [file pone.0140406.s001.tif]
