# Supplementary material for: Opinion Formation by Social Influence: From Experiments to Modeling
Source: PLoS One. 2015 Oct 30;10(10):e0140406. doi: 10.1371/journal.pone.0140406 (PMC4627778; doi:10.1371/journal.pone.0140406)
Supplement: S1 Table — (PDF) [file pone.0140406.s002.pdf]

S1 Table. Raw data obtained from the experiment.

From left to right, columns correspond to

- question number
- subject ID
- 1st round answer
- 2nd round answer
- reference answer
- 1st round confidence
- 2nd round confidence
- reference confidence

A reference confidence of -1 is indicated for class B questions, for which no reference confidence was provided.

1	1398776357	8.000e+00	1.000e+01	1.120e+01	2	2	-1
1	1398887847	5.000e+00	8.000e+00	1.120e+01	3	3	-1
1	1398892516	1.600e+01	1.600e+01	1.120e+01	0	1	-1
1	1417185554	9.000e+00	9.000e+00	1.120e+01	4	3	-1
1	1398888387	1.500e+01	1.500e+01	1.120e+01	4	5	-1
1	1398891149	6.000e+00	8.000e+00	1.120e+01	3	3	-1
1	1398776407	1.500e+01	1.500e+01	1.120e+01	2	3	-1
1	1398891375	4.000e+00	6.000e+00	1.120e+01	3	2	-1
1	1398776634	5.000e+00	5.000e+00	1.120e+01	1	1	-1
1	1398786088	1.200e+01	1.200e+01	1.120e+01	3	3	-1
1	1398892622	7.000e+00	1.100e+01	1.120e+01	1	3	-1
1	1398888074	1.000e+01	1.000e+01	1.120e+01	1	1	-1
1	1398867272	5.000e+00	1.000e+01	1.120e+01	2	3	-1
1	1398862805	5.000e+00	1.100e+01	1.120e+01	0	4	-1
1	1398869031	7.000e+00	7.000e+00	1.120e+01	2	2	-1
1	1398889535	2.500e+01	2.000e+01	1.120e+01	0	0	-1
1	1398888718	8.000e+00	1.000e+01	1.120e+01	0	2	-1
1	1398785751	1.500e+01	1.500e+01	1.120e+01	2	2	-1
1	1398889130	1.200e+01	1.200e+01	1.120e+01	2	2	-1
1	1398891104	1.000e+01	1.000e+01	1.120e+01	3	4	-1
1	1398865276	3.000e+01	1.500e+01	1.120e+01	4	4	-1
1	1398888893	7.300e+01	1.300e+01	1.120e+01	0	0	-1
1	1398891402	3.000e+00	8.000e+00	1.120e+01	1	3	-1
1	1398888834	1.200e+01	1.200e+01	1.120e+01	1	1	-1
1	1398784937	1.000e+01	1.100e+01	1.120e+01	1	1	-1
1	1398888358	5.000e+00	5.000e+00	1.120e+01	3	3	-1
1	1398890003	4.000e+00	4.000e+00	1.120e+01	3	3	-1
1	1398891430	1.000e+01	1.000e+01	1.120e+01	0	3	-1
1	1398776404	5.000e+00	8.000e+00	1.120e+01	1	3	-1
1	1398867266	2.000e+01	1.400e+01	1.120e+01	2	3	-1
1	1398778952	3.000e+01	1.000e+01	1.120e+01	3	3	-1
1	1398865301	2.500e+01	2.500e+01	1.120e+01	2	2	-1
1	1398889925	9.000e+00	9.000e+00	1.120e+01	1	1	-1
1	1398776517	5.000e+00	1.000e+01	1.120e+01	3	4	-1
1	1408795563	1.000e+01	1.000e+01	1.120e+01	3	4	-1
1	1408809584	1.000e+01	1.100e+01	1.120e+01	1	3	-1
1	1408805301	7.000e+00	8.000e+00	1.120e+01	0	0	-1
1	1408805468	4.000e+01	1.500e+01	1.120e+01	4	2	-1
1	1408812462	1.700e+01	1.700e+01	1.120e+01	2	3	-1
1	1408808932	3.000e+01	3.000e+01	1.120e+01	3	3	-1
1	1408803130	5.000e+00	1.000e+01	1.120e+01	2	2	-1
1	1408805005	5.000e+00	1.000e+01	1.120e+01	2	3	-1
1	1408810956	1.500e+01	1.300e+01	1.120e+01	2	3	-1
1	1408815379	8.000e+00	8.000e+00	1.120e+01	1	1	-1
1	1408803877	1.000e+01	1.000e+01	1.120e+01	1	2	-1
1	1408806920	7.000e+00	7.000e+00	1.120e+01	2	2	-1
1	1408807550	1.000e+01	1.000e+01	1.120e+01	2	4	-1
1	1408804745	1.000e+01	1.000e+01	1.120e+01	1	1	-1
1	1408807503	1.000e+01	1.000e+01	1.120e+01	4	4	-1
1	1408807144	1.200e+01	1.200e+01	1.120e+01	1	3	-1
1	1408809849	2.100e+01	2.100e+01	1.120e+01	4	4	-1

1	1408803676	3.000e+01	1.500e+01	1.120e+01	1	1	-1
1	1408817241	1.500e+01	1.500e+01	1.120e+01	1	3	-1
1	1408811947	7.000e+00	9.000e+00	1.120e+01	3	3	-1
1	1408811461	1.200e+01	1.200e+01	1.120e+01	2	2	-1
1	1408814406	1.000e+01	1.100e+01	1.120e+01	2	5	-1
1	1408806191	1.500e+01	1.500e+01	1.120e+01	3	3	-1
1	1408814584	1.000e+01	1.100e+01	1.120e+01	0	3	-1
1	1408819661	3.000e+01	2.500e+01	1.120e+01	0	4	-1
1	1408812882	1.400e+01	1.200e+01	1.120e+01	3	3	-1
1	1408808711	1.200e+01	1.200e+01	1.120e+01	5	5	-1
1	1408801183	8.000e+00	8.000e+00	1.120e+01	4	4	-1
1	1408809769	1.000e+01	1.000e+01	1.120e+01	2	2	-1
1	1408799162	1.500e+01	1.500e+01	1.120e+01	4	4	-1
1	1408805091	1.200e+01	1.200e+01	1.120e+01	4	4	-1
1	1408806651	3.000e+01	1.500e+01	1.120e+01	0	1	-1
1	1408816332	1.200e+01	1.200e+01	1.120e+01	3	3	-1
1	1408809326	1.000e+01	1.000e+01	1.120e+01	2	2	-1
1	1408808791	1.000e+01	1.000e+01	1.120e+01	3	3	-1
1	1408801643	1.100e+01	1.100e+01	1.120e+01	1	3	-1
1	1408807809	7.000e+00	1.100e+01	1.120e+01	1	3	-1
1	1408797111	7.000e+00	7.000e+00	1.120e+01	3	3	-1
1	1408808623	8.000e+00	8.000e+00	1.120e+01	2	2	-1
1	1408800367	1.000e+01	1.000e+01	1.120e+01	3	4	-1
1	1408808143	1.200e+01	1.200e+01	1.120e+01	3	4	-1
1	1408802365	1.500e+01	1.000e+01	1.120e+01	1	4	-1
1	1408812007	8.000e+00	1.100e+01	1.120e+01	3	3	-1
1	1408812647	7.900e+01	5.000e+01	1.120e+01	1	1	-1
1	1408805344	1.000e+01	1.000e+01	1.120e+01	0	0	-1
1	1408816236	1.000e+01	1.000e+01	1.120e+01	3	3	-1
1	1408814110	3.000e+01	2.500e+01	1.120e+01	2	2	-1
1	1408802237	1.600e+01	1.600e+01	1.120e+01	2	4	-1
1	1408811941	1.200e+01	1.200e+01	1.120e+01	2	2	-1
1	1408820913	1.300e+01	1.300e+01	1.120e+01	2	3	-1
1	1408812832	1.200e+01	1.200e+01	1.120e+01	4	4	-1
2	1398776357	4.000e+02	4.000e+02	9.000e+02	2	2	4
2	1398887847	2.500e+02	2.500e+02	9.000e+02	1	1	4
2	1398892516	1.000e+03	1.000e+03	9.000e+02	0	1	4
2	1417185554	7.000e+02	7.000e+02	9.000e+02	3	3	4
2	1398888387	5.000e+02	5.000e+02	9.000e+02	3	3	4
2	1398891149	1.500e+03	1.000e+02	9.000e+02	2	3	4
2	1398776407	3.000e+02	3.000e+02	9.000e+02	3	3	4
2	1398891375	2.500e+03	2.500e+03	9.000e+02	3	3	4
2	1398776634	5.000e+02	9.000e+02	9.000e+02	0	1	4
2	1398786088	2.000e+03	2.000e+03	9.000e+02	3	3	4
2	1398892622	2.000e+03	1.300e+03	9.000e+02	0	1	4
2	1398888074	4.000e+02	4.000e+02	9.000e+02	1	1	4
2	1398867272	1.000e+02	2.000e+02	9.000e+02	0	1	4
2	1398862805	6.000e+02	8.000e+02	9.000e+02	0	3	4
2	1398869031	5.000e+01	5.000e+01	9.000e+02	0	0	4
2	1398889535	1.000e+02	9.000e+02	9.000e+02	1	2	4
2	1398888718	1.200e+03	1.200e+03	9.000e+02	1	1	4
2	1398785751	8.000e+02	8.000e+02	9.000e+02	3	3	4
2	1398889130	5.000e+06	5.000e+06	9.000e+02	1	1	4
2	1398891104	2.000e+02	8.000e+02	9.000e+02	0	3	4
2	1398865276	4.000e+02	9.000e+02	9.000e+02	0	3	4
2	1398888893	1.300e+01	8.000e+02	9.000e+02	0	0	4
2	1398891402	4.000e+02	9.000e+02	9.000e+02	3	3	4
2	1398888834	6.000e+02	8.000e+02	9.000e+02	2	3	4
2	1398784937	3.000e+02	9.000e+02	9.000e+02	0	1	4
2	1398888358	3.000e+02	8.000e+02	9.000e+02	0	0	4
2	1398890003	4.000e+02	7.500e+04	9.000e+02	2	4	4
2	1398891430	4.000e+02	5.000e+02	9.000e+02	3	3	4
2	1398776404	2.000e+02	2.500e+02	9.000e+02	2	3	4
2	1398867266	3.000e+03	1.000e+03	9.000e+02	2	4	4
2	1398778952	2.000e+02	2.000e+02	9.000e+02	1	1	4
2	1398865301	2.300e+03	1.500e+03	9.000e+02	2	3	4
2	1398889925	9.500e+02	9.500e+02	9.000e+02	3	3	4
2	1398776517	5.000e+01	1.000e+03	9.000e+02	0	0	4
2	1408795563	1.900e+03	1.000e+02	9.000e+02	1	3	4

2	1408809584	6.000e+02	7.000e+02	9.000e+02	4	4	4
2	1408805301	1.200e+03	1.200e+03	9.000e+02	2	2	4
2	1408805468	1.000e+03	1.000e+03	9.000e+02	5	5	4
2	1408812462	8.000e+02	8.000e+02	9.000e+02	3	3	4
2	1408808932	5.000e+01	1.000e+02	9.000e+02	0	2	4
2	1408803130	1.300e+03	1.000e+03	9.000e+02	3	3	4
2	1408805005	7.000e+01	1.500e+02	9.000e+02	0	3	4
2	1408810956	7.500e+02	7.500e+02	9.000e+02	2	3	4
2	1408815379	7.000e+02	7.000e+02	9.000e+02	3	4	4
2	1408803877	6.000e+02	9.000e+02	9.000e+02	1	4	4
2	1408806920	2.000e+03	1.200e+03	9.000e+02	1	1	4
2	1408807550	2.000e+03	1.500e+03	9.000e+02	3	4	4
2	1408804745	3.000e+02	3.000e+02	9.000e+02	1	1	4
2	1408807503	9.000e+02	9.000e+02	9.000e+02	3	3	4
2	1408807144	7.000e+02	8.000e+02	9.000e+02	4	4	4
2	1408809849	2.500e+03	1.500e+03	9.000e+02	3	3	4
2	1408803676	1.300e+03	1.000e+03	9.000e+02	3	3	4
2	1408817241	2.000e+02	9.000e+02	9.000e+02	0	3	4
2	1408811947	7.000e+02	8.000e+02	9.000e+02	3	3	4
2	1408811461	2.000e+03	2.000e+03	9.000e+02	3	3	4
2	1408814406	7.000e+02	7.000e+02	9.000e+02	4	4	4
2	1408806191	2.000e+03	1.000e+03	9.000e+02	3	3	4
2	1408814584	1.500e+03	9.000e+02	9.000e+02	0	3	4
2	1408819661	4.000e+02	9.000e+02	9.000e+02	0	0	4
2	1408812882	9.000e+02	9.000e+02	9.000e+02	3	3	4
2	1408808711	1.600e+03	1.600e+03	9.000e+02	3	3	4
2	1408801183	1.000e+03	1.000e+03	9.000e+02	4	4	4
2	1408809769	3.000e+02	9.000e+02	9.000e+02	0	2	4
2	1408799162	1.500e+03	1.500e+03	9.000e+02	3	3	4
2	1408805091	1.000e+03	1.000e+03	9.000e+02	3	3	4
2	1408806651	7.500e+02	7.500e+02	9.000e+02	4	4	4
2	1408816332	8.000e+02	8.000e+02	9.000e+02	4	4	4
2	1408809326	9.000e+02	9.000e+02	9.000e+02	2	3	4
2	1408808791	6.000e+02	7.000e+02	9.000e+02	4	4	4
2	1408801643	9.000e+02	9.000e+02	9.000e+02	3	3	4
2	1408807809	1.200e+03	1.000e+03	9.000e+02	2	2	4
2	1408797111	8.000e+02	8.000e+02	9.000e+02	3	3	4
2	1408808623	7.500e+02	7.000e+02	9.000e+02	4	4	4
2	1408800367	9.000e+02	9.000e+02	9.000e+02	4	4	4
2	1408808143	1.000e+02	1.000e+02	9.000e+02	0	0	4
2	1408802365	9.000e+02	9.000e+02	9.000e+02	4	5	4
2	1408812007	2.500e+03	2.500e+03	9.000e+02	3	3	4
2	1408812647	9.000e+02	9.000e+02	9.000e+02	2	4	4
2	1408805344	9.000e+02	9.000e+02	9.000e+02	4	4	4
2	1408816236	8.000e+02	8.500e+02	9.000e+02	3	4	4
2	1408814110	5.000e+02	9.000e+02	9.000e+02	0	0	4
2	1408802237	7.500e+02	7.500e+02	9.000e+02	4	4	4
2	1408811941	9.000e+02	9.000e+02	9.000e+02	2	2	4
2	1408820913	1.400e+04	1.400e+04	9.000e+02	2	2	4
2	1408812832	7.000e+02	7.000e+02	9.000e+02	4	5	4
3	1398776357	9.000e+01	9.000e+01	7.520e+01	3	3	-1
3	1398887847	4.000e+01	4.000e+01	7.520e+01	4	4	-1
3	1398892516	8.000e+01	8.000e+01	7.520e+01	2	3	-1
3	1417185554	4.500e+01	4.500e+01	7.520e+01	3	2	-1
3	1398888387	1.000e+00	7.000e+01	7.520e+01	4	4	-1
3	1398891149	1.200e+02	9.000e+01	7.520e+01	3	3	-1
3	1398776407	1.000e+02	1.000e+02	7.520e+01	3	3	-1
3	1398891375	1.200e+02	1.000e+02	7.520e+01	3	3	-1
3	1398776634	7.000e+01	7.000e+01	7.520e+01	2	2	-1
3	1398786088	5.000e+01	7.000e+01	7.520e+01	3	3	-1
3	1398892622	2.500e+00	2.000e+00	7.520e+01	3	3	-1
3	1398888074	2.000e+00	1.000e+00	7.520e+01	1	1	-1
3	1398867272	6.000e+01	7.000e+01	7.520e+01	3	3	-1
3	1398862805	1.000e+02	1.000e+02	7.520e+01	3	3	-1
3	1398869031	1.000e+02	8.000e+01	7.520e+01	3	4	-1
3	1398889535	1.200e+02	1.000e+02	7.520e+01	1	1	-1
3	1398888718	1.000e+00	8.000e+01	7.520e+01	2	3	-1
3	1398785751	6.000e+01	6.000e+01	7.520e+01	3	3	-1
3	1398889130	1.000e+02	1.000e+02	7.520e+01	3	3	-1

3	1398891104	1.000e+00	1.000e+00	7.520e+01	5	5	-1
3	1398865276	2.000e+01	7.000e+01	7.520e+01	4	4	-1
3	1398888893	1.300e+00	7.000e+01	7.520e+01	0	0	-1
3	1398891402	1.150e+00	1.150e+00	7.520e+01	3	3	-1
3	1398888834	1.000e+02	8.000e+01	7.520e+01	1	1	-1
3	1398784937	1.000e+02	1.000e+02	7.520e+01	3	3	-1
3	1398888358	8.000e+01	8.000e+01	7.520e+01	3	3	-1
3	1398890003	1.200e+02	1.200e+02	7.520e+01	4	4	-1
3	1398891430	1.800e+00	1.000e+02	7.520e+01	4	4	-1
3	1398776404	2.000e+03	1.500e+03	7.520e+01	3	3	-1
3	1398867266	1.200e+02	8.000e+01	7.520e+01	3	4	-1
3	1398778952	9.000e+01	7.500e+01	7.520e+01	4	4	-1
3	1398865301	1.000e+00	8.000e+01	7.520e+01	2	2	-1
3	1398889925	8.000e+01	8.000e+01	7.520e+01	4	4	-1
3	1398776517	8.000e+01	8.000e+01	7.520e+01	3	3	-1
3	1408795563	1.200e+02	1.200e+02	7.520e+01	3	3	-1
3	1408809584	7.500e+01	7.500e+01	7.520e+01	4	4	-1
3	1408805301	1.400e+02	1.400e+02	7.520e+01	3	1	-1
3	1408805468	7.000e+01	7.000e+01	7.520e+01	3	4	-1
3	1408812462	5.000e+01	7.000e+01	7.520e+01	3	3	-1
3	1408808932	1.000e+02	1.000e+02	7.520e+01	3	3	-1
3	1408803130	1.000e+00	1.000e+00	7.520e+01	3	3	-1
3	1408805005	1.400e+00	1.050e+02	7.520e+01	1	2	-1
3	1408810956	1.250e+02	1.000e+02	7.520e+01	3	3	-1
3	1408815379	1.200e+02	1.000e+02	7.520e+01	3	3	-1
3	1408803877	1.200e+02	1.000e+02	7.520e+01	2	2	-1
3	1408806920	1.000e+02	1.000e+02	7.520e+01	3	3	-1
3	1408807550	8.000e+01	8.000e+01	7.520e+01	3	4	-1
3	1408804745	8.000e+01	8.000e+01	7.520e+01	3	3	-1
3	1408807503	1.000e+02	1.000e+02	7.520e+01	4	4	-1
3	1408807144	9.000e+01	9.000e+01	7.520e+01	4	4	-1
3	1408809849	1.000e+02	8.500e+01	7.520e+01	4	3	-1
3	1408803676	1.200e+02	1.100e+02	7.520e+01	3	3	-1
3	1408817241	1.000e+02	1.000e+02	7.520e+01	2	3	-1
3	1408811947	8.500e+01	8.000e+01	7.520e+01	3	3	-1
3	1408811461	8.500e+01	8.000e+01	7.520e+01	3	3	-1
3	1408814406	2.500e+02	7.500e+01	7.520e+01	2	5	-1
3	1408806191	1.000e+02	1.000e+02	7.520e+01	4	4	-1
3	1408814584	1.000e+02	8.000e+01	7.520e+01	3	3	-1
3	1408819661	8.000e+01	8.000e+01	7.520e+01	4	4	-1
3	1408812882	7.000e+01	7.000e+01	7.520e+01	3	4	-1
3	1408808711	5.000e+01	5.000e+01	7.520e+01	4	4	-1
3	1408801183	1.000e+02	7.500e+01	7.520e+01	4	4	-1
3	1408809769	1.000e+02	1.000e+02	7.520e+01	3	3	-1
3	1408799162	1.100e+02	1.100e+02	7.520e+01	4	4	-1
3	1408805091	1.300e+00	1.300e+02	7.520e+01	4	4	-1
3	1408806651	9.000e+01	7.500e+01	7.520e+01	4	4	-1
3	1408816332	1.000e+02	7.500e+01	7.520e+01	4	4	-1
3	1408809326	1.200e+02	1.200e+02	7.520e+01	3	3	-1
3	1408808791	1.200e+02	1.000e+02	7.520e+01	4	3	-1
3	1408801643	1.000e+02	1.000e+02	7.520e+01	4	3	-1
3	1408807809	9.000e+01	8.000e+01	7.520e+01	4	3	-1
3	1408797111	9.000e+01	9.000e+01	7.520e+01	4	4	-1
3	1408808623	6.500e+01	6.500e+01	7.520e+01	3	3	-1
3	1408800367	1.200e+02	1.000e+02	7.520e+01	3	3	-1
3	1408808143	1.000e+00	1.000e+00	7.520e+01	4	3	-1
3	1408802365	9.000e+01	7.500e+01	7.520e+01	4	4	-1
3	1408812007	1.300e+02	1.300e+02	7.520e+01	4	5	-1
3	1408812647	1.300e+02	1.300e+02	7.520e+01	3	3	-1
3	1408805344	1.000e+02	1.000e+02	7.520e+01	3	3	-1
3	1408816236	6.000e+01	7.000e+01	7.520e+01	3	3	-1
3	1408814110	9.000e+01	8.000e+01	7.520e+01	4	4	-1
3	1408802237	5.000e+01	7.000e+01	7.520e+01	3	4	-1
3	1408811941	7.000e+01	7.000e+01	7.520e+01	3	3	-1
3	1408820913	1.000e+02	8.000e+01	7.520e+01	3	3	-1
3	1408812832	9.000e+01	8.000e+01	7.520e+01	4	4	-1
4	1398776357	1.000e+02	1.000e+02	7.000e+01	4	4	3
4	1398887847	1.000e+02	1.000e+02	7.000e+01	4	2	3
4	1398892516	2.000e+02	1.000e+02	7.000e+01	2	2	3

4	1417185554	6.000e+00	6.000e+00	7.000e+01	1	1	3
4	1398888387	3.300e+01	3.300e+01	7.000e+01	3	3	3
4	1398891149	2.000e+01	2.000e+01	7.000e+01	3	3	3
4	1398776407	2.000e+02	1.500e+02	7.000e+01	3	3	3
4	1398891375	1.000e+03	1.500e+02	7.000e+01	1	2	3
4	1398776634	5.000e+00	6.000e+01	7.000e+01	2	3	3
4	1398786088	1.000e+03	1.000e+03	7.000e+01	3	3	3
4	1398892622	3.300e+01	3.300e+01	7.000e+01	2	4	3
4	1398888074	5.000e+01	5.000e+01	7.000e+01	3	3	3
4	1398867272	1.500e+01	5.000e+01	7.000e+01	3	3	3
4	1398862805	2.500e+01	2.500e+01	7.000e+01	3	3	3
4	1398869031	6.000e+01	6.000e+01	7.000e+01	4	4	3
4	1398889535	1.000e+02	1.000e+02	7.000e+01	1	1	3
4	1398888718	1.000e+02	1.000e+02	7.000e+01	2	2	3
4	1398785751	5.000e+01	5.000e+01	7.000e+01	3	3	3
4	1398889130	1.000e+01	1.000e+02	7.000e+01	2	2	3
4	1398891104	1.000e+02	5.000e+01	7.000e+01	4	4	3
4	1398865276	3.000e+01	3.000e+01	7.000e+01	3	3	3
4	1398888893	1.000e+02	1.000e+02	7.000e+01	2	2	3
4	1398891402	3.500e+01	5.000e+01	7.000e+01	2	3	3
4	1398888834	5.000e+01	5.000e+01	7.000e+01	0	2	3
4	1398784937	1.000e+02	1.000e+02	7.000e+01	3	3	3
4	1398888358	3.000e+01	3.000e+01	7.000e+01	3	3	3
4	1398890003	3.000e+00	5.000e+01	7.000e+01	2	3	3
4	1398891430	5.000e+03	5.000e+03	7.000e+01	5	5	3
4	1398776404	4.000e+01	4.000e+01	7.000e+01	4	4	3
4	1398867266	6.000e+00	4.000e+01	7.000e+01	3	3	3
4	1398778952	6.000e+01	6.000e+01	7.000e+01	4	4	3
4	1398865301	5.000e+02	5.000e+02	7.000e+01	2	2	3
4	1398889925	8.000e+01	8.000e+01	7.000e+01	3	3	3
4	1398776517	1.000e+01	6.000e+01	7.000e+01	3	2	3
4	1408795563	5.000e+01	5.000e+01	7.000e+01	4	4	3
4	1408809584	5.000e+01	5.000e+01	7.000e+01	3	3	3
4	1408805301	5.000e+01	5.000e+01	7.000e+01	2	2	3
4	1408805468	4.000e+01	4.000e+01	7.000e+01	3	3	3
4	1408812462	5.000e+01	5.000e+01	7.000e+01	3	4	3
4	1408808932	3.000e+02	2.000e+02	7.000e+01	3	3	3
4	1408803130	3.000e+01	3.000e+01	7.000e+01	4	4	3
4	1408805005	5.000e+00	5.000e+01	7.000e+01	1	2	3
4	1408810956	1.000e+02	1.000e+02	7.000e+01	3	3	3
4	1408815379	3.000e+02	3.000e+02	7.000e+01	2	2	3
4	1408803877	5.000e+01	5.000e+01	7.000e+01	3	3	3
4	1408806920	1.000e+02	1.000e+02	7.000e+01	3	3	3
4	1408807550	3.000e+00	3.000e+00	7.000e+01	3	3	3
4	1408804745	2.000e+02	2.000e+02	7.000e+01	3	3	3
4	1408807503	5.000e+00	1.000e+01	7.000e+01	3	2	3
4	1408807144	3.000e+02	3.000e+02	7.000e+01	3	3	3
4	1408809849	2.000e+02	2.000e+02	7.000e+01	3	3	3
4	1408803676	1.000e+02	1.000e+02	7.000e+01	2	2	3
4	1408817241	1.000e+01	1.000e+01	7.000e+01	4	4	3
4	1408811947	2.000e+02	2.000e+02	7.000e+01	1	1	3
4	1408811461	2.000e+01	3.000e+01	7.000e+01	4	4	3
4	1408814406	1.000e+01	1.000e+01	7.000e+01	3	3	3
4	1408806191	5.000e+00	5.000e+00	7.000e+01	1	1	3
4	1408814584	5.000e+02	1.500e+02	7.000e+01	0	1	3
4	1408819661	1.000e+00	1.000e+00	7.000e+01	4	4	3
4	1408812882	5.000e+01	5.000e+01	7.000e+01	4	4	3
4	1408808711	4.000e+01	6.000e+01	7.000e+01	4	3	3
4	1408801183	8.000e+01	8.000e+01	7.000e+01	4	4	3
4	1408809769	1.000e+02	1.000e+02	7.000e+01	2	2	3
4	1408799162	3.000e+01	3.000e+01	7.000e+01	4	4	3
4	1408805091	3.000e+01	3.000e+01	7.000e+01	4	4	3
4	1408806651	2.000e+02	1.500e+02	7.000e+01	3	3	3
4	1408816332	2.000e+01	2.000e+01	7.000e+01	3	3	3
4	1408809326	1.000e+02	1.000e+02	7.000e+01	3	3	3
4	1408808791	2.000e+01	2.000e+01	7.000e+01	4	4	3
4	1408801643	5.000e+01	5.000e+01	7.000e+01	1	2	3
4	1408807809	7.000e+01	7.000e+01	7.000e+01	2	2	3
4	1408797111	1.000e+02	1.000e+02	7.000e+01	3	3	3

4	1408808623	3.000e+01	3.000e+01	7.000e+01	3	3	3
4	1408800367	3.000e+02	1.000e+02	7.000e+01	2	3	3
4	1408808143	2.000e+01	3.000e+01	7.000e+01	4	3	3
4	1408802365	4.000e+01	6.000e+01	7.000e+01	3	3	3
4	1408812007	1.000e+02	1.000e+02	7.000e+01	3	3	3
4	1408812647	2.700e+01	2.700e+01	7.000e+01	4	4	3
4	1408805344	2.500e+01	2.500e+01	7.000e+01	4	4	3
4	1408816236	3.000e+01	5.000e+01	7.000e+01	2	3	3
4	1408814110	5.000e+01	5.500e+01	7.000e+01	2	2	3
4	1408802237	5.000e+00	5.000e+01	7.000e+01	3	3	3
4	1408811941	4.000e+00	4.000e+00	7.000e+01	3	3	3
4	1408820913	5.000e+01	5.000e+01	7.000e+01	3	3	3
4	1408812832	8.000e+01	8.000e+01	7.000e+01	3	4	3
5	1398776357	3.000e+01	3.000e+01	2.060e+01	3	3	-1
5	1398887847	3.000e+01	3.000e+01	2.060e+01	4	4	-1
5	1398892516	3.000e+01	3.000e+01	2.060e+01	2	3	-1
5	1417185554	1.000e+01	1.000e+01	2.060e+01	3	0	-1
5	1398888387	2.500e+01	2.500e+01	2.060e+01	3	3	-1
5	1398891149	1.000e+01	1.000e+01	2.060e+01	3	3	-1
5	1398776407	2.000e+01	2.000e+01	2.060e+01	4	5	-1
5	1398891375	2.500e+01	2.500e+01	2.060e+01	3	3	-1
5	1398776634	2.000e+01	2.000e+01	2.060e+01	2	2	-1
5	1398786088	4.500e+01	4.500e+01	2.060e+01	4	4	-1
5	1398892622	2.000e+01	2.000e+01	2.060e+01	4	5	-1
5	1398888074	3.000e+01	3.000e+01	2.060e+01	3	3	-1
5	1398867272	3.000e+01	3.000e+01	2.060e+01	3	3	-1
5	1398862805	1.500e+01	1.500e+01	2.060e+01	2	2	-1
5	1398869031	2.500e+01	2.500e+01	2.060e+01	3	3	-1
5	1398889535	2.500e+01	2.500e+01	2.060e+01	1	1	-1
5	1398888718	1.200e+01	2.400e+01	2.060e+01	2	2	-1
5	1398785751	2.000e+01	2.000e+01	2.060e+01	2	2	-1
5	1398889130	2.400e+01	2.400e+01	2.060e+01	2	2	-1
5	1398891104	2.500e+01	2.500e+01	2.060e+01	4	5	-1
5	1398865276	2.000e+01	2.000e+01	2.060e+01	4	4	-1
5	1398888893	1.600e+01	1.600e+01	2.060e+01	5	5	-1
5	1398891402	2.100e+01	2.100e+01	2.060e+01	3	4	-1
5	1398888834	4.000e+01	3.000e+01	2.060e+01	1	2	-1
5	1398784937	3.000e+01	3.000e+01	2.060e+01	0	1	-1
5	1398888358	3.000e+01	2.500e+01	2.060e+01	2	2	-1
5	1398890003	5.000e+01	5.000e+01	2.060e+01	3	3	-1
5	1398891430	2.000e+01	2.000e+01	2.060e+01	4	5	-1
5	1398776404	2.000e+01	2.000e+01	2.060e+01	5	5	-1
5	1398867266	2.400e+01	2.400e+01	2.060e+01	4	4	-1
5	1398778952	1.800e+01	1.800e+01	2.060e+01	4	4	-1
5	1398865301	2.500e+01	2.500e+01	2.060e+01	3	3	-1
5	1398889925	2.000e+01	2.000e+01	2.060e+01	4	4	-1
5	1398776517	3.000e+01	3.000e+01	2.060e+01	3	3	-1
5	1408795563	4.000e+01	3.000e+01	2.060e+01	3	4	-1
5	1408809584	2.000e+01	2.000e+01	2.060e+01	2	3	-1
5	1408805301	3.500e+01	3.500e+01	2.060e+01	2	5	-1
5	1408805468	2.500e+01	2.500e+01	2.060e+01	4	5	-1
5	1408812462	4.000e+01	4.000e+01	2.060e+01	3	3	-1
5	1408808932	2.500e+01	2.500e+01	2.060e+01	3	3	-1
5	1408803130	1.200e+01	1.200e+01	2.060e+01	3	3	-1
5	1408805005	2.000e+01	2.000e+01	2.060e+01	4	4	-1
5	1408810956	1.400e+01	1.800e+01	2.060e+01	4	4	-1
5	1408815379	2.000e+01	2.000e+01	2.060e+01	3	4	-1
5	1408803877	3.000e+01	3.000e+01	2.060e+01	2	2	-1
5	1408806920	2.000e+01	2.000e+01	2.060e+01	2	2	-1
5	1408807550	4.000e+01	3.000e+01	2.060e+01	3	4	-1
5	1408804745	2.000e+01	2.000e+01	2.060e+01	3	3	-1
5	1408807503	3.500e+01	3.500e+01	2.060e+01	2	2	-1
5	1408807144	6.500e+01	4.500e+01	2.060e+01	3	4	-1
5	1408809849	3.000e+01	3.000e+01	2.060e+01	4	4	-1
5	1408803676	2.000e+01	2.000e+01	2.060e+01	3	4	-1
5	1408817241	3.000e+01	3.000e+01	2.060e+01	3	3	-1
5	1408811947	2.500e+01	2.500e+01	2.060e+01	3	3	-1
5	1408811461	2.800e+01	2.800e+01	2.060e+01	3	3	-1
5	1408814406	3.000e+01	2.100e+01	2.060e+01	3	5	-1

5	1408806191	3.000e+01	3.000e+01	2.060e+01	4	4	-1
5	1408814584	3.000e+01	2.500e+01	2.060e+01	3	3	-1
5	1408819661	2.400e+01	2.400e+01	2.060e+01	4	4	-1
5	1408812882	2.800e+01	2.600e+01	2.060e+01	3	3	-1
5	1408808711	4.400e+01	4.400e+01	2.060e+01	3	3	-1
5	1408801183	3.000e+01	3.000e+01	2.060e+01	0	4	-1
5	1408809769	4.000e+01	4.000e+01	2.060e+01	3	3	-1
5	1408799162	2.000e+01	2.000e+01	2.060e+01	3	3	-1
5	1408805091	2.000e+01	2.000e+01	2.060e+01	3	3	-1
5	1408806651	2.500e+01	2.100e+01	2.060e+01	3	4	-1
5	1408816332	5.000e+01	2.000e+01	2.060e+01	4	4	-1
5	1408809326	4.000e+01	4.000e+01	2.060e+01	3	3	-1
5	1408808791	3.000e+01	2.500e+01	2.060e+01	4	4	-1
5	1408801643	2.000e+01	2.000e+01	2.060e+01	3	4	-1
5	1408807809	2.000e+01	2.000e+01	2.060e+01	4	5	-1
5	1408797111	3.000e+01	3.000e+01	2.060e+01	3	3	-1
5	1408808623	2.600e+01	2.600e+01	2.060e+01	3	3	-1
5	1408800367	5.000e+01	4.000e+01	2.060e+01	2	3	-1
5	1408808143	1.500e+01	2.000e+01	2.060e+01	3	4	-1
5	1408802365	1.500e+01	2.000e+01	2.060e+01	2	4	-1
5	1408812007	2.000e+01	2.000e+01	2.060e+01	3	5	-1
5	1408812647	2.000e+01	2.000e+01	2.060e+01	3	4	-1
5	1408805344	3.500e+01	3.500e+01	2.060e+01	3	3	-1
5	1408816236	3.000e+01	2.500e+01	2.060e+01	3	3	-1
5	1408814110	2.500e+01	2.500e+01	2.060e+01	3	3	-1
5	1408802237	2.000e+01	2.000e+01	2.060e+01	2	4	-1
5	1408811941	6.000e+01	6.000e+01	2.060e+01	3	3	-1
5	1408820913	1.500e+01	1.500e+01	2.060e+01	4	4	-1
5	1408812832	2.500e+01	2.000e+01	2.060e+01	4	4	-1
6	1398776357	1.000e+00	1.000e+00	1.200e+00	4	4	0
6	1398887847	3.000e+00	3.000e+00	1.200e+00	0	0	0
6	1398892516	7.500e-01	7.500e-01	1.200e+00	3	3	0
6	1417185554	7.000e-01	7.000e-01	1.200e+00	3	3	0
6	1398888387	5.000e-01	1.000e+00	1.200e+00	0	3	0
6	1398891149	5.000e-01	5.000e-01	1.200e+00	3	3	0
6	1398776407	1.500e+00	1.500e+00	1.200e+00	4	5	0
6	1398891375	1.500e+00	1.500e+00	1.200e+00	4	4	0
6	1398776634	1.000e+00	1.000e+00	1.200e+00	1	2	0
6	1398786088	1.500e+00	1.500e+00	1.200e+00	3	3	0
6	1398892622	1.500e+00	1.500e+00	1.200e+00	4	4	0
6	1398888074	1.000e+00	1.000e+00	1.200e+00	3	3	0
6	1398867272	5.000e-01	5.000e-01	1.200e+00	3	3	0
6	1398862805	7.000e-01	7.000e-01	1.200e+00	3	3	0
6	1398869031	1.200e+00	1.200e+00	1.200e+00	3	3	0
6	1398889535	2.000e+00	1.000e+00	1.200e+00	1	1	0
6	1398888718	3.000e+00	2.000e+00	1.200e+00	0	1	0
6	1398785751	1.500e+00	1.500e+00	1.200e+00	3	3	0
6	1398889130	5.000e+00	1.200e+00	1.200e+00	1	4	0
6	1398891104	2.000e+00	1.500e+00	1.200e+00	4	4	0
6	1398865276	1.000e+00	1.000e+00	1.200e+00	3	3	0
6	1398888893	2.000e+00	2.000e+00	1.200e+00	3	3	0
6	1398891402	1.500e+00	1.500e+00	1.200e+00	5	5	0
6	1398888834	1.500e+00	1.300e+00	1.200e+00	2	2	0
6	1398784937	1.000e+00	1.000e+00	1.200e+00	2	2	0
6	1398888358	1.000e+00	1.000e+00	1.200e+00	3	3	0
6	1398890003	1.500e+00	1.500e+00	1.200e+00	3	3	0
6	1398891430	1.500e+00	1.500e+00	1.200e+00	5	5	0
6	1398776404	1.000e+00	1.000e+00	1.200e+00	4	4	0
6	1398867266	5.000e-01	1.000e+00	1.200e+00	2	3	0
6	1398778952	1.000e+00	1.000e+00	1.200e+00	3	3	0
6	1398865301	8.500e+02	8.500e+02	1.200e+00	2	2	0
6	1398889925	5.000e-01	5.000e-01	1.200e+00	4	4	0
6	1398776517	7.000e-01	2.000e+00	1.200e+00	3	3	0
6	1408795563	6.500e-01	6.500e-01	1.200e+00	4	4	0
6	1408809584	2.000e+00	1.600e+00	1.200e+00	4	3	0
6	1408805301	1.200e+00	1.200e+00	1.200e+00	3	5	0
6	1408805468	1.000e+00	1.000e+00	1.200e+00	4	4	0
6	1408812462	1.000e+00	1.000e+00	1.200e+00	2	2	0
6	1408808932	2.000e+00	2.000e+00	1.200e+00	3	3	0

6	1408803130	1.250e+00	1.250e+00	1.200e+00	2	2	0
6	1408805005	5.000e-01	5.000e-01	1.200e+00	5	5	0
6	1408810956	7.500e-01	7.500e-01	1.200e+00	3	3	0
6	1408815379	1.000e+00	1.000e+00	1.200e+00	2	2	0
6	1408803877	2.000e+00	2.000e+00	1.200e+00	2	1	0
6	1408806920	5.000e-01	5.000e-01	1.200e+00	5	5	0
6	1408807550	1.000e+00	1.000e+00	1.200e+00	2	2	0
6	1408804745	2.000e+00	2.000e+00	1.200e+00	3	3	0
6	1408807503	3.500e+00	3.500e+00	1.200e+00	3	3	0
6	1408807144	2.000e+00	2.000e+00	1.200e+00	3	3	0
6	1408809849	3.000e+00	3.000e+00	1.200e+00	3	3	0
6	1408803676	5.000e-01	5.000e-01	1.200e+00	5	5	0
6	1408817241	4.000e+00	4.000e+00	1.200e+00	3	3	0
6	1408811947	2.000e+00	1.500e+00	1.200e+00	3	3	0
6	1408811461	2.700e+00	2.700e+00	1.200e+00	3	3	0
6	1408814406	3.000e+00	3.000e+00	1.200e+00	2	2	0
6	1408806191	1.000e+00	1.000e+00	1.200e+00	4	4	0
6	1408814584	1.000e+00	1.000e+00	1.200e+00	2	2	0
6	1408819661	5.000e-01	5.000e-01	1.200e+00	3	3	0
6	1408812882	1.500e+00	1.500e+00	1.200e+00	4	4	0
6	1408808711	3.000e+00	3.000e+00	1.200e+00	3	3	0
6	1408801183	7.500e-01	7.500e-01	1.200e+00	4	4	0
6	1408809769	5.000e-01	5.000e-01	1.200e+00	3	3	0
6	1408799162	8.000e-01	8.000e-01	1.200e+00	3	3	0
6	1408805091	1.000e+00	1.000e+00	1.200e+00	3	3	0
6	1408806651	2.000e+00	2.000e+00	1.200e+00	3	3	0
6	1408816332	1.000e+00	1.000e+00	1.200e+00	2	2	0
6	1408809326	7.500e-01	7.500e-01	1.200e+00	3	3	0
6	1408808791	1.000e+00	1.000e+00	1.200e+00	4	4	0
6	1408801643	1.000e+00	1.000e+00	1.200e+00	2	2	0
6	1408807809	1.500e+00	1.500e+00	1.200e+00	3	3	0
6	1408797111	1.000e+00	1.000e+00	1.200e+00	3	3	0
6	1408808623	1.000e+00	1.000e+00	1.200e+00	3	3	0
6	1408800367	5.000e-01	6.000e-01	1.200e+00	3	3	0
6	1408808143	1.500e+00	1.500e+00	1.200e+00	4	4	0
6	1408802365	2.000e+00	2.000e+00	1.200e+00	1	1	0
6	1408812007	1.000e+00	1.000e+00	1.200e+00	3	3	0
6	1408812647	1.000e+00	1.100e+00	1.200e+00	3	4	0
6	1408805344	1.500e+00	1.500e+00	1.200e+00	3	3	0
6	1408816236	4.500e-01	4.500e-01	1.200e+00	3	3	0
6	1408814110	1.500e+00	1.500e+00	1.200e+00	2	2	0
6	1408802237	4.000e-01	4.000e-01	1.200e+00	3	3	0
6	1408811941	5.000e+00	3.000e+00	1.200e+00	2	2	0
6	1408820913	5.000e-01	5.000e-01	1.200e+00	0	0	0
6	1408812832	2.000e+00	2.000e+00	1.200e+00	4	4	0
7	1398776357	5.000e+00	5.000e+00	1.250e+01	3	3	-1
7	1398887847	3.000e+00	3.000e+00	1.250e+01	2	2	-1
7	1398892516	8.000e+00	1.000e+01	1.250e+01	3	3	-1
7	1417185554	2.000e+02	2.000e+02	1.250e+01	3	3	-1
7	1398888387	1.000e-02	1.000e-02	1.250e+01	1	1	-1
7	1398891149	2.000e+01	2.000e+01	1.250e+01	3	3	-1
7	1398776407	7.000e+00	7.000e+00	1.250e+01	3	3	-1
7	1398891375	3.000e+00	8.000e+00	1.250e+01	2	2	-1
7	1398776634	1.000e+01	1.000e+01	1.250e+01	0	2	-1
7	1398786088	5.000e+00	5.000e+00	1.250e+01	3	3	-1
7	1398892622	1.100e+01	1.100e+01	1.250e+01	4	5	-1
7	1398888074	5.000e+01	5.000e+01	1.250e+01	3	3	-1
7	1398867272	5.000e+00	1.000e+01	1.250e+01	2	3	-1
7	1398862805	1.000e+00	1.000e+00	1.250e+01	2	2	-1
7	1398869031	2.000e+01	2.000e+01	1.250e+01	3	3	-1
7	1398889535	2.000e+01	2.000e+01	1.250e+01	0	0	-1
7	1398888718	2.000e+01	2.000e+01	1.250e+01	0	2	-1
7	1398785751	5.000e+00	7.000e+00	1.250e+01	4	4	-1
7	1398889130	2.000e+00	1.200e+01	1.250e+01	1	4	-1
7	1398891104	1.000e+01	1.000e+01	1.250e+01	4	4	-1
7	1398865276	0.000e+00	1.000e+01	1.250e+01	3	3	-1
7	1398888893	1.750e+02	5.000e+01	1.250e+01	3	3	-1
7	1398891402	4.000e+00	7.000e+00	1.250e+01	0	3	-1
7	1398888834	5.000e+01	1.000e+01	1.250e+01	1	3	-1

7	1398784937	5.000e+01	5.000e+01	1.250e+01	0	0	-1
7	1398888358	5.000e+01	1.500e+01	1.250e+01	3	3	-1
7	1398890003	5.000e+01	5.000e+01	1.250e+01	3	4	-1
7	1398891430	5.000e+01	2.500e+01	1.250e+01	4	4	-1
7	1398776404	2.800e+01	1.500e+01	1.250e+01	2	4	-1
7	1398867266	5.000e-01	2.000e+01	1.250e+01	2	3	-1
7	1398778952	1.000e+00	1.000e+00	1.250e+01	5	5	-1
7	1398865301	1.500e+01	1.500e+01	1.250e+01	2	2	-1
7	1398889925	4.000e+00	4.000e+00	1.250e+01	3	3	-1
7	1398776517	4.000e+02	1.000e+01	1.250e+01	3	3	-1
7	1408795563	6.000e+01	2.000e+01	1.250e+01	2	4	-1
7	1408809584	4.000e+00	5.000e+00	1.250e+01	3	4	-1
7	1408805301	2.000e+01	2.000e+01	1.250e+01	2	2	-1
7	1408805468	2.000e+01	1.500e+01	1.250e+01	3	4	-1
7	1408812462	3.000e+00	3.000e+00	1.250e+01	2	1	-1
7	1408808932	5.000e+00	1.000e+01	1.250e+01	2	2	-1
7	1408803130	2.500e+01	2.500e+01	1.250e+01	1	1	-1
7	1408805005	2.900e+00	2.900e+00	1.250e+01	3	3	-1
7	1408810956	5.000e+00	7.500e+00	1.250e+01	3	3	-1
7	1408815379	2.000e+01	2.000e+01	1.250e+01	2	3	-1
7	1408803877	1.000e+01	1.000e+01	1.250e+01	2	3	-1
7	1408806920	1.000e+02	3.000e+01	1.250e+01	3	3	-1
7	1408807550	5.000e+01	3.000e+01	1.250e+01	3	4	-1
7	1408804745	5.000e+01	5.000e+01	1.250e+01	1	1	-1
7	1408807503	1.000e+01	1.000e+01	1.250e+01	2	2	-1
7	1408807144	4.000e+00	8.000e+00	1.250e+01	3	3	-1
7	1408809849	5.000e+01	3.000e+01	1.250e+01	3	3	-1
7	1408803676	3.000e+01	2.000e+01	1.250e+01	1	1	-1
7	1408817241	5.000e-01	1.000e+00	1.250e+01	2	2	-1
7	1408811947	2.000e+01	1.000e+01	1.250e+01	1	1	-1
7	1408811461	3.000e+00	8.000e+00	1.250e+01	3	3	-1
7	1408814406	5.000e+01	1.300e+01	1.250e+01	2	5	-1
7	1408806191	2.500e+01	2.500e+01	1.250e+01	3	3	-1
7	1408814584	5.000e+01	1.500e+01	1.250e+01	1	3	-1
7	1408819661	1.000e+01	1.000e+01	1.250e+01	3	4	-1
7	1408812882	1.300e+02	2.000e+01	1.250e+01	2	3	-1
7	1408808711	1.500e+01	1.500e+01	1.250e+01	3	3	-1
7	1408801183	3.000e+01	3.000e+01	1.250e+01	4	4	-1
7	1408809769	6.110e+00	6.110e+00	1.250e+01	5	5	-1
7	1408799162	1.300e+01	1.300e+01	1.250e+01	3	3	-1
7	1408805091	2.000e+00	2.000e+00	1.250e+01	3	3	-1
7	1408806651	5.000e+01	2.000e+01	1.250e+01	3	3	-1
7	1408816332	7.000e+00	1.000e+01	1.250e+01	4	4	-1
7	1408809326	1.200e+02	1.200e+02	1.250e+01	3	3	-1
7	1408808791	1.000e+01	1.000e+01	1.250e+01	4	4	-1
7	1408801643	3.000e+00	8.000e+00	1.250e+01	1	1	-1
7	1408807809	8.000e+00	8.000e+00	1.250e+01	4	3	-1
7	1408797111	5.000e+01	2.000e+01	1.250e+01	2	3	-1
7	1408808623	1.500e+01	1.500e+01	1.250e+01	3	2	-1
7	1408800367	3.000e+01	2.000e+01	1.250e+01	2	3	-1
7	1408808143	2.000e+01	2.000e+01	1.250e+01	4	4	-1
7	1408802365	1.000e+01	1.200e+01	1.250e+01	3	4	-1
7	1408812007	5.000e+00	5.000e+00	1.250e+01	3	3	-1
7	1408812647	5.800e+01	5.800e+01	1.250e+01	3	3	-1
7	1408805344	3.000e+00	3.000e+00	1.250e+01	2	2	-1
7	1408816236	5.000e+01	1.500e+01	1.250e+01	2	3	-1
7	1408814110	3.000e+01	2.000e+01	1.250e+01	3	3	-1
7	1408802237	8.000e+00	8.000e+00	1.250e+01	3	3	-1
7	1408811941	5.000e+00	1.000e+01	1.250e+01	3	3	-1
7	1408820913	5.000e+01	4.000e+01	1.250e+01	3	3	-1
7	1408812832	1.500e+01	1.500e+01	1.250e+01	3	3	-1
8	1398776357	1.000e+02	1.000e+02	8.000e+01	1	1	5
8	1398887847	1.000e+02	1.000e+02	8.000e+01	4	4	5
8	1398892516	1.000e+02	1.000e+02	8.000e+01	3	3	5
8	1417185554	3.000e+01	3.000e+01	8.000e+01	5	5	5
8	1398888387	1.500e+01	1.500e+01	8.000e+01	4	4	5
8	1398891149	5.000e+01	5.000e+01	8.000e+01	3	3	5
8	1398776407	1.000e+02	1.000e+02	8.000e+01	3	4	5
8	1398891375	1.000e+02	9.000e+01	8.000e+01	3	4	5

8	1398776634	5.000e+01	8.000e+01	8.000e+01	1	2	5
8	1398786088	8.000e+01	8.000e+01	8.000e+01	4	4	5
8	1398892622	7.200e+01	7.200e+01	8.000e+01	3	4	5
8	1398888074	3.000e+01	4.000e+01	8.000e+01	2	3	5
8	1398867272	2.000e+01	2.000e+01	8.000e+01	2	2	5
8	1398862805	1.000e+02	1.000e+02	8.000e+01	3	3	5
8	1398869031	8.000e+01	8.000e+01	8.000e+01	2	4	5
8	1398889535	1.000e+02	8.000e+01	8.000e+01	1	1	5
8	1398888718	2.000e+02	1.000e+02	8.000e+01	2	3	5
8	1398785751	4.000e+01	5.000e+01	8.000e+01	1	2	5
8	1398889130	5.000e+01	5.000e+01	8.000e+01	2	2	5
8	1398891104	3.500e+01	8.000e+01	8.000e+01	5	5	5
8	1398865276	3.000e+02	7.000e+01	8.000e+01	2	4	5
8	1398888893	1.000e+02	1.000e+02	8.000e+01	3	3	5
8	1398891402	1.200e+02	1.000e+02	8.000e+01	1	3	5
8	1398888834	3.000e+02	8.000e+01	8.000e+01	1	4	5
8	1398784937	1.300e+01	8.100e+01	8.000e+01	1	4	5
8	1398888358	4.000e+01	8.000e+01	8.000e+01	3	3	5
8	1398890003	2.500e+01	8.000e+01	8.000e+01	1	5	5
8	1398891430	9.000e+01	9.000e+01	8.000e+01	4	4	5
8	1398776404	6.000e+01	6.000e+01	8.000e+01	3	4	5
8	1398867266	3.500e+02	1.000e+02	8.000e+01	3	4	5
8	1398778952	1.200e+01	2.000e+01	8.000e+01	4	4	5
8	1398865301	2.000e+02	1.000e+02	8.000e+01	2	3	5
8	1398889925	1.250e+02	1.250e+02	8.000e+01	3	3	5
8	1398776517	2.500e+01	8.000e+01	8.000e+01	2	4	5
8	1408795563	3.000e+01	4.000e+01	8.000e+01	1	3	5
8	1408809584	4.000e+01	4.000e+01	8.000e+01	4	4	5
8	1408805301	3.300e+01	3.300e+01	8.000e+01	3	4	5
8	1408805468	5.000e+01	5.000e+01	8.000e+01	3	3	5
8	1408812462	5.000e+01	8.000e+01	8.000e+01	3	4	5
8	1408808932	1.000e+02	9.000e+01	8.000e+01	3	3	5
8	1408803130	3.000e+01	8.000e+01	8.000e+01	3	4	5
8	1408805005	5.000e+01	8.000e+01	8.000e+01	3	3	5
8	1408810956	4.000e+01	6.000e+01	8.000e+01	2	2	5
8	1408815379	2.500e+01	6.000e+01	8.000e+01	3	3	5
8	1408803877	5.000e+01	5.000e+01	8.000e+01	2	2	5
8	1408806920	7.000e+01	7.000e+01	8.000e+01	5	5	5
8	1408807550	6.000e+01	7.000e+01	8.000e+01	3	3	5
8	1408804745	4.600e+01	4.600e+01	8.000e+01	3	3	5
8	1408807503	5.000e+01	8.000e+01	8.000e+01	3	3	5
8	1408807144	5.000e+01	5.000e+01	8.000e+01	3	3	5
8	1408809849	4.000e+01	8.000e+01	8.000e+01	3	3	5
8	1408803676	3.000e+01	6.000e+01	8.000e+01	3	3	5
8	1408817241	1.000e+02	8.000e+01	8.000e+01	2	3	5
8	1408811947	6.500e+01	6.500e+01	8.000e+01	3	3	5
8	1408811461	8.000e+01	8.000e+01	8.000e+01	3	3	5
8	1408814406	5.000e+01	5.000e+01	8.000e+01	4	4	5
8	1408806191	8.000e+01	8.000e+01	8.000e+01	2	4	5
8	1408814584	1.000e+02	8.000e+01	8.000e+01	0	3	5
8	1408819661	8.000e+01	8.000e+01	8.000e+01	2	3	5
8	1408812882	1.300e+02	8.000e+01	8.000e+01	3	3	5
8	1408808711	5.000e+01	6.500e+01	8.000e+01	3	2	5
8	1408801183	5.000e+01	8.000e+01	8.000e+01	3	4	5
8	1408809769	5.000e+01	5.000e+01	8.000e+01	4	4	5
8	1408799162	4.000e+01	4.000e+01	8.000e+01	4	4	5
8	1408805091	2.500e+01	2.500e+01	8.000e+01	4	4	5
8	1408806651	4.000e+01	6.000e+01	8.000e+01	3	4	5
8	1408816332	8.500e+01	8.000e+01	8.000e+01	1	3	5
8	1408809326	8.000e+01	8.000e+01	8.000e+01	4	4	5
8	1408808791	5.000e+01	5.000e+01	8.000e+01	4	4	5
8	1408801643	8.000e+01	8.000e+01	8.000e+01	3	4	5
8	1408807809	7.000e+01	8.000e+01	8.000e+01	4	4	5
8	1408797111	2.000e+02	8.000e+01	8.000e+01	2	3	5
8	1408808623	5.500e+01	7.000e+01	8.000e+01	2	3	5
8	1408800367	9.000e+01	9.000e+01	8.000e+01	3	4	5
8	1408808143	1.000e+02	1.000e+02	8.000e+01	2	3	5
8	1408802365	1.500e+02	9.000e+01	8.000e+01	3	3	5
8	1408812007	1.000e+02	8.000e+01	8.000e+01	3	5	5

8	1408812647	2.000e+02	2.000e+02	8.000e+01	3	3	5
8	1408805344	3.000e+01	3.000e+01	8.000e+01	3	3	5
8	1408816236	6.000e+01	7.800e+01	8.000e+01	3	4	5
8	1408814110	4.000e+01	7.500e+01	8.000e+01	3	4	5
8	1408802237	3.500e+01	3.500e+01	8.000e+01	4	4	5
8	1408811941	3.500e+01	5.000e+01	8.000e+01	3	3	5
8	1408820913	7.000e+01	7.000e+01	8.000e+01	2	3	5
8	1408812832	7.000e+01	7.000e+01	8.000e+01	4	4	5
9	1398776357	1.000e+02	1.000e+02	1.380e+03	3	3	-1
9	1398887847	2.000e+02	2.000e+02	1.380e+03	2	2	-1
9	1398892516	1.200e+03	1.200e+03	1.380e+03	3	3	-1
9	1417185554	3.000e+02	3.000e+02	1.380e+03	4	1	-1
9	1398888387	5.000e+03	1.500e+03	1.380e+03	4	4	-1
9	1398891149	3.000e+02	5.000e+02	1.380e+03	1	2	-1
9	1398776407	5.000e+02	1.000e+03	1.380e+03	3	3	-1
9	1398891375	1.400e+02	1.400e+02	1.380e+03	1	1	-1
9	1398776634	6.000e+02	1.380e+03	1.380e+03	2	2	-1
9	1398786088	2.000e+02	6.000e+02	1.380e+03	4	3	-1
9	1398892622	3.600e+03	2.300e+03	1.380e+03	4	2	-1
9	1398888074	1.000e+03	1.000e+03	1.380e+03	2	2	-1
9	1398867272	1.800e+02	3.000e+02	1.380e+03	1	3	-1
9	1398862805	1.200e+02	1.000e+03	1.380e+03	1	3	-1
9	1398869031	1.200e+03	1.300e+03	1.380e+03	4	4	-1
9	1398889535	1.600e+02	1.360e+03	1.380e+03	1	1	-1
9	1398888718	3.000e+02	1.300e+03	1.380e+03	2	2	-1
9	1398785751	8.000e+02	1.000e+03	1.380e+03	3	3	-1
9	1398889130	1.000e+03	1.000e+03	1.380e+03	1	1	-1
9	1398891104	3.000e+03	1.800e+03	1.380e+03	3	3	-1
9	1398865276	2.000e+02	2.000e+02	1.380e+03	3	3	-1
9	1398888893	1.200e+02	2.000e+03	1.380e+03	4	4	-1
9	1398891402	7.500e+02	1.200e+03	1.380e+03	0	3	-1
9	1398888834	2.000e+02	1.000e+03	1.380e+03	1	1	-1
9	1398784937	1.500e+03	1.400e+03	1.380e+03	3	3	-1
9	1398888358	1.200e+03	1.380e+03	1.380e+03	2	2	-1
9	1398890003	1.200e+03	1.200e+03	1.380e+03	4	5	-1
9	1398891430	2.750e+02	5.000e+02	1.380e+03	4	4	-1
9	1398776404	9.000e+02	9.000e+02	1.380e+03	2	3	-1
9	1398867266	3.000e+02	1.300e+03	1.380e+03	1	3	-1
9	1398778952	4.500e+01	1.300e+03	1.380e+03	4	4	-1
9	1398865301	1.500e+03	1.500e+03	1.380e+03	1	1	-1
9	1398889925	2.500e+02	6.500e+02	1.380e+03	2	2	-1
9	1398776517	1.500e+02	1.000e+03	1.380e+03	3	3	-1
9	1408795563	9.750e+02	1.300e+03	1.380e+03	4	4	-1
9	1408809584	1.750e+03	1.750e+03	1.380e+03	5	5	-1
9	1408805301	1.450e+03	1.450e+03	1.380e+03	4	5	-1
9	1408805468	7.470e+02	7.470e+02	1.380e+03	5	1	-1
9	1408812462	1.600e+03	1.600e+03	1.380e+03	3	3	-1
9	1408808932	3.000e+02	1.000e+03	1.380e+03	3	3	-1
9	1408803130	7.000e+02	1.380e+03	1.380e+03	2	2	-1
9	1408805005	5.000e+02	1.200e+03	1.380e+03	1	3	-1
9	1408810956	1.350e+03	1.350e+03	1.380e+03	5	5	-1
9	1408815379	9.000e+02	1.200e+03	1.380e+03	3	3	-1
9	1408803877	2.000e+02	1.300e+03	1.380e+03	2	3	-1
9	1408806920	1.500e+03	1.400e+03	1.380e+03	0	0	-1
9	1408807550	1.500e+03	1.400e+03	1.380e+03	3	4	-1
9	1408804745	1.200e+03	1.300e+03	1.380e+03	3	3	-1
9	1408807503	8.000e+02	8.000e+02	1.380e+03	2	2	-1
9	1408807144	1.545e+03	1.545e+03	1.380e+03	5	5	-1
9	1408809849	1.500e+03	1.500e+03	1.380e+03	3	3	-1
9	1408803676	3.500e+02	1.200e+03	1.380e+03	3	3	-1
9	1408817241	3.500e+03	2.500e+03	1.380e+03	3	3	-1
9	1408811947	1.250e+03	1.400e+03	1.380e+03	3	3	-1
9	1408811461	9.000e+02	1.100e+03	1.380e+03	3	3	-1
9	1408814406	1.400e+03	1.380e+03	1.380e+03	4	5	-1
9	1408806191	7.000e+02	1.000e+03	1.380e+03	1	3	-1
9	1408814584	7.000e+02	1.380e+03	1.380e+03	2	3	-1
9	1408819661	1.000e+03	1.000e+03	1.380e+03	3	3	-1
9	1408812882	5.000e+03	3.000e+03	1.380e+03	2	3	-1
9	1408808711	7.500e+02	7.500e+02	1.380e+03	4	4	-1

9	1408801183	1.400e+02	1.300e+03	1.380e+03	3	3	-1
9	1408809769	5.000e+02	1.300e+03	1.380e+03	3	3	-1
9	1408799162	8.000e+02	8.000e+02	1.380e+03	3	3	-1
9	1408805091	1.000e+03	1.000e+03	1.380e+03	2	2	-1
9	1408806651	1.400e+03	1.400e+03	1.380e+03	3	4	-1
9	1408816332	1.000e+03	1.300e+03	1.380e+03	3	3	-1
9	1408809326	1.100e+03	1.200e+03	1.380e+03	3	3	-1
9	1408808791	2.000e+02	2.000e+02	1.380e+03	3	2	-1
9	1408801643	1.000e+03	1.000e+03	1.380e+03	2	2	-1
9	1408807809	1.700e+03	1.380e+03	1.380e+03	4	3	-1
9	1408797111	3.000e+03	1.500e+03	1.380e+03	3	3	-1
9	1408808623	5.000e+02	8.000e+02	1.380e+03	3	2	-1
9	1408800367	1.000e+03	1.200e+03	1.380e+03	2	3	-1
9	1408808143	1.300e+03	1.300e+03	1.380e+03	3	4	-1
9	1408802365	1.500e+03	1.400e+03	1.380e+03	3	3	-1
9	1408812007	1.800e+03	1.800e+03	1.380e+03	3	3	-1
9	1408812647	2.000e+02	1.000e+03	1.380e+03	2	4	-1
9	1408805344	1.530e+03	1.530e+03	1.380e+03	4	4	-1
9	1408816236	1.200e+03	1.380e+03	1.380e+03	2	3	-1
9	1408814110	2.000e+03	1.500e+03	1.380e+03	2	3	-1
9	1408802237	1.200e+03	1.300e+03	1.380e+03	3	4	-1
9	1408811941	1.500e+03	1.500e+03	1.380e+03	3	3	-1
9	1408820913	1.500e+03	1.300e+03	1.380e+03	2	2	-1
9	1408812832	9.900e+02	1.400e+03	1.380e+03	4	4	-1
10	1398776357	1.000e+00	2.000e+02	5.000e+02	2	2	4
10	1398887847	2.500e+01	2.500e+01	5.000e+02	3	3	4
10	1398892516	3.000e+01	4.000e+01	5.000e+02	3	4	4
10	1417185554	1.000e+01	1.000e+01	5.000e+02	0	0	4
10	1398888387	1.000e+01	1.000e+01	5.000e+02	4	4	4
10	1398891149	1.000e+02	1.000e+02	5.000e+02	3	3	4
10	1398776407	1.200e+02	3.000e+02	5.000e+02	4	4	4
10	1398891375	1.000e+02	2.000e+02	5.000e+02	2	2	4
10	1398776634	8.000e+01	8.000e+01	5.000e+02	3	4	4
10	1398786088	1.500e+01	1.500e+01	5.000e+02	3	3	4
10	1398892622	1.500e+01	5.000e+01	5.000e+02	3	2	4
10	1398888074	1.500e+01	3.000e+02	5.000e+02	3	3	4
10	1398867272	1.500e+01	5.000e+01	5.000e+02	3	2	4
10	1398862805	1.500e+01	1.000e+02	5.000e+02	3	3	4
10	1398869031	5.000e+01	6.000e+01	5.000e+02	3	4	4
10	1398889535	3.000e+01	6.000e+01	5.000e+02	2	3	4
10	1398888718	5.000e+00	5.000e+00	5.000e+02	3	3	4
10	1398785751	5.000e+01	5.000e+01	5.000e+02	3	4	4
10	1398889130	5.000e+00	5.000e+00	5.000e+02	2	2	4
10	1398891104	5.000e+01	3.000e+02	5.000e+02	4	4	4
10	1398865276	2.000e+01	5.000e+02	5.000e+02	3	3	4
10	1398888893	3.000e+01	1.000e+02	5.000e+02	3	3	4
10	1398891402	9.000e+01	9.000e+01	5.000e+02	4	4	4
10	1398888834	1.000e+02	3.000e+02	5.000e+02	1	2	4
10	1398784937	1.000e+02	5.000e+02	5.000e+02	1	1	4
10	1398888358	1.400e+01	1.400e+01	5.000e+02	3	3	4
10	1398890003	1.000e+01	2.000e+02	5.000e+02	3	4	4
10	1398891430	2.500e+02	2.500e+02	5.000e+02	4	4	4
10	1398776404	4.000e+01	9.000e+01	5.000e+02	3	3	4
10	1398867266	2.000e+01	2.000e+01	5.000e+02	3	3	4
10	1398778952	5.000e+00	5.000e+00	5.000e+02	4	4	4
10	1398865301	7.500e+02	6.000e+02	5.000e+02	3	3	4
10	1398889925	5.000e+01	5.000e+01	5.000e+02	3	3	4
10	1398776517	5.000e+01	3.000e+02	5.000e+02	3	3	4
10	1408795563	9.000e+01	2.500e+02	5.000e+02	4	4	4
10	1408809584	3.000e+01	3.000e+01	5.000e+02	3	3	4
10	1408805301	6.000e+01	6.000e+01	5.000e+02	4	4	4
10	1408805468	3.000e+01	5.000e+01	5.000e+02	4	4	4
10	1408812462	2.000e+02	2.000e+02	5.000e+02	2	2	4
10	1408808932	4.000e+00	1.000e+01	5.000e+02	3	3	4
10	1408803130	1.200e+02	4.000e+02	5.000e+02	2	2	4
10	1408805005	1.900e+02	1.900e+02	5.000e+02	4	4	4
10	1408810956	5.000e+02	5.000e+02	5.000e+02	2	3	4
10	1408815379	2.000e+01	1.000e+02	5.000e+02	2	3	4
10	1408803877	2.000e+01	2.000e+02	5.000e+02	2	3	4

10	1408806920	2.000e+01	5.000e+02	5.000e+02	3	3	4
10	1408807550	1.500e+01	1.500e+01	5.000e+02	3	3	4
10	1408804745	5.000e+01	5.000e+01	5.000e+02	3	3	4
10	1408807503	5.000e+02	5.000e+02	5.000e+02	3	3	4
10	1408807144	1.000e+02	3.000e+02	5.000e+02	2	2	4
10	1408809849	1.000e+03	1.000e+03	5.000e+02	4	4	4
10	1408803676	2.000e+01	2.500e+01	5.000e+02	0	2	4
10	1408817241	1.000e+02	3.000e+02	5.000e+02	3	3	4
10	1408811947	1.000e+01	5.000e+02	5.000e+02	2	2	4
10	1408811461	3.000e+02	4.000e+02	5.000e+02	3	3	4
10	1408814406	5.000e+01	5.000e+01	5.000e+02	3	3	4
10	1408806191	1.000e+01	1.000e+02	5.000e+02	3	3	4
10	1408814584	2.000e+01	2.000e+02	5.000e+02	2	2	4
10	1408819661	1.500e+01	1.500e+01	5.000e+02	3	3	4
10	1408812882	1.000e+02	2.000e+02	5.000e+02	2	3	4
10	1408808711	8.000e+01	8.000e+01	5.000e+02	4	4	4
10	1408801183	1.500e+01	1.500e+01	5.000e+02	5	4	4
10	1408809769	3.000e+01	3.000e+02	5.000e+02	3	3	4
10	1408799162	2.500e+01	2.500e+01	5.000e+02	4	4	4
10	1408805091	3.000e+00	3.000e+00	5.000e+02	3	3	4
10	1408806651	3.000e+01	3.000e+01	5.000e+02	2	2	4
10	1408816332	3.000e+02	3.000e+02	5.000e+02	3	3	4
10	1408809326	8.000e+01	8.000e+01	5.000e+02	3	3	4
10	1408808791	1.000e+01	5.000e+01	5.000e+02	4	3	4
10	1408801643	1.000e+02	1.000e+02	5.000e+02	1	1	4
10	1408807809	5.000e+01	2.000e+02	5.000e+02	3	3	4
10	1408797111	5.000e+01	2.000e+02	5.000e+02	3	3	4
10	1408808623	1.200e+02	1.200e+02	5.000e+02	3	3	4
10	1408800367	5.000e+01	1.500e+02	5.000e+02	2	3	4
10	1408808143	2.000e+01	5.000e+01	5.000e+02	3	4	4
10	1408802365	3.000e+02	4.000e+02	5.000e+02	1	4	4
10	1408812007	2.500e+01	2.500e+01	5.000e+02	3	3	4
10	1408812647	1.500e+01	1.500e+01	5.000e+02	4	4	4
10	1408805344	2.000e+01	2.000e+01	5.000e+02	4	4	4
10	1408816236	1.000e+01	1.000e+02	5.000e+02	2	3	4
10	1408814110	1.000e+02	2.000e+02	5.000e+02	3	3	4
10	1408802237	1.000e+02	2.000e+02	5.000e+02	2	2	4
10	1408811941	4.000e+01	4.000e+01	5.000e+02	3	3	4
10	1408820913	5.500e+01	5.500e+01	5.000e+02	3	3	4
10	1408812832	8.000e+01	8.000e+01	5.000e+02	4	5	4
11	1398776357	1.000e+00	1.000e+03	1.120e+03	0	0	-1
11	1398887847	1.000e+02	1.000e+03	1.120e+03	2	2	-1
11	1398892516	8.000e+02	1.000e+03	1.120e+03	3	3	-1
11	1417185554	7.500e+02	7.500e+02	1.120e+03	4	4	-1
11	1398888387	5.000e+01	5.000e+02	1.120e+03	2	2	-1
11	1398891149	2.000e+02	2.000e+02	1.120e+03	3	3	-1
11	1398776407	9.000e+01	1.000e+03	1.120e+03	2	2	-1
11	1398891375	1.000e+03	1.120e+03	1.120e+03	2	3	-1
11	1398776634	6.000e+01	1.000e+02	1.120e+03	1	1	-1
11	1398786088	1.500e+02	1.500e+02	1.120e+03	4	4	-1
11	1398892622	4.000e+02	4.000e+02	1.120e+03	2	2	-1
11	1398888074	3.000e+02	2.000e+02	1.120e+03	0	0	-1
11	1398867272	1.500e+02	2.000e+02	1.120e+03	3	4	-1
11	1398862805	1.000e+01	1.000e+03	1.120e+03	0	3	-1
11	1398869031	3.500e+02	6.500e+02	1.120e+03	2	3	-1
11	1398889535	2.000e+02	1.200e+03	1.120e+03	0	0	-1
11	1398888718	1.000e+00	1.200e+02	1.120e+03	1	1	-1
11	1398785751	7.000e+02	9.000e+02	1.120e+03	3	3	-1
11	1398889130	1.000e+02	1.000e+02	1.120e+03	2	2	-1
11	1398891104	1.000e+03	1.300e+03	1.120e+03	2	4	-1
11	1398865276	5.000e+01	1.000e+03	1.120e+03	0	0	-1
11	1398888893	1.500e+03	1.500e+03	1.120e+03	3	3	-1
11	1398891402	6.800e+02	1.000e+03	1.120e+03	1	3	-1
11	1398888834	5.000e+02	1.000e+03	1.120e+03	0	1	-1
11	1398784937	5.000e+02	1.000e+03	1.120e+03	0	0	-1
11	1398888358	1.200e+03	1.200e+03	1.120e+03	3	3	-1
11	1398890003	2.000e+02	1.000e+03	1.120e+03	0	4	-1
11	1398891430	3.500e+02	5.000e+02	1.120e+03	3	3	-1
11	1398776404	7.500e+02	7.500e+02	1.120e+03	3	4	-1

11	1398867266	3.000e+02	1.000e+03	1.120e+03	1	3	-1
11	1398778952	1.500e+02	1.500e+03	1.120e+03	3	3	-1
11	1398865301	3.500e+02	5.000e+02	1.120e+03	2	2	-1
11	1398889925	5.000e+02	5.000e+02	1.120e+03	3	3	-1
11	1398776517	2.000e+01	1.200e+03	1.120e+03	0	3	-1
11	1408795563	1.200e+03	1.200e+03	1.120e+03	4	5	-1
11	1408809584	4.000e+02	4.000e+02	1.120e+03	4	4	-1
11	1408805301	3.000e+02	3.000e+02	1.120e+03	4	4	-1
11	1408805468	8.000e+02	1.100e+03	1.120e+03	4	5	-1
11	1408812462	3.000e+02	1.000e+03	1.120e+03	1	2	-1
11	1408808932	5.000e+02	1.000e+03	1.120e+03	0	3	-1
11	1408803130	1.300e+02	1.300e+02	1.120e+03	3	3	-1
11	1408805005	5.000e+01	5.000e+02	1.120e+03	0	1	-1
11	1408810956	4.500e+02	4.500e+02	1.120e+03	4	3	-1
11	1408815379	3.000e+02	1.000e+03	1.120e+03	3	3	-1
11	1408803877	1.200e+02	1.100e+03	1.120e+03	2	2	-1
11	1408806920	1.500e+02	1.000e+03	1.120e+03	0	0	-1
11	1408807550	3.000e+02	1.000e+03	1.120e+03	1	3	-1
11	1408804745	6.000e+02	6.000e+02	1.120e+03	4	4	-1
11	1408807503	1.200e+02	6.000e+02	1.120e+03	3	2	-1
11	1408807144	8.000e+02	9.000e+02	1.120e+03	2	2	-1
11	1408809849	3.600e+03	3.600e+03	1.120e+03	5	5	-1
11	1408803676	8.000e+02	9.000e+02	1.120e+03	0	2	-1
11	1408817241	9.000e+02	9.000e+02	1.120e+03	3	3	-1
11	1408811947	1.200e+02	1.000e+03	1.120e+03	0	0	-1
11	1408811461	1.200e+03	1.200e+03	1.120e+03	3	3	-1
11	1408814406	6.000e+02	1.100e+03	1.120e+03	4	5	-1
11	1408806191	2.000e+02	2.000e+02	1.120e+03	2	2	-1
11	1408814584	5.000e+02	1.120e+03	1.120e+03	1	1	-1
11	1408819661	1.150e+03	1.150e+03	1.120e+03	4	5	-1
11	1408812882	1.600e+02	3.000e+02	1.120e+03	2	3	-1
11	1408808711	4.000e+02	9.000e+02	1.120e+03	4	3	-1
11	1408801183	1.300e+02	1.000e+03	1.120e+03	0	1	-1
11	1408809769	1.000e+02	1.000e+03	1.120e+03	1	1	-1
11	1408799162	1.200e+03	1.120e+03	1.120e+03	4	4	-1
11	1408805091	1.200e+02	1.000e+03	1.120e+03	4	4	-1
11	1408806651	4.000e+02	6.000e+02	1.120e+03	3	3	-1
11	1408816332	5.000e+02	9.000e+02	1.120e+03	3	3	-1
11	1408809326	1.800e+02	1.800e+02	1.120e+03	3	3	-1
11	1408808791	7.200e+02	7.200e+02	1.120e+03	4	4	-1
11	1408801643	5.000e+02	1.000e+03	1.120e+03	3	4	-1
11	1408807809	1.000e+03	1.000e+03	1.120e+03	4	4	-1
11	1408797111	3.000e+02	5.000e+02	1.120e+03	3	3	-1
11	1408808623	1.000e+03	1.000e+03	1.120e+03	3	3	-1
11	1408800367	1.000e+03	1.000e+03	1.120e+03	4	4	-1
11	1408808143	1.800e+02	1.800e+02	1.120e+03	3	1	-1
11	1408802365	1.100e+03	1.100e+03	1.120e+03	4	4	-1
11	1408812007	8.000e+02	8.000e+02	1.120e+03	3	3	-1
11	1408812647	1.000e+02	1.000e+02	1.120e+03	3	3	-1
11	1408805344	9.500e+02	9.500e+02	1.120e+03	3	3	-1
11	1408816236	7.200e+02	1.000e+03	1.120e+03	3	3	-1
11	1408814110	3.000e+02	1.000e+03	1.120e+03	2	3	-1
11	1408802237	4.500e+02	1.050e+03	1.120e+03	3	4	-1
11	1408811941	1.200e+02	1.200e+02	1.120e+03	3	3	-1
11	1408820913	3.000e+03	1.800e+03	1.120e+03	3	3	-1
11	1408812832	8.000e+02	8.000e+02	1.120e+03	4	4	-1
12	1398776357	1.000e+01	1.000e+01	1.000e+02	3	3	1
12	1398887847	2.000e+00	2.000e+00	1.000e+02	3	3	1
12	1398892516	1.000e+02	1.000e+02	1.000e+02	3	3	1
12	1417185554	1.600e+01	1.600e+01	1.000e+02	2	2	1
12	1398888387	1.000e+03	1.000e+03	1.000e+02	3	3	1
12	1398891149	3.000e+03	2.000e+02	1.000e+02	3	3	1
12	1398776407	1.300e+01	1.300e+01	1.000e+02	2	3	1
12	1398891375	3.000e+00	3.000e+00	1.000e+02	2	3	1
12	1398776634	1.000e+00	1.000e+00	1.000e+02	1	1	1
12	1398786088	1.000e+01	1.000e+01	1.000e+02	3	3	1
12	1398892622	3.000e+00	3.000e+00	1.000e+02	2	2	1
12	1398888074	5.000e+02	5.000e+02	1.000e+02	3	3	1
12	1398867272	3.000e+00	4.000e+00	1.000e+02	2	4	1

12	1398862805	6.000e+00	6.000e+00	1.000e+02	3	3	1
12	1398869031	8.000e+01	8.000e+01	1.000e+02	2	2	1
12	1398889535	2.000e+00	6.000e+00	1.000e+02	1	1	1
12	1398888718	5.000e+00	1.500e+01	1.000e+02	2	2	1
12	1398785751	8.000e+00	8.000e+00	1.000e+02	3	3	1
12	1398889130	5.000e+02	5.000e+02	1.000e+02	2	2	1
12	1398891104	7.000e+01	7.000e+01	1.000e+02	4	4	1
12	1398865276	5.000e+01	5.000e+01	1.000e+02	3	3	1
12	1398888893	5.000e+01	1.200e+02	1.000e+02	2	2	1
12	1398891402	2.500e+00	2.500e+00	1.000e+02	4	4	1
12	1398888834	5.000e+01	7.000e+01	1.000e+02	2	2	1
12	1398784937	1.000e+01	1.000e+01	1.000e+02	0	1	1
12	1398888358	2.000e+00	2.000e+00	1.000e+02	3	3	1
12	1398890003	2.000e+01	2.000e+01	1.000e+02	3	3	1
12	1398891430	1.000e+01	1.000e+01	1.000e+02	4	4	1
12	1398776404	1.000e+00	1.000e+00	1.000e+02	2	4	1
12	1398867266	2.500e+00	1.500e+01	1.000e+02	3	3	1
12	1398778952	1.000e+00	1.000e+02	1.000e+02	4	4	1
12	1398865301	1.500e+01	1.500e+01	1.000e+02	3	3	1
12	1398889925	9.000e+00	9.000e+00	1.000e+02	3	3	1
12	1398776517	1.000e+01	6.000e+00	1.000e+02	0	1	1
12	1408795563	1.000e+01	1.000e+01	1.000e+02	3	4	1
12	1408809584	2.000e+01	2.000e+01	1.000e+02	2	2	1
12	1408805301	1.500e+01	1.500e+01	1.000e+02	0	0	1
12	1408805468	3.500e+01	3.500e+01	1.000e+02	3	3	1
12	1408812462	5.000e+01	5.000e+01	1.000e+02	2	2	1
12	1408808932	2.000e+02	2.000e+02	1.000e+02	3	3	1
12	1408803130	2.500e+01	2.500e+01	1.000e+02	2	2	1
12	1408805005	1.000e+01	1.000e+01	1.000e+02	2	2	1
12	1408810956	4.500e+01	7.000e+01	1.000e+02	3	3	1
12	1408815379	5.000e+00	5.000e+01	1.000e+02	3	3	1
12	1408803877	3.000e+01	3.000e+01	1.000e+02	2	2	1
12	1408806920	5.000e+00	5.000e+00	1.000e+02	0	0	1
12	1408807550	5.000e+01	7.000e+01	1.000e+02	2	3	1
12	1408804745	0.000e+00	1.000e+01	1.000e+02	0	2	1
12	1408807503	2.000e+00	2.000e+00	1.000e+02	2	2	1
12	1408807144	2.500e+01	2.500e+01	1.000e+02	3	3	1
12	1408809849	2.000e+01	2.000e+01	1.000e+02	2	2	1
12	1408803676	3.000e+00	1.000e+01	1.000e+02	2	2	1
12	1408817241	5.000e+00	5.000e+00	1.000e+02	3	3	1
12	1408811947	5.000e+00	3.500e+01	1.000e+02	2	2	1
12	1408811461	1.500e+01	2.000e+01	1.000e+02	3	3	1
12	1408814406	5.000e+01	5.000e+01	1.000e+02	2	2	1
12	1408806191	1.000e+01	1.000e+01	1.000e+02	3	3	1
12	1408814584	1.000e+03	1.000e+03	1.000e+02	0	0	1
12	1408819661	1.000e+00	1.000e+00	1.000e+02	1	1	1
12	1408812882	2.500e+00	2.500e+00	1.000e+02	3	3	1
12	1408808711	1.000e+02	1.000e+02	1.000e+02	3	3	1
12	1408801183	5.000e+01	5.000e+01	1.000e+02	3	3	1
12	1408809769	1.000e+01	1.000e+01	1.000e+02	2	2	1
12	1408799162	5.000e+00	5.000e+00	1.000e+02	3	3	1
12	1408805091	2.000e+00	2.000e+00	1.000e+02	3	3	1
12	1408806651	5.000e+00	5.000e+00	1.000e+02	3	3	1
12	1408816332	5.000e+01	5.000e+01	1.000e+02	1	1	1
12	1408809326	3.000e+00	3.000e+00	1.000e+02	3	3	1
12	1408808791	1.000e+01	2.000e+01	1.000e+02	3	3	1
12	1408801643	1.600e+01	5.000e+01	1.000e+02	2	1	1
12	1408807809	1.500e+01	1.500e+01	1.000e+02	2	2	1
12	1408797111	1.000e+01	1.000e+01	1.000e+02	2	2	1
12	1408808623	3.000e+01	3.000e+01	1.000e+02	1	1	1
12	1408800367	5.000e+02	5.000e+02	1.000e+02	2	2	1
12	1408808143	2.000e+01	5.000e+01	1.000e+02	2	3	1
12	1408802365	4.000e+00	5.000e+00	1.000e+02	1	3	1
12	1408812007	1.000e+01	1.000e+01	1.000e+02	3	3	1
12	1408812647	7.500e+00	1.000e+01	1.000e+02	3	3	1
12	1408805344	1.000e+03	1.000e+02	1.000e+02	3	3	1
12	1408816236	1.000e+01	1.000e+01	1.000e+02	3	3	1
12	1408814110	1.800e+02	1.800e+02	1.000e+02	5	5	1
12	1408802237	2.500e+01	4.000e+01	1.000e+02	3	3	1

12	1408811941	3.500e+01	3.500e+01	1.000e+02	3	3	1
12	1408820913	6.000e+01	8.000e+01	1.000e+02	3	3	1
12	1408812832	1.500e+01	1.500e+01	1.000e+02	3	3	1
13	1398776357	1.000e+01	1.000e+01	1.930e+01	0	0	-1
13	1398887847	3.000e+00	1.500e+01	1.930e+01	0	2	-1
13	1398892516	2.000e+00	1.900e+01	1.930e+01	0	3	-1
13	1417185554	3.000e+01	3.000e+01	1.930e+01	0	0	-1
13	1398888387	7.000e+00	7.000e+00	1.930e+01	0	0	-1
13	1398891149	5.000e+00	5.000e+00	1.930e+01	0	0	-1
13	1398776407	2.000e+01	2.000e+01	1.930e+01	2	2	-1
13	1398891375	1.500e+01	1.500e+01	1.930e+01	0	0	-1
13	1398776634	2.000e+00	3.000e+00	1.930e+01	0	1	-1
13	1398786088	5.000e+00	5.000e+00	1.930e+01	0	0	-1
13	1398892622	3.000e+00	3.000e+00	1.930e+01	0	0	-1
13	1398888074	1.500e+01	1.500e+01	1.930e+01	0	0	-1
13	1398867272	3.000e+00	1.500e+01	1.930e+01	0	2	-1
13	1398862805	3.000e+00	1.900e+01	1.930e+01	0	3	-1
13	1398869031	4.000e+00	4.000e+00	1.930e+01	0	0	-1
13	1398889535	5.000e+00	1.000e+01	1.930e+01	0	0	-1
13	1398888718	3.000e+00	1.500e+01	1.930e+01	0	1	-1
13	1398785751	1.000e+01	1.200e+01	1.930e+01	3	3	-1
13	1398889130	2.000e+01	2.000e+01	1.930e+01	1	1	-1
13	1398891104	5.000e+01	3.000e+01	1.930e+01	0	0	-1
13	1398865276	6.000e+00	1.500e+01	1.930e+01	2	2	-1
13	1398888893	1.200e+01	1.200e+01	1.930e+01	0	0	-1
13	1398891402	1.500e+01	1.500e+01	1.930e+01	1	4	-1
13	1398888834	1.500e+01	2.000e+01	1.930e+01	0	3	-1
13	1398784937	1.500e+01	2.000e+01	1.930e+01	0	1	-1
13	1398888358	4.000e+00	4.000e+00	1.930e+01	2	2	-1
13	1398890003	2.000e+00	1.000e+01	1.930e+01	0	4	-1
13	1398891430	1.000e+01	1.900e+01	1.930e+01	0	2	-1
13	1398776404	1.000e+01	1.500e+01	1.930e+01	0	1	-1
13	1398867266	5.000e+00	1.500e+01	1.930e+01	2	3	-1
13	1398778952	3.000e+02	3.000e+02	1.930e+01	3	3	-1
13	1398865301	3.000e+00	7.000e+00	1.930e+01	0	1	-1
13	1398889925	2.700e+01	2.700e+01	1.930e+01	1	1	-1
13	1398776517	1.500e+01	2.000e+01	1.930e+01	2	4	-1
13	1408795563	3.000e+00	2.000e+01	1.930e+01	4	5	-1
13	1408809584	1.500e+01	2.000e+01	1.930e+01	1	2	-1
13	1408805301	1.200e+01	1.200e+01	1.930e+01	0	0	-1
13	1408805468	0.000e+00	1.900e+01	1.930e+01	0	3	-1
13	1408812462	8.000e+00	8.000e+00	1.930e+01	3	2	-1
13	1408808932	1.500e+01	1.500e+01	1.930e+01	3	3	-1
13	1408803130	8.000e+00	8.000e+00	1.930e+01	3	3	-1
13	1408805005	2.000e+00	1.700e+01	1.930e+01	0	3	-1
13	1408810956	5.000e+00	1.500e+01	1.930e+01	0	1	-1
13	1408815379	4.000e+00	1.500e+01	1.930e+01	0	3	-1
13	1408803877	4.000e+00	1.500e+01	1.930e+01	1	2	-1
13	1408806920	3.000e+01	3.000e+01	1.930e+01	0	0	-1
13	1408807550	4.000e+00	1.500e+01	1.930e+01	0	1	-1
13	1408804745	5.000e+00	1.500e+01	1.930e+01	0	0	-1
13	1408807503	6.000e+00	1.900e+01	1.930e+01	0	1	-1
13	1408807144	4.100e+01	3.000e+01	1.930e+01	0	0	-1
13	1408809849	2.500e+01	2.500e+01	1.930e+01	0	0	-1
13	1408803676	1.500e+01	1.700e+01	1.930e+01	0	0	-1
13	1408817241	7.000e+00	7.000e+00	1.930e+01	4	4	-1
13	1408811947	1.700e+01	1.700e+01	1.930e+01	2	2	-1
13	1408811461	8.000e+00	1.500e+01	1.930e+01	0	1	-1
13	1408814406	6.000e+00	1.900e+01	1.930e+01	0	5	-1
13	1408806191	1.500e+01	1.500e+01	1.930e+01	2	2	-1
13	1408814584	1.000e+01	1.930e+01	1.930e+01	0	1	-1
13	1408819661	8.000e+00	8.000e+00	1.930e+01	0	0	-1
13	1408812882	8.000e+00	1.200e+01	1.930e+01	2	2	-1
13	1408808711	3.000e+00	1.900e+01	1.930e+01	0	3	-1
13	1408801183	2.000e+01	2.000e+01	1.930e+01	0	0	-1
13	1408809769	1.000e+01	1.500e+01	1.930e+01	0	2	-1
13	1408799162	3.000e+01	3.000e+01	1.930e+01	3	3	-1
13	1408805091	1.500e+01	1.500e+01	1.930e+01	4	4	-1
13	1408806651	2.000e+01	2.000e+01	1.930e+01	2	3	-1

13	1408816332	4.000e+00	1.900e+01	1.930e+01	0	0	-1
13	1408809326	1.000e+01	1.400e+01	1.930e+01	3	3	-1
13	1408808791	5.000e+00	5.000e+00	1.930e+01	0	0	-1
13	1408801643	3.000e+00	3.000e+00	1.930e+01	3	0	-1
13	1408807809	1.000e+01	2.000e+01	1.930e+01	0	0	-1
13	1408797111	5.000e+00	1.500e+01	1.930e+01	0	2	-1
13	1408808623	9.000e+00	1.500e+01	1.930e+01	2	3	-1
13	1408800367	2.000e+01	2.000e+01	1.930e+01	1	2	-1
13	1408808143	9.000e+00	1.500e+01	1.930e+01	2	3	-1
13	1408802365	2.000e+01	2.000e+01	1.930e+01	0	4	-1
13	1408812007	4.000e+00	1.900e+01	1.930e+01	0	3	-1
13	1408812647	2.000e+00	2.000e+00	1.930e+01	0	0	-1
13	1408805344	1.500e+01	1.500e+01	1.930e+01	0	0	-1
13	1408816236	6.000e+00	1.800e+01	1.930e+01	1	3	-1
13	1408814110	5.000e+01	2.000e+01	1.930e+01	0	0	-1
13	1408802237	9.000e+00	0.000e+00	1.930e+01	1	0	-1
13	1408811941	1.500e+01	1.500e+01	1.930e+01	2	2	-1
13	1408820913	1.000e+01	2.000e+01	1.930e+01	1	1	-1
13	1408812832	2.000e+00	1.500e+01	1.930e+01	1	3	-1
14	1398776357	1.640e+03	1.648e+03	1.647e+03	1	3	5
14	1398887847	8.000e+02	8.000e+02	1.647e+03	0	0	5
14	1398892516	1.400e+03	1.400e+03	1.647e+03	3	3	5
14	1417185554	1.773e+03	1.773e+03	1.647e+03	4	3	5
14	1398888387	1.798e+03	1.798e+03	1.647e+03	0	2	5
14	1398891149	1.800e+03	1.647e+03	1.647e+03	2	2	5
14	1398776407	1.680e+03	1.580e+03	1.647e+03	2	2	5
14	1398891375	1.580e+03	1.580e+03	1.647e+03	4	4	5
14	1398776634	1.590e+03	1.647e+03	1.647e+03	1	2	5
14	1398786088	1.600e+03	1.650e+03	1.647e+03	3	4	5
14	1398892622	1.683e+03	1.673e+03	1.647e+03	3	4	5
14	1398888074	1.800e+03	1.700e+03	1.647e+03	3	3	5
14	1398867272	1.880e+03	1.700e+03	1.647e+03	1	2	5
14	1398862805	1.600e+03	1.647e+03	1.647e+03	1	5	5
14	1398869031	1.680e+03	1.680e+03	1.647e+03	2	4	5
14	1398889535	1.700e+03	1.647e+03	1.647e+03	0	3	5
14	1398888718	1.592e+03	1.642e+03	1.647e+03	0	1	5
14	1398785751	1.700e+03	1.700e+03	1.647e+03	2	3	5
14	1398889130	1.885e+03	1.885e+03	1.647e+03	2	2	5
14	1398891104	1.850e+03	1.647e+03	1.647e+03	3	4	5
14	1398865276	1.860e+03	1.600e+03	1.647e+03	0	4	5
14	1398888893	1.860e+03	1.648e+03	1.647e+03	2	2	5
14	1398891402	1.680e+03	1.647e+03	1.647e+03	2	4	5
14	1398888834	1.521e+03	1.647e+03	1.647e+03	2	5	5
14	1398784937	1.678e+03	1.647e+03	1.647e+03	1	5	5
14	1398888358	1.580e+03	1.647e+03	1.647e+03	2	2	5
14	1398890003	1.920e+03	1.647e+03	1.647e+03	1	5	5
14	1398891430	1.750e+03	1.700e+03	1.647e+03	3	4	5
14	1398776404	1.820e+03	1.647e+03	1.647e+03	1	4	5
14	1398867266	1.530e+03	1.645e+03	1.647e+03	2	4	5
14	1398778952	1.600e+03	1.610e+03	1.647e+03	3	4	5
14	1398865301	8.600e+02	1.600e+03	1.647e+03	1	3	5
14	1398889925	1.750e+03	1.750e+03	1.647e+03	1	1	5
14	1398776517	1.800e+03	1.640e+03	1.647e+03	3	3	5
14	1408795563	1.785e+03	1.677e+03	1.647e+03	1	3	5
14	1408809584	1.642e+03	1.642e+03	1.647e+03	5	5	5
14	1408805301	1.470e+03	1.500e+03	1.647e+03	1	1	5
14	1408805468	1.600e+03	1.650e+03	1.647e+03	1	4	5
14	1408812462	1.590e+03	1.647e+03	1.647e+03	1	3	5
14	1408808932	1.600e+03	1.647e+03	1.647e+03	1	3	5
14	1408803130	1.779e+03	1.647e+03	1.647e+03	3	4	5
14	1408805005	0.000e+00	1.647e+03	1.647e+03	0	3	5
14	1408810956	1.650e+03	1.650e+03	1.647e+03	3	3	5
14	1408815379	1.590e+03	1.647e+03	1.647e+03	3	5	5
14	1408803877	1.640e+03	1.640e+03	1.647e+03	4	4	5
14	1408806920	1.646e+03	1.647e+03	1.647e+03	3	3	5
14	1408807550	1.642e+03	1.642e+03	1.647e+03	4	5	5
14	1408804745	1.796e+03	1.700e+03	1.647e+03	3	3	5
14	1408807503	1.400e+03	1.647e+03	1.647e+03	0	3	5
14	1408807144	1.650e+03	1.647e+03	1.647e+03	2	4	5

14	1408809849	1.500e+03	1.600e+03	1.647e+03	3	3	5
14	1408803676	1.690e+03	1.650e+03	1.647e+03	0	0	5
14	1408817241	1.590e+03	1.647e+03	1.647e+03	2	5	5
14	1408811947	1.535e+03	1.535e+03	1.647e+03	2	2	5
14	1408811461	1.740e+03	1.650e+03	1.647e+03	3	3	5
14	1408814406	1.400e+03	1.400e+03	1.647e+03	3	3	5
14	1408806191	1.680e+03	1.647e+03	1.647e+03	4	4	5
14	1408814584	1.700e+03	1.647e+03	1.647e+03	0	3	5
14	1408819661	1.495e+03	1.647e+03	1.647e+03	3	3	5
14	1408812882	1.630e+03	1.630e+03	1.647e+03	1	3	5
14	1408808711	1.642e+03	1.642e+03	1.647e+03	5	5	5
14	1408801183	1.642e+03	1.647e+03	1.647e+03	4	5	5
14	1408809769	1.600e+03	1.600e+03	1.647e+03	1	2	5
14	1408799162	1.643e+03	1.643e+03	1.647e+03	5	5	5
14	1408805091	1.800e+03	1.600e+03	1.647e+03	2	2	5
14	1408806651	1.600e+03	1.650e+03	1.647e+03	3	4	5
14	1408816332	1.500e+03	1.630e+03	1.647e+03	4	2	5
14	1408809326	1.657e+03	1.657e+03	1.647e+03	2	2	5
14	1408808791	1.560e+03	1.620e+03	1.647e+03	3	2	5
14	1408801643	1.600e+03	1.600e+03	1.647e+03	1	2	5
14	1408807809	1.742e+03	1.647e+03	1.647e+03	4	4	5
14	1408797111	1.700e+03	1.647e+03	1.647e+03	3	3	5
14	1408808623	1.595e+03	1.647e+03	1.647e+03	2	3	5
14	1408800367	1.642e+03	1.642e+03	1.647e+03	4	4	5
14	1408808143	1.750e+03	1.700e+03	1.647e+03	3	3	5
14	1408802365	1.610e+03	1.647e+03	1.647e+03	1	3	5
14	1408812007	1.740e+03	1.647e+03	1.647e+03	3	5	5
14	1408812647	1.600e+03	1.647e+03	1.647e+03	3	5	5
14	1408805344	1.820e+03	1.647e+03	1.647e+03	3	3	5
14	1408816236	1.642e+03	1.642e+03	1.647e+03	4	4	5
14	1408814110	1.520e+03	1.630e+03	1.647e+03	3	4	5
14	1408802237	1.649e+03	1.649e+03	1.647e+03	3	4	5
14	1408811941	1.457e+03	1.630e+03	1.647e+03	3	3	5
14	1408820913	1.580e+03	1.600e+03	1.647e+03	3	2	5
14	1408812832	1.671e+03	1.671e+03	1.647e+03	4	4	5
15	1398776357	1.200e+01	1.200e+01	3.200e+00	2	2	-1
15	1398887847	5.000e+00	5.000e+00	3.200e+00	2	2	-1
15	1398892516	4.000e+00	4.000e+00	3.200e+00	3	3	-1
15	1417185554	2.000e+00	2.000e+00	3.200e+00	1	1	-1
15	1398888387	3.000e+00	3.000e+00	3.200e+00	3	5	-1
15	1398891149	4.500e-01	4.500e-01	3.200e+00	3	3	-1
15	1398776407	4.000e+00	4.000e+00	3.200e+00	3	3	-1
15	1398891375	7.000e+00	7.000e+00	3.200e+00	1	1	-1
15	1398776634	4.000e+00	4.000e+00	3.200e+00	0	2	-1
15	1398786088	6.000e+00	3.000e+00	3.200e+00	3	3	-1
15	1398892622	4.000e+00	4.000e+00	3.200e+00	5	5	-1
15	1398888074	5.000e+00	5.000e+00	3.200e+00	3	3	-1
15	1398867272	2.000e+00	3.000e+00	3.200e+00	3	4	-1
15	1398862805	5.000e+00	5.000e+00	3.200e+00	2	2	-1
15	1398869031	1.500e+00	1.500e+00	3.200e+00	3	3	-1
15	1398889535	1.000e+00	3.000e+00	3.200e+00	1	1	-1
15	1398888718	6.000e+00	4.000e+00	3.200e+00	2	3	-1
15	1398785751	3.000e+00	3.000e+00	3.200e+00	1	1	-1
15	1398889130	4.800e+01	4.800e+01	3.200e+00	2	2	-1
15	1398891104	3.000e+00	3.000e+00	3.200e+00	4	4	-1
15	1398865276	3.000e+00	3.000e+00	3.200e+00	4	4	-1
15	1398888893	1.500e+00	1.500e+00	3.200e+00	2	2	-1
15	1398891402	2.000e+00	2.500e+00	3.200e+00	2	3	-1
15	1398888834	2.000e+00	2.000e+00	3.200e+00	1	1	-1
15	1398784937	5.000e+00	5.000e+00	3.200e+00	1	1	-1
15	1398888358	6.000e+00	3.500e+00	3.200e+00	2	2	-1
15	1398890003	3.000e+00	3.000e+00	3.200e+00	3	4	-1
15	1398891430	4.000e+00	4.000e+00	3.200e+00	4	4	-1
15	1398776404	3.000e+00	3.000e+00	3.200e+00	2	3	-1
15	1398867266	2.000e+00	3.000e+00	3.200e+00	3	3	-1
15	1398778952	2.000e+00	2.000e+00	3.200e+00	4	4	-1
15	1398865301	1.000e+00	1.000e+00	3.200e+00	3	3	-1
15	1398889925	1.500e+00	1.500e+00	3.200e+00	3	3	-1
15	1398776517	5.000e-01	2.000e+00	3.200e+00	0	2	-1

15	1408795563	2.000e+00	3.000e+00	3.200e+00	3	4	-1
15	1408809584	2.000e+00	2.000e+00	3.200e+00	2	2	-1
15	1408805301	7.000e-01	7.000e-01	3.200e+00	2	3	-1
15	1408805468	5.000e+00	4.000e+00	3.200e+00	2	3	-1
15	1408812462	4.000e+00	4.000e+00	3.200e+00	1	1	-1
15	1408808932	2.000e+00	2.500e+00	3.200e+00	3	3	-1
15	1408803130	1.000e+00	1.000e+00	3.200e+00	2	2	-1
15	1408805005	4.000e+00	4.000e+00	3.200e+00	3	3	-1
15	1408810956	5.000e-01	2.000e+00	3.200e+00	2	3	-1
15	1408815379	1.000e+00	1.800e+00	3.200e+00	2	3	-1
15	1408803877	1.000e+00	1.000e+00	3.200e+00	2	2	-1
15	1408806920	3.000e+00	3.000e+00	3.200e+00	3	3	-1
15	1408807550	1.000e+00	3.000e+00	3.200e+00	2	2	-1
15	1408804745	2.000e+00	2.300e+00	3.200e+00	1	1	-1
15	1408807503	2.000e+00	3.000e+00	3.200e+00	3	3	-1
15	1408807144	2.000e+00	3.000e+00	3.200e+00	1	2	-1
15	1408809849	2.000e+00	3.000e+00	3.200e+00	2	3	-1
15	1408803676	2.000e+00	3.000e+00	3.200e+00	3	3	-1
15	1408817241	3.500e+00	3.500e+00	3.200e+00	4	4	-1
15	1408811947	7.000e+00	4.000e+00	3.200e+00	3	3	-1
15	1408811461	1.000e+00	1.000e+00	3.200e+00	2	2	-1
15	1408814406	3.000e+00	3.000e+00	3.200e+00	0	5	-1
15	1408806191	1.000e+00	1.000e+00	3.200e+00	4	4	-1
15	1408814584	5.000e-01	5.000e-01	3.200e+00	3	3	-1
15	1408819661	1.000e+00	2.000e+00	3.200e+00	1	2	-1
15	1408812882	4.000e+00	4.000e+00	3.200e+00	2	3	-1
15	1408808711	6.000e+00	4.000e+00	3.200e+00	4	4	-1
15	1408801183	2.000e+00	2.000e+00	3.200e+00	3	3	-1
15	1408809769	2.000e+00	2.000e+00	3.200e+00	3	3	-1
15	1408799162	3.000e+00	3.000e+00	3.200e+00	3	3	-1
15	1408805091	2.000e+00	2.000e+00	3.200e+00	3	3	-1
15	1408806651	1.000e+00	2.000e+00	3.200e+00	3	3	-1
15	1408816332	2.000e+00	3.000e+00	3.200e+00	1	1	-1
15	1408809326	2.000e+01	5.000e+00	3.200e+00	0	1	-1
15	1408808791	3.000e+00	3.000e+00	3.200e+00	4	4	-1
15	1408801643	2.000e+00	4.000e+00	3.200e+00	0	0	-1
15	1408807809	3.000e+00	3.000e+00	3.200e+00	2	3	-1
15	1408797111	1.000e+00	1.000e+00	3.200e+00	3	3	-1
15	1408808623	3.000e+00	3.000e+00	3.200e+00	3	3	-1
15	1408800367	4.000e+00	4.000e+00	3.200e+00	1	3	-1
15	1408808143	5.000e-01	5.000e-01	3.200e+00	3	2	-1
15	1408802365	6.000e+00	4.000e+00	3.200e+00	2	4	-1
15	1408812007	3.000e+00	3.000e+00	3.200e+00	3	3	-1
15	1408812647	7.500e+00	6.000e+00	3.200e+00	5	5	-1
15	1408805344	1.500e+00	1.500e+00	3.200e+00	3	3	-1
15	1408816236	1.000e+00	3.000e+00	3.200e+00	3	3	-1
15	1408814110	4.000e-01	1.000e+00	3.200e+00	2	2	-1
15	1408802237	5.000e-01	5.000e-01	3.200e+00	1	1	-1
15	1408811941	8.000e+00	4.000e+00	3.200e+00	3	3	-1
15	1408820913	2.000e+00	2.000e+00	3.200e+00	3	2	-1
15	1408812832	4.000e+00	4.000e+00	3.200e+00	3	3	-1
16	1398776357	5.000e+00	5.000e+00	1.600e+00	1	1	4
16	1398887847	5.000e+00	5.000e+00	1.600e+00	2	2	4
16	1398892516	2.000e+00	2.000e+00	1.600e+00	3	3	4
16	1417185554	6.000e+00	6.000e+00	1.600e+00	3	3	4
16	1398888387	1.000e+01	1.000e+01	1.600e+00	3	3	4
16	1398891149	4.000e+00	2.000e+00	1.600e+00	2	3	4
16	1398776407	2.300e+00	2.300e+00	1.600e+00	3	3	4
16	1398891375	5.000e+00	2.000e+00	1.600e+00	0	0	4
16	1398776634	2.000e+00	2.000e+00	1.600e+00	2	2	4
16	1398786088	4.000e+00	4.000e+00	1.600e+00	4	4	4
16	1398892622	7.000e+00	2.000e+00	1.600e+00	3	3	4
16	1398888074	5.000e+00	2.500e+00	1.600e+00	3	3	4
16	1398867272	2.000e+00	2.000e+00	1.600e+00	3	4	4
16	1398862805	2.000e+00	1.500e+00	1.600e+00	0	3	4
16	1398869031	4.000e+00	2.000e+00	1.600e+00	0	3	4
16	1398889535	3.000e+00	3.000e+00	1.600e+00	3	3	4
16	1398888718	3.000e+00	2.000e+00	1.600e+00	0	1	4
16	1398785751	2.000e+00	2.000e+00	1.600e+00	4	4	4

16	1398889130	1.200e+01	1.200e+01	1.600e+00	2	2	4
16	1398891104	2.000e+00	2.000e+00	1.600e+00	1	1	4
16	1398865276	8.000e+00	6.000e+00	1.600e+00	3	3	4
16	1398888893	3.000e+00	3.000e+00	1.600e+00	3	3	4
16	1398891402	3.000e+00	2.000e+00	1.600e+00	3	3	4
16	1398888834	2.000e+00	2.000e+00	1.600e+00	1	3	4
16	1398784937	3.000e+00	2.000e+00	1.600e+00	0	1	4
16	1398888358	3.000e+00	3.000e+00	1.600e+00	2	0	4
16	1398890003	3.000e+00	3.000e+00	1.600e+00	2	4	4
16	1398891430	3.000e+00	3.000e+00	1.600e+00	2	3	4
16	1398776404	2.000e+00	2.000e+00	1.600e+00	4	4	4
16	1398867266	5.000e+00	2.000e+00	1.600e+00	2	4	4
16	1398778952	2.000e+00	2.000e+00	1.600e+00	3	3	4
16	1398865301	2.000e+00	2.000e+00	1.600e+00	2	3	4
16	1398889925	6.000e+00	6.000e+00	1.600e+00	2	2	4
16	1398776517	1.000e+00	2.000e+00	1.600e+00	3	3	4
16	1408795563	1.500e+00	1.500e+00	1.600e+00	5	5	4
16	1408809584	1.500e+00	1.500e+00	1.600e+00	4	4	4
16	1408805301	3.500e+00	1.000e+00	1.600e+00	3	3	4
16	1408805468	2.000e+00	2.000e+00	1.600e+00	4	5	4
16	1408812462	2.000e+00	1.500e+00	1.600e+00	3	3	4
16	1408808932	2.000e+00	2.000e+00	1.600e+00	3	3	4
16	1408803130	1.000e+00	1.000e+00	1.600e+00	3	3	4
16	1408805005	3.000e+00	2.000e+00	1.600e+00	5	4	4
16	1408810956	1.150e+00	1.150e+00	1.600e+00	4	4	4
16	1408815379	1.500e+00	1.500e+00	1.600e+00	3	4	4
16	1408803877	4.000e+00	2.000e+00	1.600e+00	2	3	4
16	1408806920	3.000e+00	3.000e+00	1.600e+00	3	3	4
16	1408807550	1.000e+00	1.200e+00	1.600e+00	2	3	4
16	1408804745	2.000e+00	2.000e+00	1.600e+00	2	2	4
16	1408807503	1.500e+00	1.500e+00	1.600e+00	4	4	4
16	1408807144	1.000e+00	1.000e+00	1.600e+00	3	4	4
16	1408809849	2.000e+00	2.000e+00	1.600e+00	3	3	4
16	1408803676	2.000e+00	2.000e+00	1.600e+00	2	2	4
16	1408817241	2.000e+00	1.600e+00	1.600e+00	3	3	4
16	1408811947	2.500e+00	2.000e+00	1.600e+00	3	3	4
16	1408811461	2.070e+00	2.070e+00	1.600e+00	4	4	4
16	1408814406	1.000e+00	1.500e+00	1.600e+00	1	1	4
16	1408806191	2.000e+00	2.000e+00	1.600e+00	2	3	4
16	1408814584	2.000e+00	2.000e+00	1.600e+00	1	3	4
16	1408819661	2.000e+00	2.000e+00	1.600e+00	5	5	4
16	1408812882	2.000e+00	2.000e+00	1.600e+00	3	3	4
16	1408808711	2.000e+00	2.000e+00	1.600e+00	3	3	4
16	1408801183	2.000e+00	2.000e+00	1.600e+00	0	2	4
16	1408809769	2.000e+00	2.000e+00	1.600e+00	4	4	4
16	1408799162	1.700e+00	1.700e+00	1.600e+00	4	4	4
16	1408805091	4.000e+00	4.000e+00	1.600e+00	5	5	4
16	1408806651	2.000e+00	2.000e+00	1.600e+00	2	2	4
16	1408816332	2.000e+00	2.000e+00	1.600e+00	3	3	4
16	1408809326	3.500e+00	3.500e+00	1.600e+00	3	3	4
16	1408808791	0.000e+00	2.500e+00	1.600e+00	0	4	4
16	1408801643	3.000e+00	3.000e+00	1.600e+00	4	3	4
16	1408807809	2.000e+00	2.000e+00	1.600e+00	5	5	4
16	1408797111	2.000e+00	2.000e+00	1.600e+00	4	4	4
16	1408808623	3.000e+00	2.000e+00	1.600e+00	3	3	4
16	1408800367	2.500e+00	2.300e+00	1.600e+00	3	3	4
16	1408808143	1.000e+01	1.000e+01	1.600e+00	3	3	4
16	1408802365	3.000e+00	2.000e+00	1.600e+00	2	3	4
16	1408812007	4.000e+00	1.600e+00	1.600e+00	2	4	4
16	1408812647	5.000e+00	3.500e+00	1.600e+00	3	3	4
16	1408805344	2.000e+00	2.000e+00	1.600e+00	4	4	4
16	1408816236	3.000e+00	2.000e+00	1.600e+00	3	3	4
16	1408814110	5.000e+00	2.000e+00	1.600e+00	1	2	4
16	1408802237	3.500e+00	3.500e+00	1.600e+00	2	2	4
16	1408811941	2.500e+00	2.500e+00	1.600e+00	4	4	4
16	1408820913	4.000e+00	3.000e+00	1.600e+00	2	2	4
16	1408812832	2.000e+00	2.000e+00	1.600e+00	4	4	4
17	1398776357	2.650e+02	2.320e+02	2.083e+02	4	5	-1
17	1398887847	2.380e+02	2.380e+02	2.083e+02	3	3	-1

17	1398892516	2.000e+02	2.000e+02	2.083e+02	2	3	-1
17	1417185554	2.160e+02	2.160e+02	2.083e+02	4	4	-1
17	1398888387	1.000e+02	1.500e+02	2.083e+02	3	3	-1
17	1398891149	1.000e+02	1.000e+02	2.083e+02	2	2	-1
17	1398776407	2.060e+02	2.060e+02	2.083e+02	4	5	-1
17	1398891375	3.470e+02	2.080e+02	2.083e+02	4	1	-1
17	1398776634	2.700e+02	2.000e+02	2.083e+02	2	2	-1
17	1398786088	2.800e+02	2.080e+02	2.083e+02	4	4	-1
17	1398892622	2.060e+02	2.060e+02	2.083e+02	5	5	-1
17	1398888074	2.070e+02	2.070e+02	2.083e+02	4	4	-1
17	1398867272	2.160e+02	2.160e+02	2.083e+02	4	4	-1
17	1398862805	1.300e+02	1.300e+02	2.083e+02	2	2	-1
17	1398869031	1.400e+02	1.900e+02	2.083e+02	2	2	-1
17	1398889535	1.000e+03	5.000e+02	2.083e+02	0	1	-1
17	1398888718	6.400e+01	1.640e+02	2.083e+02	2	2	-1
17	1398785751	3.000e+02	2.100e+02	2.083e+02	2	2	-1
17	1398889130	2.060e+02	2.060e+02	2.083e+02	5	5	-1
17	1398891104	4.000e+02	4.000e+02	2.083e+02	3	4	-1
17	1398865276	3.000e+02	3.000e+02	2.083e+02	3	3	-1
17	1398888893	2.500e+02	2.500e+02	2.083e+02	0	0	-1
17	1398891402	2.230e+02	2.100e+02	2.083e+02	5	5	-1
17	1398888834	2.880e+02	2.080e+02	2.083e+02	2	2	-1
17	1398784937	2.500e+02	2.080e+02	2.083e+02	3	4	-1
17	1398888358	2.300e+02	2.080e+02	2.083e+02	2	3	-1
17	1398890003	2.060e+02	2.060e+02	2.083e+02	5	5	-1
17	1398891430	2.500e+02	2.500e+02	2.083e+02	5	5	-1
17	1398776404	3.000e+02	3.000e+02	2.083e+02	2	3	-1
17	1398867266	2.070e+02	2.070e+02	2.083e+02	4	4	-1
17	1398778952	3.650e+02	2.050e+02	2.083e+02	1	3	-1
17	1398865301	2.900e+02	2.900e+02	2.083e+02	3	3	-1
17	1398889925	2.060e+02	2.060e+02	2.083e+02	5	5	-1
17	1398776517	2.070e+02	2.070e+02	2.083e+02	3	4	-1
17	1408795563	2.070e+02	2.070e+02	2.083e+02	4	5	-1
17	1408809584	1.000e+02	2.000e+02	2.083e+02	2	3	-1
17	1408805301	2.000e+02	2.150e+02	2.083e+02	1	1	-1
17	1408805468	2.200e+02	2.200e+02	2.083e+02	5	5	-1
17	1408812462	2.000e+02	2.000e+02	2.083e+02	3	3	-1
17	1408808932	5.000e+01	1.500e+02	2.083e+02	2	3	-1
17	1408803130	1.500e+02	1.500e+02	2.083e+02	3	3	-1
17	1408805005	2.000e+02	2.000e+02	2.083e+02	3	3	-1
17	1408810956	3.500e+02	3.000e+02	2.083e+02	3	3	-1
17	1408815379	1.800e+02	2.000e+02	2.083e+02	3	4	-1
17	1408803877	2.060e+02	2.060e+02	2.083e+02	5	5	-1
17	1408806920	2.060e+02	2.060e+02	2.083e+02	5	5	-1
17	1408807550	2.000e+02	2.000e+02	2.083e+02	2	4	-1
17	1408804745	2.460e+02	2.100e+02	2.083e+02	3	3	-1
17	1408807503	1.000e+02	1.500e+02	2.083e+02	2	2	-1
17	1408807144	2.060e+02	2.060e+02	2.083e+02	4	4	-1
17	1408809849	3.000e+02	2.200e+02	2.083e+02	3	3	-1
17	1408803676	3.000e+02	2.500e+02	2.083e+02	1	1	-1
17	1408817241	2.200e+02	2.100e+02	2.083e+02	4	4	-1
17	1408811947	2.060e+02	2.060e+02	2.083e+02	4	4	-1
17	1408811461	3.160e+02	2.600e+02	2.083e+02	3	3	-1
17	1408814406	2.060e+02	2.060e+02	2.083e+02	5	5	-1
17	1408806191	3.000e+02	3.000e+02	2.083e+02	3	3	-1
17	1408814584	3.000e+02	2.500e+02	2.083e+02	1	1	-1
17	1408819661	2.230e+02	2.100e+02	2.083e+02	3	4	-1
17	1408812882	1.800e+02	1.800e+02	2.083e+02	1	2	-1
17	1408808711	2.060e+02	2.060e+02	2.083e+02	5	5	-1
17	1408801183	3.600e+02	3.150e+02	2.083e+02	4	4	-1
17	1408809769	2.000e+02	2.000e+02	2.083e+02	3	3	-1
17	1408799162	2.090e+02	2.090e+02	2.083e+02	5	5	-1
17	1408805091	2.060e+02	2.060e+02	2.083e+02	5	5	-1
17	1408806651	2.000e+02	2.080e+02	2.083e+02	4	4	-1
17	1408816332	2.060e+02	2.060e+02	2.083e+02	5	5	-1
17	1408809326	2.080e+02	2.080e+02	2.083e+02	4	4	-1
17	1408808791	2.070e+02	2.070e+02	2.083e+02	3	3	-1
17	1408801643	7.000e+02	5.000e+02	2.083e+02	0	1	-1
17	1408807809	2.060e+02	2.060e+02	2.083e+02	4	4	-1

17	1408797111	4.000e+02	3.000e+02	2.083e+02	0	1	-1
17	1408808623	2.060e+02	2.060e+02	2.083e+02	4	4	-1
17	1408800367	2.000e+02	2.000e+02	2.083e+02	1	3	-1
17	1408808143	1.300e+02	1.300e+02	2.083e+02	4	4	-1
17	1408802365	1.020e+02	2.000e+02	2.083e+02	1	3	-1
17	1408812007	2.500e+02	2.500e+02	2.083e+02	0	0	-1
17	1408812647	2.000e+02	2.000e+02	2.083e+02	2	2	-1
17	1408805344	2.000e+02	2.080e+02	2.083e+02	2	3	-1
17	1408816236	2.520e+02	2.100e+02	2.083e+02	3	3	-1
17	1408814110	2.000e+02	2.080e+02	2.083e+02	3	4	-1
17	1408802237	2.100e+02	2.100e+02	2.083e+02	2	4	-1
17	1408811941	2.640e+02	2.060e+02	2.083e+02	4	4	-1
17	1408820913	2.010e+02	2.040e+02	2.083e+02	3	3	-1
17	1408812832	3.000e+02	3.000e+02	2.083e+02	4	5	-1
18	1398776357	5.000e+01	5.000e+01	2.000e+02	4	4	3
18	1398887847	9.000e+01	9.000e+01	2.000e+02	2	2	3
18	1398892516	5.000e+01	7.000e+01	2.000e+02	3	3	3
18	1417185554	6.000e+01	6.000e+01	2.000e+02	4	4	3
18	1398888387	2.000e+01	2.000e+01	2.000e+02	1	1	3
18	1398891149	2.000e+01	1.000e+02	2.000e+02	2	2	3
18	1398776407	1.200e+02	1.200e+02	2.000e+02	4	4	3
18	1398891375	1.200e+02	1.200e+02	2.000e+02	1	1	3
18	1398776634	1.000e+02	3.000e+02	2.000e+02	1	2	3
18	1398786088	1.000e+02	1.500e+02	2.000e+02	4	4	3
18	1398892622	3.000e+01	2.000e+02	2.000e+02	1	1	3
18	1398888074	2.000e+02	2.000e+02	2.000e+02	3	3	3
18	1398867272	7.500e+01	1.000e+02	2.000e+02	0	2	3
18	1398862805	1.200e+02	1.200e+02	2.000e+02	3	3	3
18	1398869031	1.600e+02	1.800e+02	2.000e+02	3	4	3
18	1398889535	1.000e+02	3.000e+02	2.000e+02	1	2	3
18	1398888718	1.500e+02	1.500e+02	2.000e+02	2	3	3
18	1398785751	1.000e+02	1.200e+02	2.000e+02	4	3	3
18	1398889130	5.000e+01	5.000e+01	2.000e+02	3	3	3
18	1398891104	1.500e+02	1.500e+02	2.000e+02	5	5	3
18	1398865276	2.000e+02	2.000e+02	2.000e+02	4	4	3
18	1398888893	1.500e+02	1.500e+02	2.000e+02	3	3	3
18	1398891402	1.120e+02	1.120e+02	2.000e+02	3	3	3
18	1398888834	1.800e+02	1.800e+02	2.000e+02	4	4	3
18	1398784937	1.500e+02	1.500e+02	2.000e+02	3	3	3
18	1398888358	1.200e+02	1.200e+02	2.000e+02	3	3	3
18	1398890003	2.000e+01	1.000e+02	2.000e+02	2	3	3
18	1398891430	1.500e+02	1.500e+02	2.000e+02	4	4	3
18	1398776404	1.500e+02	1.500e+02	2.000e+02	4	4	3
18	1398867266	1.500e+02	1.750e+02	2.000e+02	3	4	3
18	1398778952	1.000e+02	1.000e+02	2.000e+02	3	3	3
18	1398865301	1.500e+02	1.500e+02	2.000e+02	2	2	3
18	1398889925	1.500e+02	1.500e+02	2.000e+02	4	4	3
18	1398776517	1.500e+02	1.500e+02	2.000e+02	3	4	3
18	1408795563	1.500e+02	1.500e+02	2.000e+02	4	4	3
18	1408809584	1.500e+02	1.500e+02	2.000e+02	3	3	3
18	1408805301	4.000e+01	4.000e+01	2.000e+02	2	2	3
18	1408805468	9.000e+01	9.000e+01	2.000e+02	4	4	3
18	1408812462	2.000e+02	2.000e+02	2.000e+02	2	3	3
18	1408808932	1.500e+02	2.000e+02	2.000e+02	3	3	3
18	1408803130	1.000e+02	1.000e+02	2.000e+02	3	3	3
18	1408805005	5.000e+01	7.500e+01	2.000e+02	3	3	3
18	1408810956	1.000e+02	1.000e+02	2.000e+02	3	3	3
18	1408815379	1.000e+02	1.000e+02	2.000e+02	3	3	3
18	1408803877	5.000e+01	2.000e+02	2.000e+02	2	2	3
18	1408806920	1.000e+02	1.000e+02	2.000e+02	3	3	3
18	1408807550	8.000e+01	1.000e+02	2.000e+02	2	3	3
18	1408804745	1.200e+02	1.200e+02	2.000e+02	4	4	3
18	1408807503	1.600e+02	1.600e+02	2.000e+02	3	3	3
18	1408807144	1.000e+02	1.000e+02	2.000e+02	1	3	3
18	1408809849	2.000e+02	2.000e+02	2.000e+02	4	4	3
18	1408803676	3.000e+02	3.000e+02	2.000e+02	4	4	3
18	1408817241	1.500e+02	1.500e+02	2.000e+02	4	4	3
18	1408811947	1.750e+02	2.000e+02	2.000e+02	3	3	3
18	1408811461	1.200e+02	1.300e+02	2.000e+02	3	3	3

18	1408814406	1.500e+02	1.500e+02	2.000e+02	3	3	3
18	1408806191	2.000e+02	2.000e+02	2.000e+02	4	4	3
18	1408814584	1.000e+02	1.500e+02	2.000e+02	3	3	3
18	1408819661	5.000e+01	5.000e+01	2.000e+02	0	0	3
18	1408812882	4.000e+02	4.000e+02	2.000e+02	3	4	3
18	1408808711	1.500e+02	1.500e+02	2.000e+02	4	4	3
18	1408801183	1.500e+02	1.500e+02	2.000e+02	3	4	3
18	1408809769	3.000e+02	3.000e+02	2.000e+02	3	3	3
18	1408799162	2.500e+02	2.500e+02	2.000e+02	4	4	3
18	1408805091	2.000e+01	2.000e+01	2.000e+02	3	3	3
18	1408806651	2.000e+02	2.000e+02	2.000e+02	2	3	3
18	1408816332	1.500e+02	1.500e+02	2.000e+02	3	3	3
18	1408809326	4.000e+02	4.000e+02	2.000e+02	1	1	3
18	1408808791	1.000e+02	1.000e+02	2.000e+02	4	4	3
18	1408801643	1.500e+02	1.500e+02	2.000e+02	2	2	3
18	1408807809	1.500e+02	1.500e+02	2.000e+02	3	3	3
18	1408797111	7.500e+01	7.500e+01	2.000e+02	3	3	3
18	1408808623	1.000e+02	1.000e+02	2.000e+02	3	3	3
18	1408800367	1.500e+02	1.500e+02	2.000e+02	4	4	3
18	1408808143	1.000e+02	1.000e+02	2.000e+02	2	3	3
18	1408802365	1.000e+02	1.000e+02	2.000e+02	4	4	3
18	1408812007	1.000e+02	1.000e+02	2.000e+02	0	0	3
18	1408812647	1.000e+00	1.000e+00	2.000e+02	1	1	3
18	1408805344	2.000e+02	2.000e+02	2.000e+02	2	2	3
18	1408816236	3.000e+02	3.000e+02	2.000e+02	3	3	3
18	1408814110	3.000e+02	2.600e+02	2.000e+02	2	2	3
18	1408802237	1.000e+02	1.200e+02	2.000e+02	2	3	3
18	1408811941	1.500e+02	1.500e+02	2.000e+02	3	3	3
18	1408820913	2.000e+02	2.000e+02	2.000e+02	3	3	3
18	1408812832	1.000e+02	1.000e+02	2.000e+02	3	3	3
19	1398776357	1.500e+01	1.500e+01	4.780e+01	2	2	-1
19	1398887847	3.800e+01	3.800e+01	4.780e+01	2	2	-1
19	1398892516	4.000e+01	4.000e+01	4.780e+01	2	3	-1
19	1417185554	1.000e+01	1.000e+01	4.780e+01	1	0	-1
19	1398888387	3.000e+01	3.000e+01	4.780e+01	3	3	-1
19	1398891149	2.000e+01	3.000e+01	4.780e+01	3	3	-1
19	1398776407	3.000e+01	4.000e+01	4.780e+01	3	3	-1
19	1398891375	3.500e+01	4.000e+01	4.780e+01	2	3	-1
19	1398776634	1.500e+01	5.000e+01	4.780e+01	0	3	-1
19	1398786088	3.500e+01	3.500e+01	4.780e+01	4	4	-1
19	1398892622	1.700e+01	1.700e+01	4.780e+01	2	2	-1
19	1398888074	2.500e+01	3.500e+01	4.780e+01	3	3	-1
19	1398867272	1.700e+01	3.000e+01	4.780e+01	3	3	-1
19	1398862805	2.300e+01	4.700e+01	4.780e+01	1	3	-1
19	1398869031	4.800e+01	4.800e+01	4.780e+01	0	0	-1
19	1398889535	2.000e+01	4.000e+01	4.780e+01	2	2	-1
19	1398888718	1.100e+01	3.100e+01	4.780e+01	2	2	-1
19	1398785751	3.000e+01	3.000e+01	4.780e+01	2	2	-1
19	1398889130	2.000e+01	2.000e+01	4.780e+01	1	1	-1
19	1398891104	2.500e+01	3.500e+01	4.780e+01	4	4	-1
19	1398865276	3.500e+01	4.000e+01	4.780e+01	3	3	-1
19	1398888893	5.700e+01	5.700e+01	4.780e+01	3	3	-1
19	1398891402	6.500e+01	5.000e+01	4.780e+01	4	4	-1
19	1398888834	4.300e+01	4.300e+01	4.780e+01	1	2	-1
19	1398784937	2.500e+01	5.000e+01	4.780e+01	0	0	-1
19	1398888358	4.000e+01	4.000e+01	4.780e+01	2	2	-1
19	1398890003	3.000e+01	4.500e+01	4.780e+01	2	3	-1
19	1398891430	3.500e+01	3.500e+01	4.780e+01	4	4	-1
19	1398776404	4.500e+01	4.500e+01	4.780e+01	2	4	-1
19	1398867266	2.000e+01	3.000e+01	4.780e+01	3	3	-1
19	1398778952	1.500e+01	4.500e+01	4.780e+01	3	3	-1
19	1398865301	2.500e+01	2.500e+01	4.780e+01	3	3	-1
19	1398889925	1.340e+02	1.340e+02	4.780e+01	3	3	-1
19	1398776517	3.000e+00	4.000e+01	4.780e+01	3	2	-1
19	1408795563	1.000e+02	1.000e+02	4.780e+01	3	3	-1
19	1408809584	2.800e+01	3.500e+01	4.780e+01	4	4	-1
19	1408805301	3.000e+01	3.000e+01	4.780e+01	1	1	-1
19	1408805468	3.500e+01	4.200e+01	4.780e+01	3	3	-1
19	1408812462	2.500e+01	4.000e+01	4.780e+01	2	2	-1

19	1408808932	2.000e+01	4.000e+01	4.780e+01	2	3	-1	
19	1408803130	2.300e+01	4.000e+01	4.780e+01	2	2	-1	
19	1408805005	1.700e+01	3.000e+01	4.780e+01	2	3	-1	
19	1408810956	3.500e+01	4.000e+01	4.780e+01	2	2	-1	
19	1408815379	2.200e+01	4.000e+01	4.780e+01	2	3	-1	
19	1408803877	2.000e+01	4.500e+01	4.780e+01	2	2	-1	
19	1408806920	5.400e+01	5.400e+01	4.780e+01	4	4	-1	
19	1408807550	2.000e+01	3.000e+01	4.780e+01	2	3	-1	
19	1408804745	4.600e+01	4.600e+01	4.780e+01	2	2	-1	
19	1408807503	2.500e+01	4.000e+01	4.780e+01	2	2	-1	
19	1408807144	4.000e+01	4.000e+01	4.780e+01	4	4	-1	
19	1408809849	2.700e+01	3.500e+01	4.780e+01	3	2	-1	
19	1408803676	4.000e+01	4.000e+01	4.780e+01	2	2	-1	
19	1408817241	2.000e+01	2.000e+01	4.780e+01	3	3	-1	
19	1408811947	2.600e+01	4.500e+01	4.780e+01	3	3	-1	
19	1408811461	4.700e+01	4.700e+01	4.780e+01	3	3	-1	
19	1408814406	5.000e+01	5.000e+01	4.780e+01	2	2	-1	
19	1408806191	2.000e+01	2.000e+01	4.780e+01	3	3	-1	
19	1408814584	4.000e+01	4.000e+01	4.780e+01	3	3	-1	
19	1408819661	2.500e+01	3.000e+01	4.780e+01	2	2	-1	
19	1408812882	3.200e+01	4.000e+01	4.780e+01	2	3	-1	
19	1408808711	4.000e+01	4.000e+01	4.780e+01	3	3	-1	
19	1408801183	2.500e+01	4.000e+01	4.780e+01	3	3	-1	
19	1408809769	5.000e+01	5.000e+01	4.780e+01	4	4	-1	
19	1408799162	5.000e+01	5.000e+01	4.780e+01	3	3	-1	
19	1408805091	2.600e+01	3.000e+01	4.780e+01	3	3	-1	
19	1408806651	5.000e+01	5.000e+01	4.780e+01	3	3	-1	
19	1408816332	3.500e+01	4.200e+01	4.780e+01	3	2	-1	
19	1408809326	3.200e+01	3.200e+01	4.780e+01	3	3	-1	
19	1408808791	5.000e+01	5.000e+01	4.780e+01	2	3	-1	
19	1408801643	1.500e+01	4.000e+01	4.780e+01	2	1	-1	
19	1408807809	2.500e+01	2.500e+01	4.780e+01	3	3	-1	
19	1408797111	1.500e+01	4.000e+01	4.780e+01	2	3	-1	
19	1408808623	2.500e+01	3.500e+01	4.780e+01	2	2	-1	
19	1408800367	5.000e+01	5.000e+01	4.780e+01	3	3	-1	
19	1408808143	2.000e+01	2.000e+01	4.780e+01	3	3	-1	
19	1408802365	4.400e+01	4.400e+01	4.780e+01	1	4	-1	
19	1408812007	1.500e+01	2.500e+01	4.780e+01	3	3	-1	
19	1408812647	1.800e+01	2.700e+01	4.780e+01	2	2	-1	
19	1408805344	2.500e+01	4.000e+01	4.780e+01	1	2	-1	
19	1408816236	3.000e+01	4.500e+01	4.780e+01	1	3	-1	
19	1408814110	4.500e+01	4.500e+01	4.780e+01	3	3	-1	
19	1408802237	2.500e+01	3.800e+01	4.780e+01	3	3	-1	
19	1408811941	3.500e+01	4.000e+01	4.780e+01	3	3	-1	
19	1408820913	5.000e+01	5.000e+01	4.780e+01	2	3	-1	
19	1408812832	3.000e+01	3.500e+01	4.780e+01	4	5	-1	
20	1398776357	1.000e+03	1.500e+05	1.000e+05	0	2	2	
20	1398887847	3.000e+05	3.000e+05	1.000e+05	2	2	2	
20	1398892516	1.500e+06	5.000e+06	1.000e+05	1	2	2	
20	1417185554	5.000e+03	5.000e+03	1.000e+05	2	2	2	
20	1398888387	5.000e+04	5.000e+04	1.000e+05	0	0	2	
20	1398891149	1.000e+03	3.000e+03	1.000e+05	2	2	2	
20	1398776407	1.000e+04	5.000e+04	1.000e+05	2	2	2	
20	1398891375	1.000e+05	1.000e+05	1.000e+05	0	0	2	
20	1398776634	2.000e+04	2.000e+04	1.000e+05	0	2	2	
20	1398786088	2.500e+04	2.500e+04	1.000e+05	0	0	2	
20	1398892622	5.700e+03	5.700e+03	1.000e+05	2	3	2	
20	1398888074	3.000e+03	3.000e+02	1.000e+05	4	4	2	
20	1398867272	1.200e+03	1.200e+04	1.000e+05	1	3	2	
20	1398862805	2.000e+04	1.000e+05	1.000e+05	1	1	2	
20	1398869031	5.500e+03	5.500e+03	1.000e+05	2	2	2	
20	1398889535	6.000e+03	6.000e+04	1.000e+05	0	2	2	
20	1398888718	1.000e+05	1.000e+05	1.000e+05	0	1	2	
20	1398785751	1.000e+04	1.000e+04	1.000e+05	2	2	2	
20	1398889130	5.000e+04	5.000e+04	1.000e+05	2	2	2	
20	1398891104	3.000e+04	3.000e+04	1.000e+05	1	3	2	
20	1398865276	3.000e+03	3.000e+00	1.000e+05	3	3	2	
20	1398888893	1.355e+04	1.355e+04	1.000e+05	0	0	2	
20	1398891402	1.000e+03	1.000e+06	1.000e+05	3	4	2	

20	1398888834	2.000e+05	2.000e+05	1.000e+05	1	3	2
20	1398784937	9.516e+05	9.516e+05	1.000e+05	0	2	2
20	1398888358	2.000e+07	2.000e+07	1.000e+05	2	2	2
20	1398890003	1.000e+04	1.000e+04	1.000e+05	1	3	2
20	1398891430	1.000e+04	1.000e+04	1.000e+05	3	3	2
20	1398776404	3.000e+04	3.000e+04	1.000e+05	1	2	2
20	1398867266	1.000e+03	1.000e+04	1.000e+05	0	2	2
20	1398778952	1.000e+05	1.000e+05	1.000e+05	3	3	2
20	1398865301	3.000e+05	3.000e+05	1.000e+05	3	3	2
20	1398889925	6.500e+03	6.500e+03	1.000e+05	2	2	2
20	1398776517	1.000e+03	1.500e+05	1.000e+05	2	2	2
20	1408795563	1.000e+05	1.000e+05	1.000e+05	0	4	2
20	1408809584	1.000e+05	1.000e+05	1.000e+05	4	4	2
20	1408805301	5.000e+03	5.000e+03	1.000e+05	1	1	2
20	1408805468	1.300e+04	1.300e+04	1.000e+05	1	1	2
20	1408812462	2.000e+05	2.000e+05	1.000e+05	1	1	2
20	1408808932	8.000e+04	1.000e+05	1.000e+05	3	3	2
20	1408803130	1.000e+04	1.000e+04	1.000e+05	2	2	2
20	1408805005	5.000e+02	1.000e+05	1.000e+05	0	2	2
20	1408810956	1.000e+04	3.000e+04	1.000e+05	1	1	2
20	1408815379	1.000e+05	1.000e+05	1.000e+05	2	3	2
20	1408803877	1.000e+03	1.000e+05	1.000e+05	2	2	2
20	1408806920	2.000e+06	2.000e+06	1.000e+05	0	0	2
20	1408807550	4.000e+04	4.000e+04	1.000e+05	2	2	2
20	1408804745	1.000e+06	1.000e+06	1.000e+05	2	2	2
20	1408807503	5.000e+04	6.000e+04	1.000e+05	1	1	2
20	1408807144	1.000e+05	1.000e+05	1.000e+05	1	1	2
20	1408809849	5.000e+04	5.000e+04	1.000e+05	2	2	2
20	1408803676	1.000e+05	1.000e+05	1.000e+05	1	1	2
20	1408817241	6.000e+03	6.000e+03	1.000e+05	3	3	2
20	1408811947	3.600e+04	1.500e+05	1.000e+05	0	0	2
20	1408811461	1.500e+05	1.500e+05	1.000e+05	3	3	2
20	1408814406	5.000e+04	5.000e+04	1.000e+05	1	1	2
20	1408806191	3.000e+03	3.000e+03	1.000e+05	1	1	2
20	1408814584	5.000e+06	5.000e+06	1.000e+05	0	0	2
20	1408819661	5.000e+03	2.000e+04	1.000e+05	0	0	2
20	1408812882	1.000e+06	1.000e+06	1.000e+05	1	2	2
20	1408808711	2.000e+04	2.000e+04	1.000e+05	3	3	2
20	1408801183	6.000e+05	6.000e+05	1.000e+05	2	3	2
20	1408809769	1.000e+04	1.000e+04	1.000e+05	1	1	2
20	1408799162	2.000e+05	2.000e+05	1.000e+05	2	2	2
20	1408805091	1.000e+04	1.000e+04	1.000e+05	2	2	2
20	1408806651	5.000e+06	4.000e+06	1.000e+05	1	1	2
20	1408816332	1.500e+05	1.500e+05	1.000e+05	4	4	2
20	1408809326	6.500e+04	6.500e+04	1.000e+05	3	3	2
20	1408808791	2.000e+04	2.000e+04	1.000e+05	2	3	2
20	1408801643	1.000e+04	1.000e+05	1.000e+05	0	1	2
20	1408807809	1.000e+06	1.000e+06	1.000e+05	2	2	2
20	1408797111	3.000e+06	3.000e+06	1.000e+05	3	3	2
20	1408808623	5.000e+04	5.000e+04	1.000e+05	2	2	2
20	1408800367	1.000e+04	1.000e+04	1.000e+05	3	3	2
20	1408808143	2.000e+06	2.000e+06	1.000e+05	3	1	2
20	1408802365	1.200e+05	1.200e+05	1.000e+05	1	1	2
20	1408812007	1.000e+04	1.000e+04	1.000e+05	0	3	2
20	1408812647	6.000e+04	6.000e+04	1.000e+05	1	1	2
20	1408805344	3.000e+05	3.000e+05	1.000e+05	1	1	2
20	1408816236	1.000e+04	5.000e+04	1.000e+05	1	3	2
20	1408814110	2.000e+04	1.500e+04	1.000e+05	0	0	2
20	1408802237	3.400e+03	1.000e+04	1.000e+05	2	2	2
20	1408811941	8.000e+23	8.000e+23	1.000e+05	3	3	2
20	1408820913	3.500e+03	3.500e+03	1.000e+05	3	3	2
20	1408812832	1.000e+04	8.000e+04	1.000e+05	2	3	2
