# Supplementary material for: Opinion Formation by Social Influence: From Experiments to Modeling
Source: PLoS One. 2015 Oct 30;10(10):e0140406. doi: 10.1371/journal.pone.0140406 (PMC4627778; doi:10.1371/journal.pone.0140406)
Supplement: S2 Table — (PDF) [file pone.0140406.s003.pdf]

S2 Table. Frequencies of confidence changes measured in the experiment.

**confidence change Δc**	**frequency**
-5	0.000
-4	0.000
-3	0.000
-2	0.005
-1	0.021
0	0.711
1	0.161
2	0.066
3	0.028
4	0.008
5	0.000
