# Supplementary material for: Opinion Formation by Social Influence: From Experiments to Modeling
Source: PLoS One. 2015 Oct 30;10(10):e0140406. doi: 10.1371/journal.pone.0140406 (PMC4627778; doi:10.1371/journal.pone.0140406)
Supplement: S1 Text — (PDF) [file pone.0140406.s004.pdf]

S1 Text. List of questions used in the experiment. The original text in Spanish and an English translation are provided, with a short explanation when necessary. The group to which each question belongs (A or B) is also indicated.

1. *¿Cuántos discos de estudio grabó la banda británica "The Beatles"?* / How many studio records did the British band "The Beatles" publish? (B)
2. *¿Cuántos kilómetros en línea recta separan la ciudad de Viedma de Buenos Aires?* / What is the straight-line distance in kilometers between the city of Viedma an Buenos Aires? (Viedma is the capital of an Argentine province; A)
3. *¿Cuántos centímetros de largo mide una cuerda en una guitarra criolla del puente al clavijero?* / What is the length in centimeters of a classical guitar string from the bridge to the head? (Question accompanied by a figure of a guitar with an arrow indicating the asked length; B)
4. *¿Cuántos metros de largo tiene una cinta "scotch" en todo el carretel?* / What is the length in meters of a Scotch tape roll? (Question accompanied by a photo of a tape roll; A)
5. *¿Cuántas galletitas tiene un paquete de "Cerealitas"?* / How many crackers are there in a Cerealitas pack? (Cerealitas is a very popular Argentine trademark of cereal crackers; B)
6. *¿Cuántos kilos de arroz cocido se preparan con medio kilo de arroz blanco crudo?* / How many kilograms of cooked rice result from half kilogram of raw white rice? (A)
7. *¿Cuántos gramos pesa una moneda de $1 Argentino?* / What is the weight in grams of an Argentine peso coin? (The peso coin's weight has remained the same since several decades; B)
8. *¿Cuántos metros de alto mide el Obelisco de Buenos Aires?* / What is the height in meters of the Buenos Aires Obelisk? (The Buenos Aires Obelisk is an icon of the Argentine capital; A)
9. *¿A qué temperatura (°C) funde el hierro?* / At what temperature in degrees centigrade does iron melt? (B)

10. *En promedio, ¿cuántos litros de agua gasta una persona adulta al bañarse?* / On the average, how many liters of water does an adult use during a bath? (A)

11. *¿A cuántos km/h viaja una bala disparada de un revólver reglamentario 9mm?* / What is the speed in kilometers per hour of a 9mm revolver bullet? (B)

12. *En promedio, ¿cuántas toneladas pesa una ballena azul adulta?* / On the average, how many metric tons does an adult blue whale weight? (A)

13. *¿Cuántas películas realizó como director Stanley Kubrick?* / How many films did Stanley Kubrick direct? (B)

14. *¿En que año nació Isaac Newton?* / What was Isaac Newton's birth year? (A)

15. *¿Cuántas horas dura en promedio una partida de ajedrez en un torneo internacional?* / What is the average duration in hours of a chess match in an international tournament? (B)

16. *¿Cuántas horas duran en promedio los vuelos de cabotaje en la Argentina?* / What is the average duration in hours of a domestic flight in Argentina? (A)

17. *¿Cuántos huesos tiene el esqueleto de un ser humano?* / How many bones does the human skeleton have? (B)

18. *En promedio, ¿cuántos años vive una tortuga de las Islas Galápagos?* / On the average, how many years does a Galapagos turtle live? (A)

19. *¿Cuántos países hay en el continente africano?* / How many countries are there in the African continent? (B)

20. *¿Cuántas palabras tiene el diccionario de la Real Academia Española?* / How many words does the dictionary of the Royal Spanish Academy contain? (The RSA dictionary is the standard reference of the Spanish language; A)
