# Supplementary material for: Opinion Formation by Social Influence: From Experiments to Modeling
Source: PLoS One. 2015 Oct 30;10(10):e0140406. doi: 10.1371/journal.pone.0140406 (PMC4627778; doi:10.1371/journal.pone.0140406)
Supplement: S2 Text — (PDF) [file pone.0140406.s005.pdf]

S2 Text. Analytical model for the autonomous evolution of confidence. Between two successive interaction events, the confidence level changes as $c' = c + \Delta c$ (see the second of Eqs. (2)). Therefore, the probability P'_c of getting a given value of c' depends on the probability P_c of starting from confidence c and the probability $p_{\Delta c}$ of a confidence change $\Delta c = c' - c$. We assume that the transition probabilities $p_{\Delta c}$ do not vary as the process goes on, and that they do not depend on the initial confidence level. Recall also that, in a large population, P_c can be interpreted as the fraction of the population with confidence level c or, equivalently, the frequency of each confidence level.

The requirement that the confidence level must be bounded between 0 and 5 can be insured by fixing suitable boundary conditions. Here, we impose that if the chosen confidence changes lead to $c' < 0$ or $c' > 5$, the resulting confidence is respectively assigned to $c' = 0$ and $c' = 5$. With this prescription the evolution equations for the probabilities P_c read

$$\begin{aligned}
P'_0 = {} & (p_{-5} + p_{-4} + p_{-3} + p_{-2} + p_{-1} + p_0)P_0 \\
& + (p_{-5} + p_{-4} + p_{-3} + p_{-2} + p_{-1})P_1 \\
& + (p_{-5} + p_{-4} + p_{-3} + p_{-2})P_2 \\
& + (p_{-5} + p_{-4} + p_{-3})P_3 + (p_{-5} + p_{-4})P_4 + p_{-5}P_5 \\
P'_1 = {} & p_1 P_0 + p_0 P_1 + p_{-1} P_2 + p_{-2} P_3 + p_{-4} P_4 + p_{-4} P_5 \\
P'_2 = {} & p_2 P_0 + p_1 P_1 + p_0 P_2 + p_{-1} P_3 + p_{-2} P_4 + p_{-3} P_5 \\
P'_3 = {} & p_3 P_0 + p_2 P_1 + p_1 P_2 + p_0 P_3 + p_{-1} P_4 + p_{-2} P_5 \\
P'_4 = {} & p_4 P_0 + p_3 P_1 + p_2 P_2 + p_1 P_3 + p_0 P_4 + p_{-1} P_5 \\
P'_5 = {} & p_5 P_0 + (p_5 + p_4)P_1 + (p_5 + p_4 + p_3)P_2 \\
& + (p_5 + p_4 + p_3 + p_2)P_3 + (p_5 + p_4 + p_3 + p_2 + p_1)P_4 \\
& + (p_5 + p_4 + p_3 + p_2 + p_1 + p_0)P_5
\end{aligned}$$

This system of master-like equations is linear and autonomous, and thus can be exactly solved. As stated in the main text, we estimate the values of $p_{\Delta c}$ from the experimental results as the frequencies observed for each Δc.

First, the stationary confidence distribution is obtained from the equations $P'_c = P_c$ for all c. This yields the values displayed in Table 1. Second, the largest eigenvalues of the linear system characterize the typical time scales of the evolution. In our case, the

maximal eigenvalue is $\lambda_0 = 1$, as required by the conservation of the total probability $P_0 + \cdots + P_5 = 1$. The second largest eigenvalue is $\lambda_1 \approx 0.836$. The dominant contribution to the approach to the stationary distribution depends on the number n of events per agent as λ_1^n. Thus, the distance to the stationary state decreases by a factor ν in $n_\nu = -\ln \nu / \ln \lambda_1$ events per agent. For $\nu = 2$ and 10 we respectively find $n_2 = 3.9$ and $n_{10} = 12.9$.
