# Supplementary material for: Opinion Formation by Social Influence: From Experiments to Modeling
Source: PLoS One. 2015 Oct 30;10(10):e0140406. doi: 10.1371/journal.pone.0140406 (PMC4627778; doi:10.1371/journal.pone.0140406)
Supplement: S3 Text — (PDF) [file pone.0140406.s006.pdf]

S3 Text. Analytical approximation to the evolution of opinions. As explained in the main text, the implementation of the first of Eqs. (2) as a model of opinion evolution requires calculating the influence factor I_{ij} for each interaction event between agents i and j. In turn, the influence factor depends on the confidence difference between the two agents, which changes as the process goes on. An approximation that makes possible the analytical treatment of the first of Eqs. (2) is to assume that confidence has already reached its asymptotic distribution and, moreover, that all agents have the maximal confidence, so that the confidence difference is $\delta c = 0$ for all agent pairs. Under this assumption the probability distribution for the influence factor is estimated from the frequencies calculated for each category, f_K, f_A, and f_C, using the analytical approximations given in Fig. 2B for $\delta c = 0$.

Averaging the first of Eqs. (2) over the population of agents, we find

$$\langle r' \rangle = \langle r \rangle$$

which implies that in our model the average opinion is preserved during the process. A similar calculation shows that the standard deviation of opinions changes as

$$\sigma'_r = (1 - 2\langle I \rangle + 2\langle I^2 \rangle)^{1/2} \sigma_r$$

where the averages of the influence factor and its square are calculated over the respective probability distribution. Using the probability distribution estimated for $\delta c = 0$, we find $\sigma'_r \approx 0.958\, \sigma_r$. As a consequence, the standard deviation approximately halves each 16 events per agent or, equivalently, decreases by a factor of 10 every 54 events per agent. Thus, the convergence of opinions is considerable slower than that of confidence levels, which justifies the approximation of a stationary confidence distribution. For asymptotically long times, all agents share the same opinion, which coincides with the initial value of $\langle r \rangle$.

In the presence of M opinion leaders, whose special role in the dynamics is defined in the main text, the first of Eqs. (2) is replaced by the following set of probabilistic dynamical rules:

$$r'_i = \begin{cases} r_i + I_{im}(R_m - r_i) & \text{with probability } \alpha_m, \quad m = 1, \ldots, M \\ r_i + I_{ij}(r_j - r_i) & \text{with probability } \alpha_0 \end{cases}$$

and $\alpha_0 = 1 - \sum_m \alpha_m$. The first line describes the interaction of agent i with opinion leader m $(= 1, \ldots, M)$, which occurs with frequency α_m and is characterized by an

influence factor I_{im}. The fixed opinion of leader m is R_m. The second line correspond to the interaction with an ordinary agent j.

In a continuous-time approximation the above equation predict that the average opinion depends on the number of events per agent, n, as

$$\langle r\rangle = \bar{R} + (\langle r\rangle_{\text{ini}} - \bar{R})\exp(-n/n_T)$$

where $\langle r\rangle_{\text{ini}}$ is the initial average opinion, and

$$\bar{R} = \frac{\sum_m \alpha_m \langle I\rangle_m R_m}{\sum_m \alpha_m \langle I\rangle_m} \qquad n_T = \left(\sum_m \alpha_m \langle I\rangle_m\right)^{-1}$$

Thus, the average opinion converges to $\bar{R}$, which is in turn an average of the leaders' opinions, each of them weighted by the factor $\alpha_m\langle I\rangle_m$. The inverse of the sum of all these weights, n_T, is the typical number of events per agent for convergence to take place.

Along the same lines it is possible to find the evolution of the standard deviation of opinions. For brevity we only report the stationary standard deviation, which is attained after an asymptotically long number of events:

$$\sigma_{\text{st}} = \left[\frac{\sum_m \alpha_m \langle I^2\rangle_m (\bar{R} - R_m)^2}{2\alpha_0(\langle I\rangle - \langle I^2\rangle) + \sum_m \alpha_m (2\langle I\rangle_m - \langle I^2\rangle_m)}\right]^{1/2}$$
